# Phylogenetic analysis, structural evolution and functional divergence of the 12-oxo-phytodienoate acid reductase gene family in plants

**DOI:** 10.1186/1471-2148-9-90

**Published:** 2009-05-05

**Authors:** Wenyan Li, Bing Liu, Lujun Yu, Dongru Feng, Hongbin Wang, Jinfa Wang

**Affiliations:** 1 State Key Laboratory for Biocontrol and Key Laboratory of Gene Engineering of Ministry of Education, School of Life Sciences, Sun Yat-sen University, 510275, Guangzhou, PR China

## Abstract

**Background:**

The 12-oxo-phytodienoic acid reductases (OPRs) are enzymes that catalyze the reduction of double-bonds in *α*, *β*-unsaturated aldehydes or ketones and are part of the octadecanoid pathway that converts linolenic acid to jasmonic acid. In plants, OPRs belong to the old yellow enzyme family and form multigene families. Although discoveries about this family in *Arabidopsis *and other species have been reported in some studies, the evolution and function of multiple OPRs in plants are not clearly understood.

**Results:**

A comparative genomic analysis was performed to investigate the phylogenetic relationship, structural evolution and functional divergence among OPR paralogues in plants. In total, 74 OPR genes were identified from 11 species representing the 6 major green plant lineages: green algae, mosses, lycophytes, gymnosperms, monocots and dicots. Phylogenetic analysis showed that seven well-conserved subfamilies exist in plants. All OPR genes from green algae were clustered into a single subfamily, while those from land plants fell into six other subfamilies, suggesting that the events leading to the expansion of the OPR family occurred in land plants. Further analysis revealed that lineage-specific expansion, especially by tandem duplication, contributed to the current OPR subfamilies in land plants after divergence from aquatic plants. Interestingly, exon/intron structure analysis showed that the gene structures of OPR paralogues exhibits diversity in intron number and length, while the intron positions and phase were highly conserved across different lineage species. These observations together with the phylogenetic tree revealed that successive single intron loss, as well as indels within introns, occurred during the process of structural evolution of OPR paralogues. Functional divergence analysis revealed that altered functional constraints have occurred at specific amino acid positions after diversification of the paralogues. Most notably, significant functional divergence was also found in all pairs, except for the II/IV, II/V and V/VI pairs. Strikingly, analysis of the site-specific profiles established by posterior probability revealed that the positive-selection sites and/or critical amino acid residues for functional divergence are mainly distributed in *α*-helices and substrate binding loop (SBL), indicating the functional importance of these regions for this protein family.

**Conclusion:**

This study highlights the molecular evolution of the OPR gene family in all plant lineages and indicates critical amino acid residues likely relevant for the distinct functional properties of the paralogues. Further experimental verification of these findings may provide valuable information on the OPRs' biochemical and physiological functions.

## Background

Plant responses to many biotic and abiotic stresses are orchestrated locally and systemically by signaling molecules known as jasmonates (JAs), which are derived from linolenic acid via the octadecanoid pathway [1,2]. Jasmonic acid (JA) and other octadecanoids act as plant growth regulators in various developmental processes such as fruit ripening, pollen maturation, root growth and tendril coiling [3-5]. They are also potent modulators of defenses against insects and pathogens [2,5-7]. Thus, the lipid-based octadecanoid pathway leading to JA has also been found to be an integral part of the signal transduction pathway. The 12-oxo-phytodienoic acid reductases (OPRs) are enzymes that catalyze the reduction of double-bonds adjacent to an oxo group in *α*, *β*-unsaturated aldehydes or ketones and are part of the octadecanoid pathway that converts linolenic acid to jasmonic acid [8,9].

In plants, the OPR genes, which belong to the old yellow enzyme (OYE) family, are flavin mononucleotide (FMN)-dependent oxidoreductases and form multigene families. The first member of the OPR family in higher plants was identified from *Arabidopsis thaliana*, and named *AtOPR1 *[10]. Subsequently, other OPR genes were identified in the tomato [11,12], pea [13], rice [14-16] and maize [17] genomes. Earlier studies on the enzymatic activity of OPRs in *Arabidopsis *and tomato revealed that these enzymes have distinct substrate preferences and therefore have been classified into two groups, group I and II, depending on their substrate specificity [10,12,18,19]. OPR group I enzymes preferentially catalyze the reduction of (9R,13R)-12-oxo-10, 15(Z)-octadecatrienoic acid (9R,13R-OPDA), while OPR group II enzymes preferentially catalyze (9S,13S)-12-oxo-10, 15(Z)-octadecatrienoic acid (9S,13S-OPDA), a natural biosynthetic intermediate precursor in JA biosynthesis. AtOPR3 and LeOPR3, belonging to group II, have been shown to efficiently reduce the natural isomer 9S,13S-OPDA to 3-oxo-2(2'(Z)-pentenyl)-cyclopentane-1-octanoic acid (OPC 8:0), the precursor of JA [12,18,19]. In contrast, AtOPR1/2 and LeOPR1/2, belonging to group I, were unable to catalyze this step [10,18,20].

The biological significance of plants having multiple OPRs is not clearly understood. To date, studies of the physiological role of OPRs have focused mainly on their expression in dicots and monocots. Among dicots, OPRs have been characterized in *Arabidopsis *and tomato. The expression levels of OPR mRNA and protein have also been analyzed in transgenic plants. OPRs in *Arabidopsis *have been shown to have tissue-specific expression patterns. *AtOPR3 *is transcribed more actively in flowers or anthers than in the roots and leaves [21]. Conversely, *AtOPR1/2 *is transcribed more actively in roots and leaves [20]. Furthermore, the transcription of OPRs can also be induced by wounds, pathogens, signaling molecules such as JA, methyl jasmonate (MeJA), salicylic acid (SA), abscisic acid (ABA) and ethylene, and other environmental stimuli [10,20-22]. For example, *AtOPR1/2 *transcription is up-regulated transiently in response to wounding [20]; likewise, the expression of *β*-glucuronidase (GUS) under the control of the *AtOPR1 *and *AtOPR2 *promoters was up-regulated after stimulation by touch, wounding, and ultraviolet (UV) irradiation [20]. In addition, mutants for *AtOPR3*, which encodes the enzymes belonging to group II, were shown to be deficient in the biosynthesis of JA and the males were sterile [21,22]. Moreover, the *AtOPR3 *mutants accumulated 12-oxo-10, 15(Z)-octadecatrienoic acid (OPDA) following wounding and were resistant to fungal and insect attacks [21].

In monocots, more than 13 OPR genes have been found in the rice genome; *OsOPR1 *was the first OPR gene characterized at the biochemical and molecular level [14]. This gene is rapidly and transiently up-regulated in response to a variety of environmental cues including JA, SA, ethylene and H_2_O_2 _[14]. Similar results were found for the expression of *OsOPR7 *[23]. Moreover, over-expression *OsOPR7*, clustered in the same group II, was able to compensate for the phenotype of *AtOPR3 *mutants, whereas *OsOPR1*, which is clustered in other groups, was unable to compensate for the same phenotype [23]. In addition, an analysis of mRNA transcripts indicated that maize OPR genes exhibit organ-specific expression and can be rapidly and transiently up-regulated in response to a variety of biotic and abiotic stresses that include wounding, signaling molecules (JA, MeJA, SA, ABA and ethylene) and the presence of pathogens [17]. In spite of ongoing studies, the function of OPRs in plants remains obscure.

Although biochemical and genetic studies in *Arabidopsis *and other species have led to important discoveries in understanding the function of OPRs, proven biological roles have been elucidated for only a few members of this family and in a limited number of species. Additionally, differences in family size among eukaryotes raise several questions regarding the evolution and functional divergence of the OPR gene family. Thus, a comprehensive comparative genome study is essential for understanding the evolution and function of the OPR gene family in plants. Here, we performed a comparative genomic analysis using a comprehensive bioinformatics/phylogenetic approach to elucidate the evolutionary history, structural evolution and putative functional divergence of the OPR gene family in plants. Firstly, we identified all OPR paralogues from eleven species (*Chlamydomonas reinhardtii*, *Volvox carteri*, *Physcomitrella patens*, *Selaginella moellendorffii*, *Picea sitchensis*, *Oryza sativa*, *Sorghum bicolor, Zea mays, Arabidopsis thaliana*, *Populus trichocarpa *and *Medicago truncatula*), representing the six major plant lineages with available genome sequences. Secondly, phylogenetic analysis was performed to trace back the evolutionary history of the OPR family in plants. Thirdly, exon/intron structure analysis was performed to gain insight into the possible mechanisms for structural evolution of the OPR gene family, because the exon/intron structural divergence within gene families is also a mechanism for the evolution of multiple gene families. Finally, functional divergence analysis suggests that changes in selective constraints and/or amino acid properties occurred after gene duplication, which led to subfamily-specific functional evolution after their diversification. This has also led us to predict the positive-selection sites or critical amino acid sites that may be of importance for the functional divergence of the OPR paralogues.

## Results

### Identification of OPR genes and their homologues in plants

Using the TIGR, TAIR, MaizeGDB, PlantGDB, JGI and NCBI databases, we first retrieved the available OPR or OPR-like sequences from currently sequenced and unfinished genomes; 105 OPR homologue genes were identified (Additional files 1, 2) from various green plants, including unicellular and multicellular green algae, mosses, lycophytes, gymnosperms and angiosperms. To explore the origin and evolutionary history of the OPR gene family, we characterized OPR genes from eleven species representing the six major plant lineages: the green algae *Chlamydomonas reinhardtii *and *Volvox carteri*, the moss *Physcomitrella patens*, the lycophyte *Selaginella moellendorffii*, the gymnosperm *Picea sitchensis*, the monocotyledonous angiosperms *Oryza sativa*, *Sorghum bicolor *and *Zea mays *and the dicotyledonous angiosperms *Arabidopsis thaliana*, *Populus trichocarpa *and *Medicago truncatula*. A complete or draft genome sequence was used in all of our searches, except for the gymnosperm *Picea sitchensis*, whose genome sequence is not yet available. After exclusion of unfinished and partial protein sequences, we finally obtained 74 OPR genes from the above eleven representative plants (Table 1; Additional file 1). The results of Pfam and SMART analysis showed that the typical OPR proteins possess only one Oxidored_FMN (PF00724) domain. Three OPR candidates (AtOPR01-2/3 and SbOPR06-4) without complete Oxidored_FMN domains were excluded from the following analysis.

**Table 1:** Number of OPR or OPR-like genes in 11 representative plants

**Lineage**	**Organism**	Genome Size* (Mbp)	**Number**	**Nomenclature**
Algae	*Chlamydomonas reinhardtii*	121	3	*CrOPR*
	*Volvox carteri*	120	2	*VcOPR*
Mosses	*Physcomitrella patens*	511	6	*PpOPR*
Lycophytes	*Selaginella moellendorffii*	100	6	*SmOPR*
Gymnosperms	*Picea sitchensis*	NA	3	*PsOPR*
Monocots	*Oryza sativa*	430	13	*OsOPR*
	*Sorghum bicolor*	760	13	*SbOPR*
	*Zea mays*	2365	8	*ZmOPR*
Dicots	*Arabidopsis thaliana*	125	6	*AtOPR*
	*Populus trichocarpa*	485	7	*PtOPR*
	*Medicago truncatula*	500	7	*MtOPR*
Total			74	

* The genome size of each selected organism is based on GenBank release 167.0.

### Phylogenetic relationships and evolution of the OPR gene family in all plant lineages

**Figure 1:**
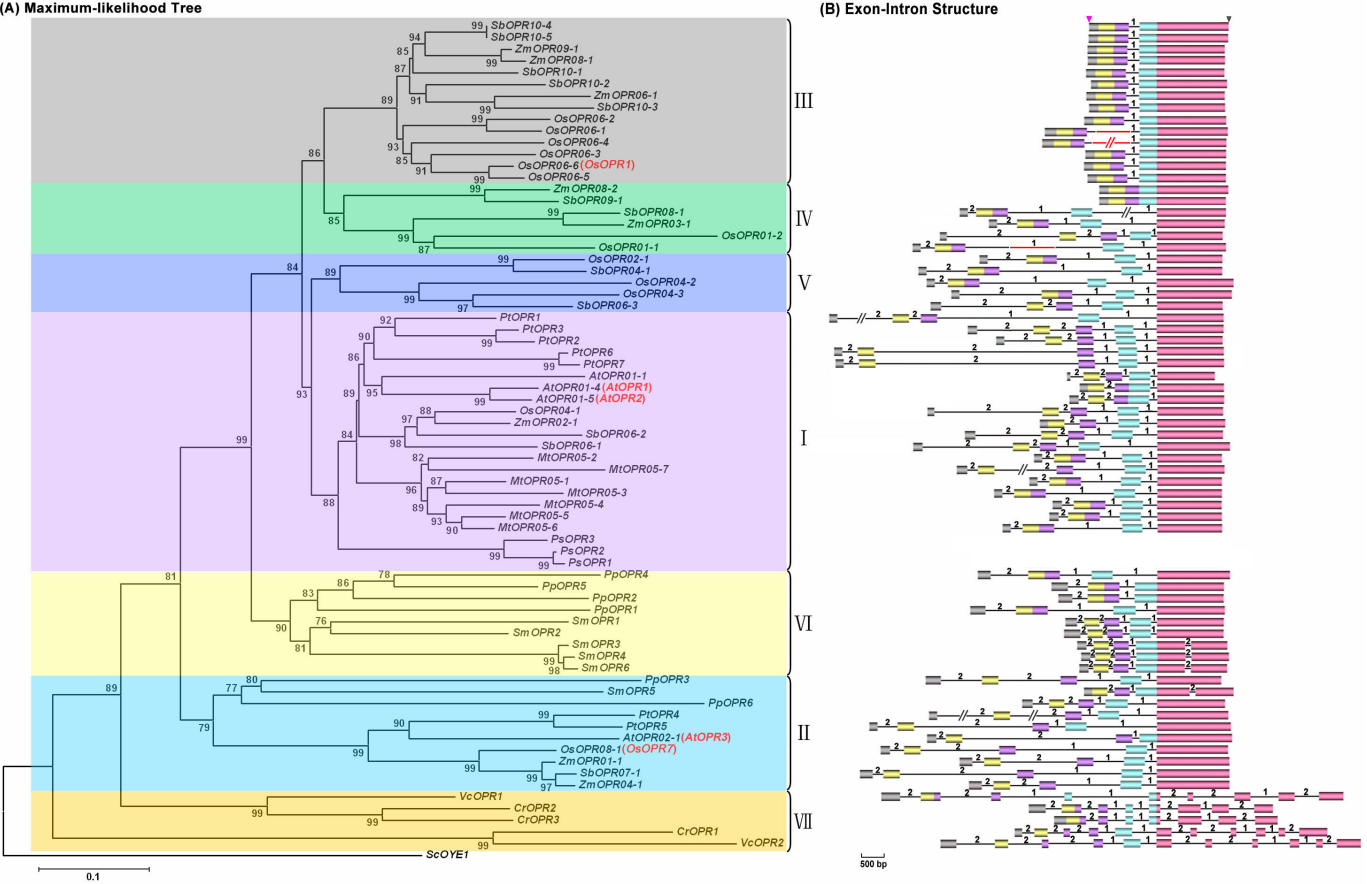
**Phylogenetic relationship and exon-intron structure of OPR genes in 11 representative plants**. (A) The rooted maximum-likelihood (ML) phylogenetic tree was inferred from the amino acid sequences alignment of the Oxidored_FMN domain under the best-fit model WAG+I+G (selected by ProtTest v1.4) with discrete gamma distribution in four categories. All parameters (gamma shape = 1.303; proportion of invariants = 0.042) were estimated from the dataset. The bootstrap values from 1000 resamplings are given at each node and the branch lengths are drawn to scale. (B) Exon-intron structures of the OPR family genes. The untranslated region (UTR) sequences are not shown, and the symbols "pink inverted triangle" and "black inverted triangle" denote the start and stop of codon sequences, respectively. Filled colored boxes: exons; lines: introns; numbers 1 and 2: intron phases. Exons shared with a high degree of identity (≥ 70%) between two OPR genes (Additional files 3, 4) are depicted in the same color. The length of the boxes and lines are scaled based on the length of the genes, except for *PtOPR1*, *PtOPR4*, *MtOPR05-7*, *SbOPR08-1 *and *OsOPR06-4*, for which long introns are denoted by slash-slash. Insertion fragments in the introns of *OsOPR01-1*, *OsOPR06-1 *and *OsOPR06-4*, caused by miniature inverted-repeat transposable elements (MITEs) or retrotransposons, are denoted by bold red lines. The exon-intron structures of OPR genes in the gymnosperm *Picea sitchensis *are not represented here because the genome sequence of *Picea sitchensis *is unavailable and its gene structure is uncertain.

To explore the phylogenetic relationship among OPR paralogues in plants, a rooted maximum-likelihood (ML) phylogenetic tree with 71 OPR genes from 11 species (Figure 1A) was inferred from the amino acid sequences of their Oxidored_FMN domains (Additional file 3, 4), using the PhyML v3.0 program [24] under the best-fit model WAG+I+G. Here, the best-fit model (WAG+I+G) for amino acid substitution was selected by ProtTest v1.4 [25] with discrete gamma distribution in four categories. All parameters (gamma shape = 1.303; proportion of invariants = 0.042) were estimated from the dataset. To compensate for the disadvantages of PhyML in tree-space searches, the ML tree was reconstructed using the Phylip v3.68 package [26] under the gamma-corrected Jones-Taylor-Thornton (JTT) model [27]. The ML trees constructed by PhyML v3.0 and Phylip v3.68 gave congruent topologies (Figure 1A; Additional file 5). Additionally, tree topology assessed by neighbor joining (NJ), minimum evolution (ME) and maximum parsimony (MP) methods (using MEGA v3.1), was substantially similar to the ML tree (data not shown). Using *ScOYE1 *from yeast as the outgroup, the OPR gene family can be subdivided into seven well-conserved subfamilies (Figure 1A) with high statistical support, according to the topology and the deep duplication nodes of OPR paralogues in the ML tree (Figure 1A); we numbered these subfamilies sub. I to sub. VII. All OPR genes from the green algae were grouped into the same subfamily (sub. VII), while those from the land plants were grouped into several other subfamilies (sub. I–VI), showing that the OPR family originated before the divergence of the green algae and the ancestor of land plants. Of the other six subfamilies, only sub. II was present in all land plants except for the gymnosperm *Picea sitchensis*, revealing that all OPR genes from land plants shared a common ancestor after the divergence from aquatic plants. Sub. VI was only present in lower land plants, i.e. mosses and lycophytes, while sub. I, III, IV and V were only present in higher land plants, i.e. gymnosperms and angiosperms. Moreover, sub. III, IV and V were found exclusively in monocots (Figure 1A). These observations indicated that all OPR genes from land plants shared a common ancestor before the divergence between lower and higher land plants; subsequently, lineage-specific expansion and divergence events occurred in higher land plants, especially in monocots, after divergence from lower land plants. In addition, OPR genes from the same lineage, such as mosses, lycophytes, gymnosperms and angiosperms, tended to be clustered together (Figure 1A).

**Figure 2:**
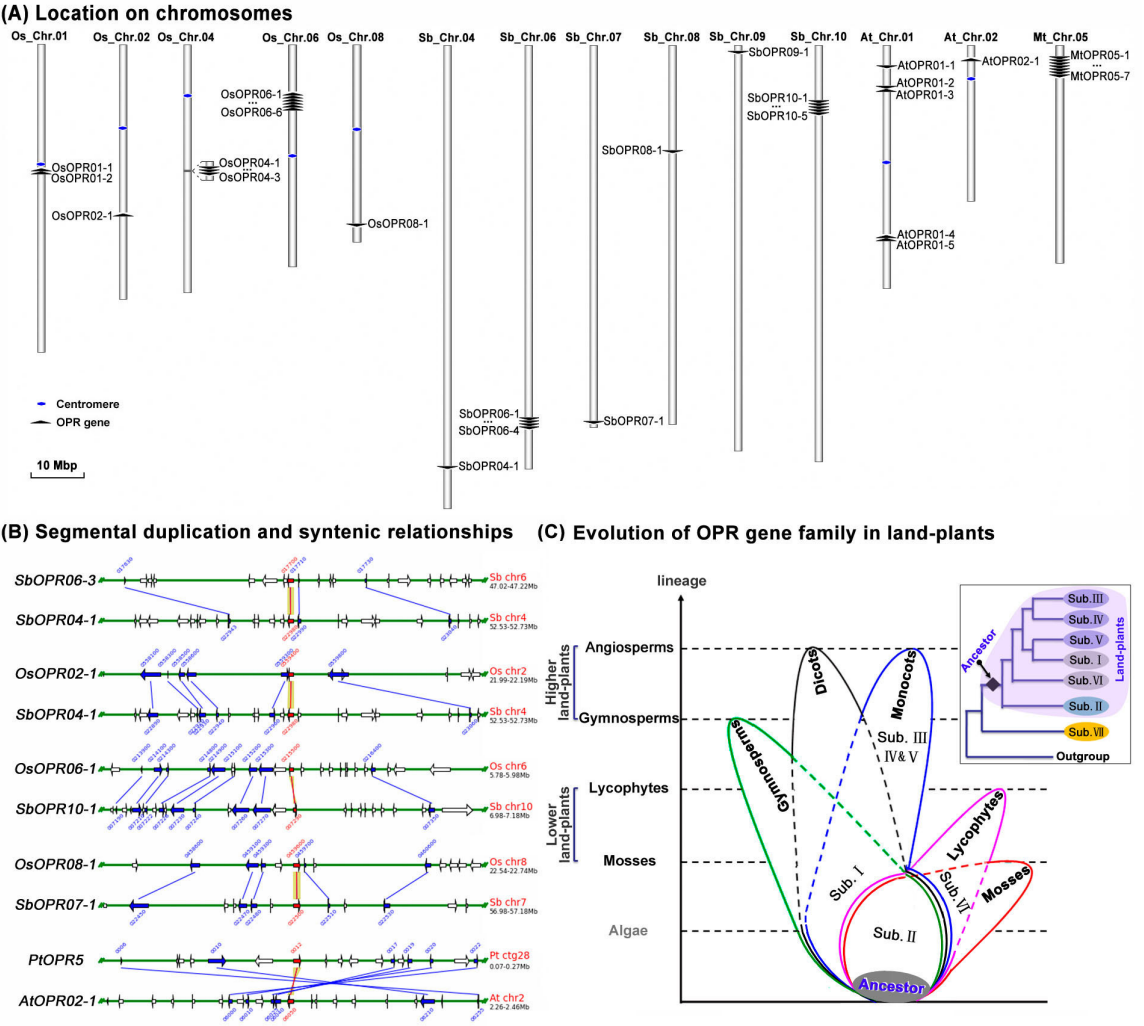
**The expansion and evolution of the OPR gene family in land plants**. (A) Chromosomal location of OPR genes in monocots (*Oryza sativa *and *Sorghum bicolor*) and dicots (*Arabidopsis thaliana *and *Medicago truncatula*). (B) Gene duplications and syntenic relationships of OPR genes in higher land plants. Paralogous gene pairs generated by gene duplications and gene pairs based on cross-genome syntenic relationships within the OPR family of four species (*Arabidopsis thaliana*, *Populus trichocarpa*, *Oryza sativa*, and *Sorghum bicolor*) were analyzed using the PGDD. All intra/cross-species blocks for each query gene display regions of only ~100 kb. Blue arrows indicate the other anchor genes in the region, and red arrows indicate the query locus. Green lines connect gene pairs. (C) A schematic pattern for the expansion and evolution of the OPR gene family in land plants. The phylogenetic relationship of seven OPR subfamilies is represented in the upper right, while the expansion and evolution relationship of six OPR subfamilies (shaded, Sub. I–VI) in land plants is showed in the bottom left. The areas circled in different colors represent different plant lineages: red = mosses, pink = lycophytes, green = gymnosperms, blue = monocots, black = dicots. The "Sub. I" legend in the common area shared by gymnosperms, monocots and dicots indicates that this subfamily exists in all three lineages. Similarly, subfamily II is shared by all lineages, and subfamily VI is shared by lycophytes and mosses; subfamilies III, IV and V, however, only exist in monocots. The "black upright diamond" symbol in the upper diagram and the shaded circle in the lower diagram both represent the common ancestor of the OPR gene family in land plants.

Additionally, the chromosomal location of the OPR genes in the genomes of the monocots (*Oryza sativa *and *Sorghum bicolor*) and dicots (*Arabidopsis thaliana *and *Medicago truncatula*) showed that OPR genes are distributed in clusters (Figure 2A). Moreover, searching for such paralogues within the OPR family of genes using the Plant Genome Duplication Database (PGDD; ) revealed that only one paralogous gene pair (*SbOPR04-1*/*SbOPR06-3*) exists in *Sorghum bicolor *(Figure 2B), but not in other species. Further analysis using the PGDD revealed cross-genome syntenic relationships in four gene pairs: *OsOPR02-1/SbOPR04-1*, *OsOPR06-1/SbOPR10-1*, *OsOPR08-1/SbOPR07-1 *and *PtOPR5/AtOPR02-1 *(Figure 2B). These findings suggest that the ancestral OPR of each subfamily in monocots and dicots underwent tandem duplication, which caused differences in the number of OPR genes within each subfamily and species, while segmental duplication occurred only in the expansion of the OPR family in *Sorghum bicolor*.

Interestingly, phylogenetic analysis showed distinct differences between aquatic and land plants, not only in the number of OPR genes, but also in the number of subfamilies (Figure 1A). Therefore, based on the results obtained from phylogenetic analysis, we proposed a schematic pattern to account for the expansion and evolution of the OPR gene family in plants (Figure 2C). In this pattern, the OPR genes were plant-specific and originated before the divergence of green algae from land plants. The ancestral OPR gene evolved into the present sub. VII and the common ancestral OPR of land plants evolved after the divergence of aquatic plants (green algae) from land plants. Subsequently, the common ancestral OPR of land plants underwent one duplication and yielded two copies: one copy evolved into the present sub. II, while the other copy evolved into the present sub. VI in lower land plants (mosses and lycophytes) and sub. I in higher land plants (gymnosperms and angiosperms) (Figure 2C). The events leading to lineage-specific expansion, especially by tandem duplication, occurred in monocots after their divergence from dicots, and sub. III, IV and V were generated exclusively in monocots (Figure 2C).

### Structural evolution of the OPR family genes

**Figure 3:**
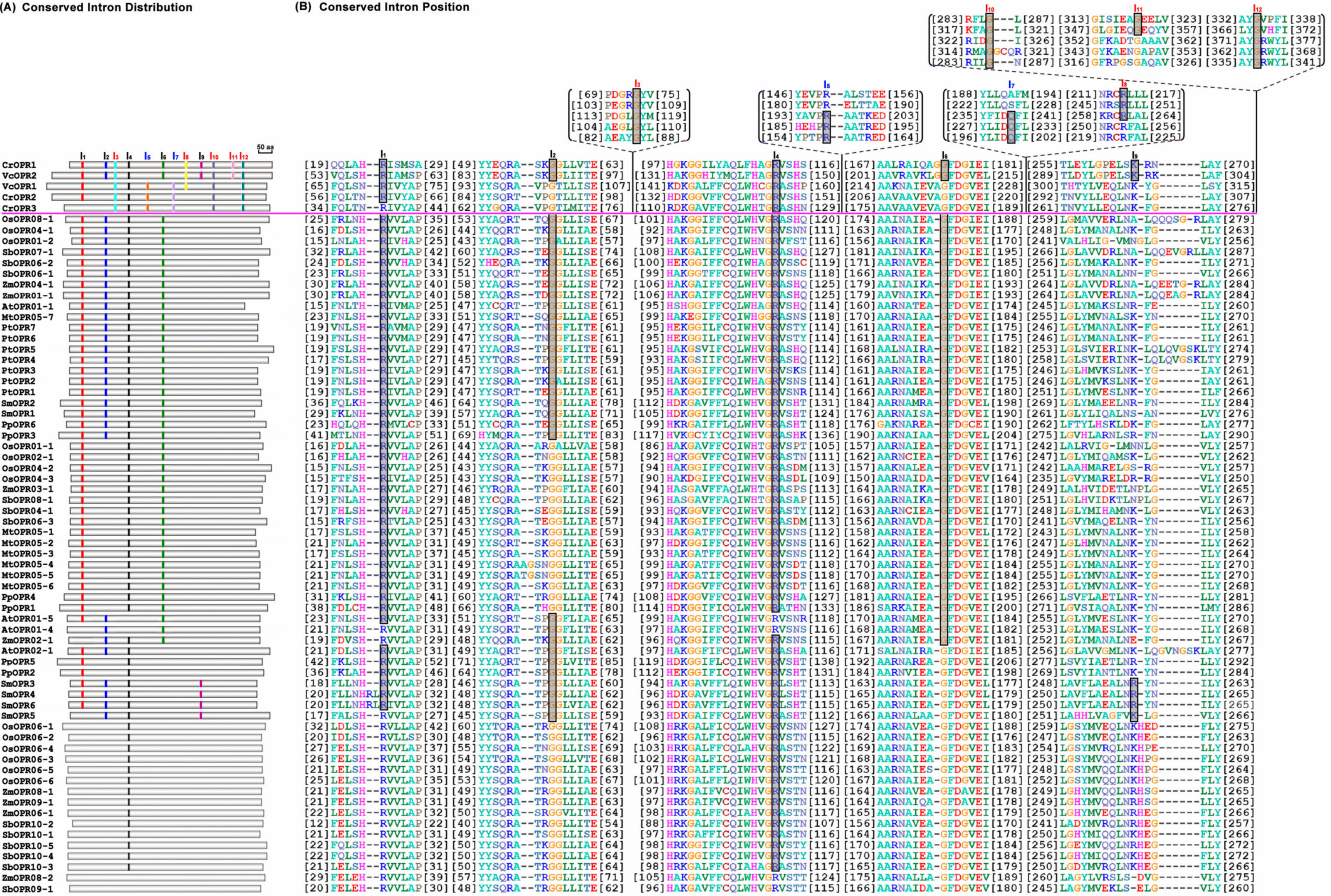
**Distribution and position of conserved introns within the Oxidored_FMN domain**. (A) Schematic comparison of conserved intron distribution of OPR genes. The positions of introns in the coding sequence of the OPR genes are indicated as colored vertical lines and labeled I_1 _to I_12_. Introns 3, 8, 10, 11 and 12 (in red) and introns 5 and 7 (in blue) only exist in aquatic plants (green algae). Moreover, introns 5 and 7 most probably arose in aquatic plants. Introns 1, 2, 4, 6 and 9, labeled in black, are present in both aquatic and land plants. (B) Conserved intron positions in OPR proteins with a topology identical to that in (A). The boundary residues between two exons are denoted by a black translucent box.

To examine the possible mechanisms of structural evolution of OPR paralogues, we compared the exon/intron structures of individual OPR genes in all plant lineages, except for gymnosperms (for which complete or nearly complete genome sequences are unavailable). The exon/intron structures were obtained using the online Gene Structure Display Server (GSDS: ) with either GenBank accession numbers, or both coding sequences (CDS) and genomic sequences [28]. Figure 1B provides a detailed illustration of the relative length of introns and conservation of the corresponding exon sequences within each of the OPR paralogues in plants. Notably, although the members of the OPR gene family exhibited differences in intron number and intron length, the intron positions and intron phases were remarkably well-conserved, with conserved splicing sites between adjacent exons (Figures 1B, 3; Additional file 3). As for the number of introns, the OPR genes in sub. VII contained 6–10 introns while those in sub. III all contained only one intron. Most of the OPR genes in the other five subfamilies, sub. I, II, IV, V and VI, contained 3–4 introns (Figure 1B). Interestingly, the OPR genes in the oldest subfamily, sub. VII, contained the greatest number of introns while those in the youngest subfamily, sub. III, contained the fewest introns (Figure 1B). These findings, together with the phylogenetic trees, indicate that a significant number of intron loss events occurred during the structural evolution of the OPR gene family from green algae to angiosperms.

In addition, a total of 12 different introns have been found within all the genes of the OPR family across different lineage species, according to intron position (Figure 3). Further analysis the introns of OPR paralogues in sub. VII indicated that I_5 _and I_7 _exist only in *VcOPR1 *and *CrOPR2/3*, but not in *CrOPR1 *and *VcOPR2 *(Figures 1B, 3). However, *CrOPR1 *and *VcOPR2 *arose earlier than *VcOPR1 *and *CrOPR2/3*, according to the topology of the ML tree (Figure 1A). These observations suggest that I_5 _and I_7 _were most likely gained by the ancestor of *VcOPR1 *and *CrOPR2/3 *before the divergence between *Chlamydomonas reinhardtii *and *Volvox carteri*. The above-mentioned events of intron loss and I_5 _and I_7 _gain in OPR paralogues are consistent with previous findings by Lin et al. (2006) [29] and Roy and Penny (2007) [30,31].

**Figure 4:**
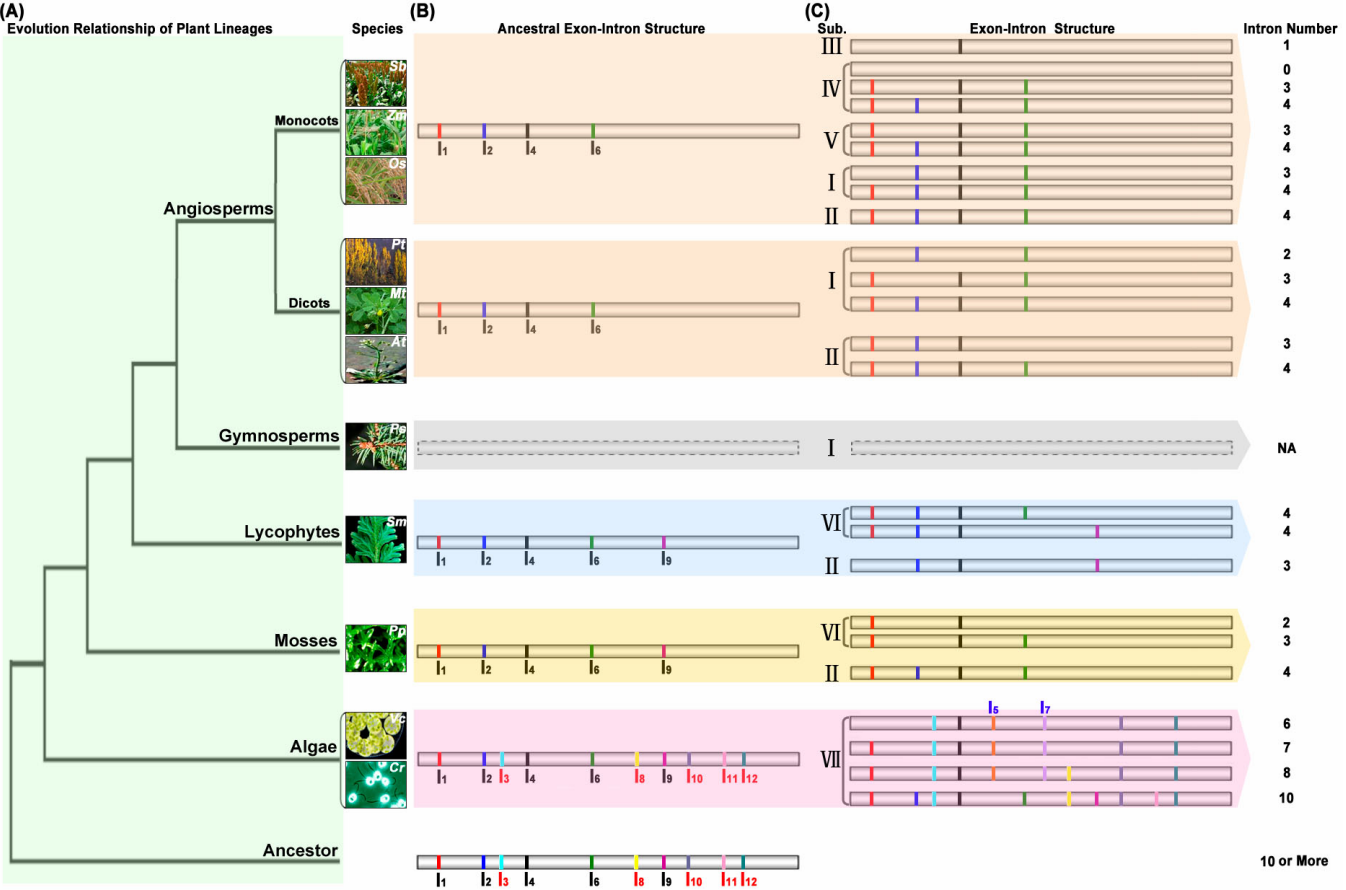
**An evolution model for the structural evolution of the OPR paralogues in plants**. (A) Evolutionary relationship of all plant lineages. (B) Proposed exon-intron structure of the ancestral OPR gene in each plant lineage. I_1_-I_12 _represent introns 1–12; I_1_, I_2_, I_4_, I_6 _and I_9_, labeled in black, are present in both aquatic and land plants; I_3_, I_8_, I_10_, I_11 _and I_12_, labeled in red, only exist in aquatic plants (green algae); I_5 _and I_7_, labeled in blue, most probably arose in aquatic plants (green algae). (C) Current exon-intron structure of OPR genes in all plant lineages. The exon-intron structure of OPR genes in the gymnosperm *Picea sitchensis *is represented with a dashed line, because the genome sequences of *Picea sitchensis *are unavailable and its gene structure is uncertain.

The aforementioned exon/intron structure comparison and the phylogenetic analysis provide strong evidence that single intron loss events occurred during the structural evolution of OPR paralogues from green algae to angiosperms. To further investigate the structural evolution of OPR paralogues in different lineage species, we constructed an evolution model that could yield the current OPR genes in plant species of different lineages (Figure 4). Under the assumption that introns, which were located exactly at the same position and have been given the same phase, should be present in the common ancestor, we reconstructed the ancestral exon/intron structure of OPR for all plant lineages (Figure 4B). The results obtained from the analysis of introns of OPR paralogues in sub. VII suggested that the events of intron gain may have occurred in algae and that I_5 _and I_7 _were most likely gained in *VcOPR1 *and *CrOPR2/3*; therefore, the ancestral exon/intron structure of OPR in algae should contain 10, not 12 introns, and I_5 _& I_7 _were not included (Figure 4B). In this model, the ancestor OPR contained 10 or more introns, symmetrically distributed throughout the coding sequence of OPR, and multiple unique introns were lost during the evolutionary process from green algae to angiosperms (Figure 4B). For example, I_3_, I_8_, I_10_, I_11 _and I_12 _were lost in the evolution from aquatic plants (green algae) to lower land plants (mosses and lycophytes), while I_9 _was lost in the evolution from lower land plants to higher land plants (angiosperms). Moreover, single intron losses also occurred during the expansion and divergence of the OPR gene family in each plant lineage. For example, the ancestral OPR in algae contained at least 10 introns, whereas all five OPR genes contained only 6–10 introns (Figure 4C). This suggests that a single intron loss occurred during the evolution of OPR genes in algae. Similar cases were also found in other lineages, i.e., mosses, lycophytes and angiosperms (dicots and monocots) (Figure 4C). Interestingly, I_1_, I_2_, I_4 _and I_6 _were present in the OPR gene of the common ancestor of all plant lineages (Figure 4B), but some or all of them were lost during the evolution from the ancestral OPR gene to the present individual OPR genes in each plant lineage (Figure 4C). This suggests that these four conserved introns (I_1_, I_2_, I_4 _and I_6_) were retained during the evolution of different plant lineages from algae to angiosperms, but other introns were lost during the structural evolution of OPR paralogues in each plant lineage. In addition to single intron losses, intron gain may have occurred during the structural evolution of OPR paralogues; it appears to have occurred only in green algae (Figures 4B, 4C).

Additionally, the exon-intron structure of OPR genes showed that the length of introns within each individual OPR gene was distinct, with lengths varying from 47 to 2919 bp (data not shown). Further analysis of the introns of OPRs in *Arabidopsis *and rice indicated that the average intron length of *Arabidopsis *OPR genes was 165 bp, close to that of the entire *Arabidopsis *genome (168 bp) calculated from *Arabidopsis *genome TAIR 6.0 release. The average intron length of rice OPR genes was 559 bp, longer than that of the entire rice genome (393 bp) calculated from the TIGR rice genome release 5.0. Moreover, most of these introns are putative miniature inverted-repeat transposable elements (MITEs) or retrotransposons, which can be found in the TIGR Oryza Repeat Database. For example, short fragment insertions in the first intron (213 bp) of *OsOPR01-1 *and the second intron (600 bp) of *OsOPR06-1 *show high homology (> 90%) with the MITEs, while a long fragment insertion in the intron (~5.0 kb) of *OsOPR06-4 *shows high homology (> 90%) with the retrotransposons (Figure 2B). Similar cases were also found in maize and *Sorghum *OPR genes. These results suggest that the presence of indels within introns, caused by MITEs or retrotransposons, may have arisen during the structural evolution of the OPR gene family.

### Variable selective pressures among amino acid sites under diversifying selection

To analyze positive or negative selection of specific amino acid regions within the full-length protein sequences of OPRs, substitution rate ratios of nonsynonymous (dN or Ka) versus synonymous (dS or Ks) mutations (dN/dS or *ω*) were calculated. The Ka/Ks ratio should be 1 for genes subject to neutral selection, < 1 for genes subject to negative selection and > 1 for genes subject to positive selection; however, there are constraints in using Ka/Ks to assess protein evolution for this gene family. Because members of the gene family show few changes in protein sequences, especially for duplicated genes, they may have more similar Ka and Ks values than their parental genes, bringing the Ka/Ks ratio closer to 1 or to less than 1 [32,33]. Amino acids in a protein sequence are expected to be under different selective pressure and to have different underlying dN/dS ratios. In order to test for positive selection at individual amino acid codons, the site-specific models implemented using the codeml program of the PAML v4.0 package [34] were tested. Table 2 lists parameter estimates and log-likelihood values under models of variable *ω *ratios among sites. Model M0 (one ratio) assumes the same ratio for all sites and fits the data much worse than any of the other models, accounting for variable *ω *ratios across sites. For example, the M3 (discrete) model involves four more parameters than M0 (one ratio), and the likelihood rate test (LRT) statistic 2Δℓ = 2439.82 is much greater than the critical value  = 13.28 with df = 4 (Additional file 6). The results suggest that M0 was rejected when compared to M3 (P < 0.01) and the existence of extreme variation in selective pressure among amino acid sites. Moreover, the ratio value (*ω*) in M0 was 1.047, closer to 1 (Table 2), suggesting that the OPR family genes within each subfamily were under strong negative selection pressure and positive selection may have acted in very short regions or on only a few sites during the evolutionary process from algae to angiosperms.

**Table 2:** Likelihood values and parameter estimates for the OPR genes in plants

**Models**	***P***^**a**^	**dN/dS**^**b**^	**Estimates of parameters**^**c**^	ℓ	**Positively Selected Sites**^**d**^
M0 (one-ratio)	1	1.047	*ω *= 1.047	-24650.04	None
M1a (nearly neutral)	2	0.649	p_0 _= 0.415, (p_1 _= 0.585) *ω*_0 _= 0.154, *ω*_1 _= 1	-23834.20	Not allowed
M2a (positive selection)	4	**1.377**	p_0 _= 0.271, p_1 _= 0.447, (p_2 _= 0.283) *ω*_0 _= 0.113, *ω*_1 _= 1, *ω*_2 _= **3.183**	-23430.45	**20**, **22**, **24**, 35*, **39**, 40*, **48**, **51**, 56*, 62*, 69*, 70*, 76, 83*, **102**, **131**, **132**, **135**, **151**, **152**, 157*, 158*, 163*, **170**, **177**, 205*, 207*, 227*, 228*, 233, 243*, **246**, 248*, **249**, 254*, 258*, **262**, **267**, 268, **301**, **302**, **303**, **304**, **305**, 307*, **311**, 314*, **319**, 324*, 327*, 330*, **333**, **334**, **336**, **345**, **354**, **357**, 358, **359**, 361
M3 (discrete)	5	**1.414**	p_0 _= 0.274, p_1 _= 0.449, (p_2 _= 0.227) *ω *= 0.119, *ω*_1 _= 1.049, *ω*_2 _= **3.290**	-23430.13	16, **20**, **22**, **24**, 35*, **39**, 40*, 41*, **48**, **51**, 56*, 62*, 66, 69*, 70*, 76, 83*, 98, **102**, **131**, **132**, **135**, **151**, **152**, 157*, 158*, 159, 163*,**170**, **177**, 205*, 207*, 223, 224, 227*, 228*, 233, 243*, 245, **246**, 248*, **249**, 254*, 258*, **262**, **267**, 268, **301**, **302**, **303**, **304**, **305**, 307*, **311**, 314*, **319**, 324*, 325, 327*, 330*, **333**, **334**, **336**, **345**, **354**, **357**, 358, **359**, 361
M7 (beta)	2	0.593	p = 0.384, q = 0.264	-23749.95	Not allowed
M8 (beta &*ω*)	4	**1.194**	p_0 _= 0.733, (p_1 _= 0.267) p = 0.378, q = 0.264, *ω *= **2.853**	-23413.00	**20**, **22**, **24**, 35*, **39**, 40*, **48**, **51**, 56*, 62*, 69*,70*, 76, 83*, **102**, **131**, **132**, **135**, 151, **152**, 157*, 158*, 163*, **170**, **177**, 205*, 207*, 227*, 228*, 243*, **246**, 248*, **249**, 254*, **262**, **267**, 268, **301**, **302**, **303**, **304**, **305**, 307*, **311**, 314*, **319**, 324*, 327*, 330*, **333**, **334**, **336**, **345**, **354**, **357**, 358, **359**, 361

^a ^Number of parameters in the *ω *distribution.

^b ^The dN/dS ratio is an average over all sites of OPR gene alignments.

^c ^Parameters in parentheses are not free parameters.

^d ^Numbering of amino acid residues corresponds to AtOPR02-1(AtOPR3). Positive-selection sites are inferred at posterior probabilities >95% with those reaching 99% shown in bold. The lists of sites are identical between Naive Empirical Bayes (NEB) and Bayes Empirical Bayes (BEB) analysis in M2a and M8, while only Naive Empirical Bayes (NEB) analysis was used in M3.

* The amino acid residues depicted with an asterisk were also found to be implicated in the functional divergence between OPR paralogues (see Table 3).

All three models that allow for the presence of positive-selection sites, i.e., M2a (positive selection), M3 (discrete) and M8 (beta &*ω*), do suggest the presence of such sites (Table 2). Allowing for the presence of positive-selection sites (with *ω *> 1) significantly improves the fit of the models. The comparison of models M1a and M2a should be stated as a test of the null hypothesis that all genes evolved under neutral conditions, versus the alternate hypothesis that some sites are under negative selection (*ω *< 1), some sites under neutral constraints (*ω *= 1) and some sites under positive selection (*ω *> 1). The neutral model (M1a) does not allow for sites with *ω *> 1, while the positive selection model (M2a) adds an additional site class, with the *ω *ratio estimated to be 3.183. The log-likelihood improvement was huge, as 2Δℓ = 807.50 should be compared with  = 9.21 with df = 2 (Additional file 6). Comparison between M7 (beta) and M8 (beta and *ω*) produced similar results (Additional file 6). This could be explained by the fact that the majority of the protein was subjected to constant negative selection while a few sites underwent positive selection [35].

**Figure 5:**
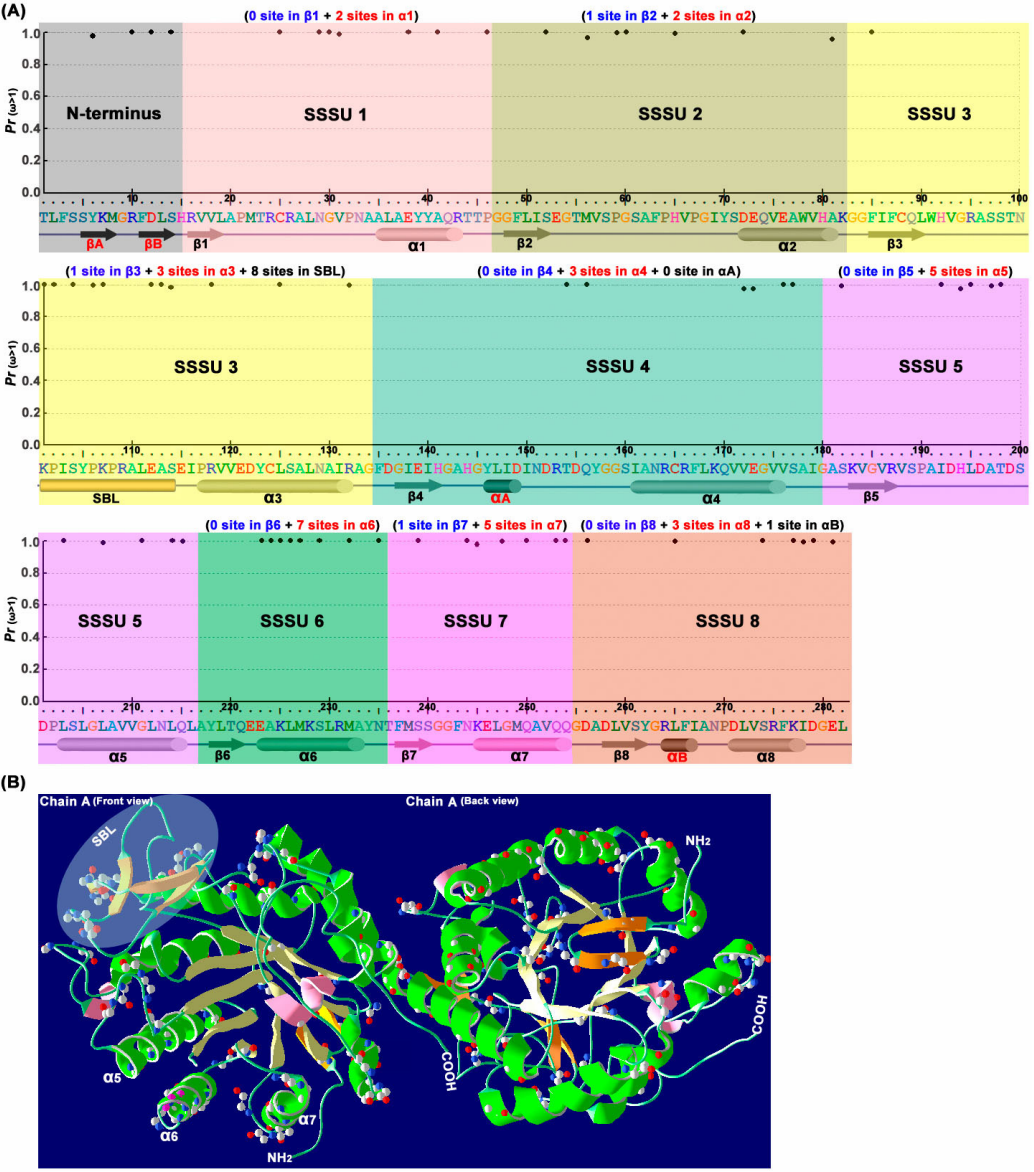
**Distribution of positive-selection sites in the Oxidored_FMN domain**. (A) Posterior probabilities for site classes with positive-selection pressures (measured by the *ω *ratio) for amino acid sites along the sequence. The OPR sequence of the Oxidored_FMN domain is shown below the x-axis along with its secondary structure elements corresponding to the structure of AtOPR1/3 [64,65] and LeOPR3 [66]. Eight *α*/*β*-barrel domains are represented as colored tubes and arrows, respectively, and each *α*/*β*-barrel is defined as one super secondary structure unit (SSSU). Two *β*-sheets that form a short hairpin loop at the N-terminus are represented as black arrows. Helix *α*A, located in SSSU_4_, and helix *α*B, part of SSSU_8_, contribute to a common phosphate-binding motif. And the substrate binding loop (SBL), part of SSSU_3_, forms the ceiling of the substrate-binding pocket. M3 (discrete) is applied to the data in Table 2. (B) OPR crystal structure from AtOPR3 (PDB ID 1Q45). The crystal structure of AtOPR3, with the backbone shown as a ribbon, was obtained using the Swiss-PdbViewer v4.0 [67]; only chain A is shown, in different orientations: front view (Left) and back view (Right). The 8 *α*-helices and *β*-strands in (A) are showed in green and light yellow respectively. Helices *α*A and *α*B are shown in pink, and other strands in the N-terminus and SBL are shown in orange. The substrate binding loop (SBL), which contains four *β*-strands in AtOPR3, is shown in the shaded circle. The positive-selection sites are shown using the as ball-and-stick model, based on the discrete (M3) model (see Table 2).

Additionally, posterior probabilities for site classes were calculated under three models that allow for selection to be tested (M2a, M3 and M8), and the results (data not shown) were similar. For example, the probabilities that site 324 belongs to the class of positive-selection sites (with the *ω *ratio being 3.183 under M2, 3.290 under M3 and 2.853 under M8; Table 2) were 1.000, 0.971, and 0.999 under the three models, respectively. Table 2 lists sites inferred to be under positive selection under different models at the 95% confidence level. Under models M2a, M3 and M8, 60, 69, and 58 sites were detected, respectively, and the majority of positive-selection sites were conserved with all three models. The detailed distribution of positive-selection sites predicted by model M3 is showed in Figure 5. The 69 sites were scattered over the 8 SSSUs (Super Secondary Structure Units) of the Oxidored_FMN domain, except for four sites (16, 20, 22 and 24) at the N-terminus (Figure 5A). Further analysis indicated that 30 out of 69 sites were distributed in *α*-helices *α*1-*α*8, whereas only 3 sites were distributed in *β*-strands, *β*2, *β*3 and *β*7 (Figure 5A). Moreover, nearly 60% of the positive-selection sites (19 out of 30) in *α*-helices were clustered in *α*5 (5 sites), *α*6 (7 sites) and *α*7 (5 sites) (Figure 5A). Interestingly, all of the positive-selection sites in *α*-helices were clustered on the outside of the OPR protein and near the 8 inner *β*-strands (Figure 5B). In addition, 8 of the positive-selection sites were also detected in the substrate binding loop (SBL) (Figure 5A), which was clustered at the top of the OPR protein and formed the ceiling of the substrate-binding pocket (Figure 5B). These observations provide evidence that positive-selection sites on *α*-helices, especially the *α*5, *α*6 and *α*7 helices, together with the SBL, contributed to Darwinian selection and evolution in the OPR gene family.

### Analysis of functional divergence

We further investigated whether amino acid substitutions in the highly conserved Oxidored_FMN domain could have caused adaptive functional diversification. Two types of functional divergence (type-I and type-II) between gene clusters of the OPR family were estimated by posterior analysis using the DIVERGE v2.0 program [36], which evaluates the shifted evolutionary rate and altered amino acid properties after gene duplication [37,38]. In this analysis, the 71 OPR proteins, except for AtOPR01-2/3 and SbOPR06-4 (for which we do not have a complete Oxidored_FMN domain), were used and the estimation was based on the multiple amino acid sequence alignments of the Oxidored_FMN domain (Additional file 3) for any two OPR subfamilies. Pairwise comparisons of paralogous OPRs from subfamilies I to VII were carried out and the rate of amino acid evolution at each sequence position was estimated. Our results, as shown in Table 3, indicated that with three exceptions (subfamily pairs II/IV, V/VI and V/VI), the coefficients of type-I functional divergence (*θ*_I_) between OPR subfamilies were statistically significant (p < 0.05; Table 3), with *θ*_I _values varying from 0.114 to 0.437. These observations indicate that there were significantly site-specific altered selective constraints on most members of the OPR family, leading to subfamily-specific functional evolution after diversification. Nonetheless, in contrast to the findings on type-I functional divergence, there was no evidence of type-II functional divergence among OPR subfamilies, suggesting that the relative importance of type-I and type-II functional divergence might be associated with specific functional classes of the protein family [38].

**Table 3:** Analysis of functional divergence between OPR subfamilies in plants

**Comparison**	***θ*_**1 **_± S.E^**a**^**	***P***^**b**^	**Q**_**k**_**>0.70**^**c**^	**Critical amino acid sites**^**d**^
Sub. I vs II	0.220 ± 0.056	<0.01	7	35*, 65, 70*, 110, 258*, 306, 324*
Sub. I vs III	0.280 ± 0.055	<0.01	11	41*, 54, 69*, 80, 104, 243*, 258*, 312, 324*, 330*, 360
Sub. I vs IV	0.176 ± 0.070	<0.01	1	212
Sub. I vs V	0.351 ± 0.083	<0.01	7	62*, 98, 166, 212, 227*, 254*, 264
Sub. I vs VI	0.229 ± 0.063	<0.01	5	47, 110, 157*, 212, 307*
Sub. I vs VII	0.377 ± 0.077	<0.01	16	38, 43, 56*, 62*, 68, 85, 163*, 178, 207*, 227*, 245, 257,258*, 327*, 331, 332
Sub. II vs III	0.340 ± 0.064	<0.01	14	41*, 65, 69*, 70*, 80, 85, 167, 169, 194, 201, 243*, 306, 308, 309
Sub. II vs IV	0.001 ± 0.022	**>0.05**	0	Not found
Sub. II vs V	0.142 ± 0.133	**>0.05**	0	Not found
Sub. II vs VI	0.269 ± 0.064	<0.01	10	38, 65, 157*, 191, 223, 245, 248*, 307*, 308, 324*
Sub. II vs VII	0.114 ± 0.083	<0.05	0	Not found
Sub. III vs IV	0.350 ± 0.086	<0.01	8	21, 40*, 83*, 187, 254*, 307*, 308, 309
Sub. III vs V	0.437 ± 0.084	<0.01		16 *62, 66, 70*, 82, 104, 158*, 176, 243*, 254*, 255, 258*, 264, 273, 275, 318, 330*
Sub. III vs VI	0.353 ± 0.068	<0.01	13	21, 70*, 87, 158*, 243*, 248*, 255, 259, 309, 312, 314*, 318, 324*
Sub. III vs VII	0.378 ± 0.081	<0.01	12	56*, 62*, 68, 69*, 85, 189, 205*, 228*, 233, 245, 324*, 330*
Sub. IV vs V	0.288 ± 0.155	<0.05	0	Not found
Sub. IV vs VI	0.351 ± 0.083	<0.01	9	47, 157*, 189, 223, 248*, 254*, 265, 307*, 308
Sub. IV vs VII	0.259 ± 0.153	<0.05	0	Not found
Sub. V vs VI	0.159 ± 0.105	**>0.05**	0	Not found
Sub. V vs VII	0.316 ± 0.162	<0.05	2	264, 332
Sub. VI vs VII	0.178 ± 0.072	<0.01	1	245

^a ^*θ *is the coefficient of functional divergence; *θ*_1 _± S.E. is the coefficient of type I functional divergence between two clusters and its standard error.

^b ^The significance level (P value) is computed using Fisher's transformation.

^c ^Q_k_, posterior probability. A site-specific profile based on the posterior probability (Q_k_) was used to identify critical amino acid residues that were responsible for functional divergence.

^d ^Numbering of amino acid residues corresponds to AtOPR02-1. Critical amino acid sites with the highest posterior values (Q_k_>0.70) are shown.

* The amino acid residues depicted with an asterisk were also found to be predicted in positive-selection sites between OPR paralogues (see Table 2).

Moreover, some critical amino acid residues responsible for the functional divergence were predicted based on site-specific profiles in combination with suitable cut-off values derived from the posterior probability of each comparison. In order to reduce false positives, Q_k _> 0.7 (Q_k_, posterior probability) was used as the cutoff to identify type-I functional divergence-related residues in all comparisons between the seven OPR subfamilies; the results are shown in Table 3. These results show distinct differences in the number and distribution of predicted sites for functional divergence within each pair (Table 3; Additional file 7). For example, 5 critical amino acid sites were predicted for the subfamilyI/VI pair and distributed in SSSU (Super Secondary Structure Unit) 1, 3, 4 and 6, while 16 critical amino acids sites were predicted for the subfamilyI/VII pair and distributed in SSSU 1, 2, 3, 4, 5 and 7 (Additional file 7). Further analysis revealed that 2 out of 5 sites in pair I/VI and 9 out of 15 sites in pair I/VII were distributed in *α *helices and only one site (site 62) was located in *β*2 in the subfamily I/VII pair (Additional file 7). Similar cases were found in other subgroup pairs (Additional file 7). In addition, 24 out of 69 positively selected sites detected under model M3 (discrete) implemented in the codeml program of the PAML v4.0 package were also found to be functionally divergent between OPR paralogues (marked by asterisks in Tables 2 and 3). The shifted evolutionary rate at specific amino acid sites throughout the Oxidored_FMN domain within each pair facilitated the functional divergence of OPR subfamilies during the long period of evolution.

## Discussion

### Origin and evolution of the OPR gene family

The OPR gene family is present in all plant species and generally has multiple genes in each species. In this study, we comprehensively analyzed the phylogeny and evolution of the OPR gene family in all plant lineages, and the results showed that seven well-conserved OPR subfamilies exist in plants (Figure 1A). All five OPR genes identified from green algae fell into a separate clade (sub. VII) while all OPR genes from land plants were clustered into sub. I–VI (Figure 1A), suggesting that the OPR gene might have originated before the divergence of green algae and the ancestor of land plants. Moreover, the intron positions and the phases of adjacent exons in the Oxidored_FMN domain were conserved in the OPR genes in land plants (Figures 1B, 3; Additional file 3), suggesting that all land plant OPR genes might have originated from a common ancestor. Additionally, phylogenetic analysis suggested that lineage-specific expansion events occurred after the divergence between lower and higher land plants, leading to the generation of sub. VI in lower land plants and sub. I in higher land plants (Figure 2C). Similarly, lineage-specific expansion events also occurred in higher land plants (monocots), and sub. III, IV and V were generated after the divergence from dicots (Figure 2C).

Gene duplication, including tandem duplication, segmental duplication and genome duplication, continues to be a pervasive process and contributes to biological novelty in evolution [39,40]. In this paper, the clustering distribution of OPR genes (Figure 2A) revealed that tandem duplication had an additional role in determining the current size of the OPR gene family. Moreover, the search for paralogues indicated that the *SbOPR04-1*/*SbOPR06-3 *pair was generated by segmental duplication (Figure 2B); the age estimation of *OsOPR *genes (data not shown) indicates that the divergence time within OPR gene pairs was between 20.5 and 36.9 million years ago (Mya), falling into the period of large-scale duplication events 30–40 Mya [41-43]. These observations suggest that large-scale duplication may also have been involved in the expansion of the OPR gene family in *Sorghum *and rice.

The above analysis reveals that the OPR gene family originated from a common ancestor of green plants, followed by lineage-specific expansion and divergence in each lineage and species during their evolution. Moreover, lineage-specific expansion, especially by tandem duplication, is likely to have contributed to the size of the OPR family and yielded multiple OPR subfamilies in land plants, especially in higher land plants such as monocots. Additionally, large-scale or segmental duplication may have been involved in the expansion of the OPR gene family in *Sorghum *and rice.

### Successive intron loss for structural evolution

Gene duplication is a common phenomenon in plant genomes and continues to be a pervasive force in genome evolution [44]. To date, several models for the evolution of genomes have been proposed based on comparative genome studies of model organisms [45-47], but little attention has been focused on the structural evolution of duplicated gene families. In fact, the structural diversity of gene family members is also a mechanism for the evolution of multiple gene families, and intron loss or gain can be an important step in generating structural diversity and complexity [48]. In this study, we analyzed the structural diversity of OPR genes (Figure 1B) and found that single intron loss events occurred during the expansion and structural evolution of OPR paralogues. We found that most OPR family genes lost two or more introns, and the number and position of intron loss was distinctly different among OPR genes (Figure 4). Furthermore, the intron loss events occurred not only in different plant lineages from algae to angiosperms (Figure 4B), but also in each individual plant lineage, from the ancestral OPR of each individual plant lineage to the present individual OPR genes (Figure 4C). These results, in combination with the phylogenetic trees of the OPR gene family (Figure 1A), suggest that intron losses occurred successively rather than simultaneously. In addition to intron loss, intron gain may have occurred during the structural evolution of OPR paralogues in green algae (Figure 4).

Intron positions have been shown to be remarkably well-conserved over long evolutionary time intervals [49,50], and mounting evidence suggests that lineage-specific intron loss may occur during the evolution of a gene family [51]. In this paper, we observed that the intron positions and intron phases of the OPR family genes were well-conserved (Figures 1B, 3), and some introns (I_1_, I_2_, I_4_, I_6_) were conserved in all plant lineages (Figure 4B). This suggests that lineage-specific intron loss events might have occurred during the expansion and structural evolution of OPR genes and generated diversity of gene structure.

The most commonly used model for intron loss is mRNA-mediated intron loss [52,53], but there are also other possibilities such as simple genomic deletion [54] and in-frame intron deletion [55]. Recent studies [56-58] have indicated that introns closer to the 3' end of genes are preferentially lost, leaving the flanking exons to fuse and form a large exon at the 3' end. In this study, the results of exon-intron structure analysis (Figures 1B, 3) revealed that the introns of OPR genes in aquatic plants (green algae) were distributed relatively symmetrically among the coding sequences, while the introns of OPR genes in land plants were distributed asymmetrically, clustered at the 5' ends with the largest exon at the 3' end (Figures 1B, 3A, 4). Moreover, introns I_8 _to I_12 _(except for I_9_), located on the 3' end, were lost in land plants after the divergence from aquatic plants (Figure 4B). These findings suggest that multiple unique introns, i.e., I_8 _-I_12_, were lost during the evolution from aquatic plants to land plants and that mRNA-mediated intron loss was responsible for their deletion. In contrast, the presence of indels within introns would predict that other mechanisms for intron loss (e.g. simple genomic deletion and in-frame intron deletion) were involved.

### Functional divergence in the OPR gene family

Functional innovations including pseudogene formation [59,60], subfunctionalization [61], neofunctionalization [62] and subneofunctionalization [63] after gene duplication may result in altered functional constraints between the gene clusters of a gene family. In this study, the differences between exon/intron structures and the divergences in amino acid sequences among different subfamilies provided us with some hints that the OPR paralogues may have a variety of physiological functions. The results of the functional divergence analysis (Table 3) suggested that OPR genes should be significantly functionally divergent from each other, owing to the evolutionary rate differences at some amino acid sites. A reasonable explanation for these differences would be that due to amino acid mutations, the OPR family genes evolved some new subgroup-specific functions after divergence. Hence, functional divergence might reflect the existence of long-term selective pressure. Previous studies regarding the enzymatic activity of OPR in *Arabidopsis *and tomato showed that OPR enzymes could be classified into two groups (OPR I and II) based on their substrate specificity, which was determined by the substrate binding loop (SBL) [18,19,64,65]. Site-specific profile analysis of OPR members showed that 24 out of the 69 positive-selection sites found under model M3 (discrete) were functionally divergent and only 2 out of 24 critical amino acid sites (E157 and A158) were located in the SBL (Figure 5; Tables 2, 3 and Additional file 7). Moreover, the majority of positive-selection sites or critical amino acid sites were distributed in *α*-helices, especially in *α*5, *α*6 and *α*7 (Figure 5; Additional file 7). These observations suggest that positive-selection pressure on the SBL, as well as *α*-helices, accelerated the functional divergence and formed multiple subfamilies in plants. Additionally, few positive-selection sites were distributed in *β*-strands (*β*1-*β*8), suggesting that the function of the 8 *β*-strands, clustered inside of the OPR protein, might be to define the conserved fold common to all OPRs and maintain the proteins' structural and/or conformational stability.

Studies on *AtOPRs *showed the *AtOPR01-4 *and *AtOPR01-5*, which belong to subfamily I, preferentially catalyze 9R,13R-OPDA and are predominantly expressed in roots [20], whereas *AtOPR02-1*, which belongs to subfamily II, catalyzes the reduction of 9S,13S-OPDA to form OPC 8:0 and is expressed in flowers and anthers [21,22]. Studies of *Atopr3 *have shown that *opr3 *plants are deficient in the biosynthesis of jasmonic acid and male-sterile, whereas *opr1 *and *opr2 *plants are normal [19,21,22]. These studies, together with the results of the phylogenetic analysis of OPR paralogues in plants, suggest that *OsOPR08-1*, *ZmOPR01-1 *and *ZmOPR04-1*, which belong to subfamily II, are probable candidates for involvement in the JA biosynthesis pathway, while *OsOPR04-1 *and *ZmOPR02-1*, which belong to subfamily I, are likely part of a defense signaling pathway.

Additionally, the expression of *OsOPR06-6 *(*OsOPR1*), a member of subfamily III, in the leaves of two week-old seedlings is induced not only by hormones (JA, MeJA, SA) and environmental stress factors such as drought, salt, chilling, UV and O_3_, but also by protein phosphatase inhibitors such as cantharidin (CN), endothall (EN) and okadaic acid (OA) [16], while its expression in suspension-cultured rice cells is induced by JA and protein synthesis inhibitor cycloheximide (CHX) [15]. The results of our phylogenetic analysis of the OPR gene family, together with the previous studies, suggest that subfamily III, existing exclusively in monocots, may have an important role in defense signaling pathways and the mitogen-activated protein kinase (MAPK) pathway. The other two subfamilies (IV and V) may represent pseudogene, subfunctionalization, or neofunctionalization families of genes. Further experiments need to be performed to elucidate the function of these genes in monocots.

## Conclusion

This study provides a comparative genomic analysis addressing the phylogenetic relationships and evolution of the OPR gene family in eleven species representing six major lineages within the green plants. The results of the phylogenetic analysis revealed that seven well-conserved subfamilies exist in plants and that all OPR paralogues originated from a common ancestor of green plants. Lineage-specific expansion, primarily through tandem duplication, contributed to the size of the OPR gene family, and multiple subfamilies formed in land plants after divergence from aquatic plants. The exon/intron structure analysis showed that the gene structures were diverse, while the intron positions and intron phases were highly conserved across different lineages. These observations together with the results obtained from the phylogenetic analysis indicate that successive single intron losses, as well as indels within introns, were involved in the structural evolution of OPR paralogues. Finally, the functional divergence analysis between OPR paralogues suggested that significantly site-specific altered selective constraints acted on most OPR paralogues after gene duplication, leading to subgroup-specific functional evolution after their phylogenetic diversification. This study also demonstrates that amino acids critical for functional divergence are located in the regions including the substrate binding loop (SBL), as well as in *α*-helices (especially helices *α*5, *α*6 and *α*7), indicating the importance of these regions in OPR proteins. These data may provide valuable information for future studies of the function of this gene family, especially subfamilies III, IV and V in monocots.

## Methods

### Identification of OPR genes and their homologues in plants

To identify OPR genes and their homologues in plants, the BLASTP and TBLASTN programs were used to search the TIGR (The Institute for Genomic Research, ), TAIR (The Arabidopsis Information Resource, ), MaizeGDB (Maize Genetics and Genomics Database, ), PlantGDB (Plant Genome Database, ), JGI (Joint Genome Institute, ) and NCBI (The National Center for Biotechnology Information, ) non-redundant databases for protein sequences of the three previously reported OPRs in *Arabidopsis *[10,20]. The Blast searches were performed with the following criteria: E value < 1 × e-05 and only OPR or OPR-like genes from plants were included. Moreover, proteins identified by the BLAST search algorithms were considered as potential homologues when amino acid identity was above 25% over a stretch of 200 amino acids. Then, the Pfam  and SMART  databases were employed to detect conserved domains with OPR or OPR-like protein candidates. Finally, based on the Pfam and SMART analysis, we refined the search results manually to further reduce hits with partially conserved functional domains and other false positives.

### Sequence alignment and phylogenetic analysis

Multiple-sequence alignment is the first step in phylogenetic analysis and the alignment quality may have an enormous impact on the final phylogenetic tree [68-70]. Amino acid sequences of OPR genes and their homologues in plants were aligned using the EBI web tool Clustal W v2.0 program [71] with the default parameters. The GBlocks 0.91b program [70,72] was then used to select the conserved blocks of the above alignment with the default parameters underlined. Thus, the poorly aligned positions, gap positions and divergent regions from the alignment were completely excluded from the phylogenetic analyses. The Akaike Information criterion (AIC) was implemented in ProtTest v1.4 [25] to estimate the most appropriate model of amino acid substitution for tree-building analyses. ProtTest v1.4 is based on the PhyML program [73] for maximum likelihood (ML) optimizations, and the best-fit model considers the relative rates of amino acid replacement and the evolutionary constraints imposed by conservation of protein structure and function. Then, according to the best-fit model predicted by ProtTest v1.4, a rooted maximum likelihood tree was constructed from the Gblocks alignment using the PhyML v3.0 online program [24], and the reliability of interior branches was assessed with 1000 bootstrap resamplings. Considering the limitations of PhyML in tree-space searches, the Phylip v3.68 package [26] was used to reconstruct the ML tree under the Jones-Taylor-Thornton (JTT) model [27]. Finally, the phylogenetic trees were displayed using MEGA v3.1 [74]. In addition, another three phylogenetic trees were reconstructed with MEGA v3.1 from the Gblocks alignment, by employing the neighbor joining (NJ), minimal evolution (ME) and maximum parsimony (MP) methods, respectively.

### Estimating the pattern of nucleotide substitution and positive-selection sites

The diversity of OPR genes was examined with molecular evolutionary analyses using *ω*, which is the ratio of nonsynonymous substitutions (dN) to synonymous substitutions (dS), and a simple and useful measurement of protein evolution [33]. Considering that positive selection may act in very short episodes or on only a few sites during the evolution of duplicated genes, we calculated the *ω *ratio for various amino acid sites and detected the positive selection sites (*ω *> 1). First, accurate nucleotide sequences and related multiple protein sequence alignments were obtained with PAL2NAL [75], a program that constructs multiple codon alignments from matching protein sequences. Then, the resulting codon alignments and NJ tree were used in the program codeml from the PAML v4.0 package [34] to calculate the dN/dS (or *ω*) ratio for each site and to test different evolutionary models. The improved versions of site-specific models, recommended by Anisimova et. al. [35] and Wong et. al., [76] were tested: Models M0 (one ratio), M1a (nearly neutral), M2a (positive selection), M3 (discrete), M7 (beta) and M8 (beta+ *ω*) were all used in this analysis. Model M0 assumed a constant *ω *ratio, while in models M1a and M2a *ω *is estimated from the date (0 <*ω*0 < 1) while *ω*1 = 1 is fixed. M7 and M8 assume a *β*-distribution for *ω *between 0 and 1. Models M2a, M3, and M8 allow for the occurrence of positively selected sites (*ω *> 1). Subsequent likelihood rate comparisons of M0 with M3, M1a with M2a, and M7 with M8, respectively, were performed to test which model fits the data better. The difference in log likelihood between the models, multiplied by two, was compared with a chi-square distribution with n degrees of freedom, n being the difference between the numbers of parameters of the two models. A significantly higher likelihood of the alterative model compared to the null model suggests positive selection. Finally, the Naive Empirical Bayes (NEB) and/or Bayes Empirical Bayes (BEB) approach were used to calculate the posterior probability that each site belongs to the site class of positive selection under each model.

### Functional divergence and altered functional constraint analysis

To estimate the level of functional divergence and predict important amino acid residues for these functional differences among OPR subfamilies, the coefficients of type-I and type-II functional divergence (*θ*_I _and *θ*_II_) between any two clusters were calculated for each position in the alignment (Additional file 3), using the method suggested by Gu et. al. (1999, 2006) [38,77], as implemented in the DIVERGE v2.0 package [36]. This method is based on maximum likelihood procedures to estimate significant changes in the site-specific shift of evolutionary rate or site-specific shift of amino acid properties after the emergence of two paralogous sequences. The advantage of this method is that it uses amino acid sequences and, thereby, is not sensitive to saturation of synonymous sites. Type I designates amino acid configurations that are very conserved in gene 1 but highly variable in gene 2, or vice versa, implying that these residues have experienced altered functional constraints (i.e., different evolutionary rates) [77,78]. Type II designates amino acid configurations that are very conserved in both genes but whose biochemical properties are very different (e.g., positive versus negative charge), implying that these residues may be responsible for functional specification [78,79]. *θ*_I _or *θ*_II _values that are significantly greater than 0, suggest site-specific altered selective constraints or a radical shift of amino acid physiochemical properties after gene duplication. Moreover, a site-specific posterior analysis was used to predict amino acid residues that were crucial for functional divergence.

## Authors' contributions

HW and WL designed the study. WL carried out the data mining, sequence alignments and bioinformatics analysis, and wrote the manuscript. HW and JW conceived of and supervised the study, provided funding and critically revised the manuscript. LY, BL and DF provided some advice for the revision of the manuscript. All authors read and approved the final manuscript.

## Supplementary Material

Additional file 1 **OPR family genes in eleven representative plants**. This table lists the 74 OPR genes from the eleven representative plants (i.e. *Chlamydomonas reinhardtii*, *Volvox carteri*, *Physcomitrella patens*, *Selaginella moellendorffii*, *Picea sitchensis*, *Oryza sativa*, *Sorghum bicolor, Zea mays, Arabidopsis thaliana*, *Populus trichocarpa *and *Medicago truncatula*), as summarized in Tables 1. Click here for file

Additional file 2 **OPR family genes in other monocots and dicots plants**. This table lists the 31 OPR genes from the other monocots (i.e. *Hordeum vulgare *and *Setaria italica*) and dicots (i.e. *Vitis vinifera*, *Pisum sativum*, *Solanum lycopersicum*, *Lithospermum erythrorhizon*, *Carica papaya*, *Artemisia annua*, *Catharanthus roseus*, *Hevea brasiliensis *and *Vigna unguiculata*) plants. Click here for file

Additional file 3 **Amino acid sequences alignment of Oxidored_FMN domain**. Amino acid sequences alignment of Oxidored_FMN domain in OPR genes from eleven species representing six major lineages within the green plants: the green algae (*Chlamydomonas reinhardtii *and *Volvox carteri*), the mosses (*Physcomitrella patens*), the lycophytes (*Selaginella moellendorffii*), the gymnosperms (*Picea sitchensis*), the monocotyledoneous angiosperms (*Oryza sativa*, *Sorghum bicolor *and *Zea mays*) and the dicotyledoneous angiosperms (*Arabidopsis thaliana*, *Populus trichocarpa *and *Medicago truncatula*). Only 3 of 74 OPR genes (AtOPR01-2/3 and SbOPR06-4) without complete Oxidored_FMN domain are excluded, while the outgroup ScOYE1 from yeast is included in the alignment. The blue blocks below the alignment represent the fragments selected by the Gblocks program with default parameters underlined. Positions of the alignments where more than 50% of the sequences are identical are shown with black boxes. The secondary structure elements are represented at the bottom of the alignment as tubes or arrows shown in gray based on structural information from OPR proteins AtOPR1, AtOPR3 and LeOPR3. The intron positions of putative ancestral OPR are represented at the top of the alignment as red vertical arrows, and I_1_~I_12 _represent intron 1~12. The seven conserved OPR subfamilies are represented as I, II, III, IV, V, VI and VII at the left side of the alignment. Click here for file

Additional file 4 **Nucleotide sequences alignment of OPR family genes from eleven species**. Nucleotide sequences alignment of OPR genes from eleven species representing the six major lineages within the green plants: the green algae (*Chlamydomonas reinhardtii *and *Volvox carteri*), the mosses (*Physcomitrella patens*), the lycophytes (*Selaginella moellendorffii*), the gymnosperms (*Picea sitchensis*), the monocotyledoneous angiosperms (*Oryza sativa*, *Sorghum bicolor *and *Zea mays*) and the dicotyledoneous angiosperms (*Arabidopsis thaliana*, *Populus trichocarpa *and *Medicago truncatula*). Identical residues in the alignments are shaded and seven well-conserved OPR subfamilies are represented as Sub.I, II, III, IV, V, VI and VII at the left side of the alignment. Click here for file

Additional file 5 **The rooted maximum-likelihood phylogenetic tree of OPR family genes from eleven representative species**. The rooted maximum-likelihood (ML) phylogenetic tree of OPR genes in eleven representative plants was inferred from the amino acid sequence alignments of the Oxidored_FMN domain (Additional file 3) under the Jones-Taylor-Thornton (JTT) model, implemented in Phylip v3.68 package. The old yellow enzyme 1 (OYE1) of *Saccharomyces cerevisiae *(*ScOYE1*) was selected as an out of group marker and its accession number in SWISS-PROT is Q02899. The bootstrap values from 1000 resamplings are given at each node and the branch lengths are drawn to scale. The seven conserved OPR subfamilies are represented as Sub.I, II, III, IV, V, VI and VII at the right side of the ML tree. Click here for file

Additional file 6 **Likelihood ratio statistics (2Δℓ)**. This data provide represent the likelihood ratio statistical analysis of the comparisons of M0 with M3, M1a with M2a, and M7 with M8, respectively. Click here for file

Additional file 7 **The distribution of positive-selection sites and/or critical amino acid residues in Oxidored_FMN domain**. This data provide represent the detailed distribution of positive-selection sites and/or critical amino acid residues in Oxidored_FMN domain, which are responsible for functional divergence between OPR paralogues. Click here for file
