# Supplementary material for: Phylogenetic analysis, structural evolution and functional divergence of the 12-oxo-phytodienoate acid reductase gene family in plants
Source: BMC Evol Biol. 2009 May 5;9:90. doi: 10.1186/1471-2148-9-90 (PMC2688005; doi:10.1186/1471-2148-9-90)
Supplement: Additional file 3 — Amino acid sequences alignment of Oxidored_FMN domain. Amino acid sequences alignment of Oxidored_FMN domain in OPR genes from eleven species representing six major lineages within the green plants: the green algae (Chlamydomonas reinhardtii and Volvox carteri), the mosses (Physcomitrella patens), the lycophytes (Selaginella moellendorffii), the gymnosperms (Picea sitchensis), the monocotyledoneous angiosperms (Oryza sativa, Sorghum bicolor and Zea mays) and the dicotyledoneous angiosperms (Arabidopsis thaliana, Populus trichocarpa and Medicago truncatula). Only 3 of 74 OPR genes (AtOPR01-2/3 and SbOPR06-4) without complete Oxidored_FMN domain are excluded, while the outgroup ScOYE1 from yeast is included in the alignment. The blue blocks below the alignment represent the fragments selected by the Gblocks program with default parameters underlined. Positions of the alignments where more than 50% of the sequences are identical are shown with black boxes. The secondary structure elements are represented at the bottom of the alignment as tubes or arrows shown in gray based on structural information from OPR proteins AtOPR1, AtOPR3 and LeOPR3. The intron positions of putative ancestral OPR are represented at the top of the alignment as red vertical arrows, and I1~I12 represent intron 1~12. The seven conserved OPR subfamilies are represented as I, II, III, IV, V, VI and VII at the left side of the alignment. [file 1471-2148-9-90-S3.pdf]

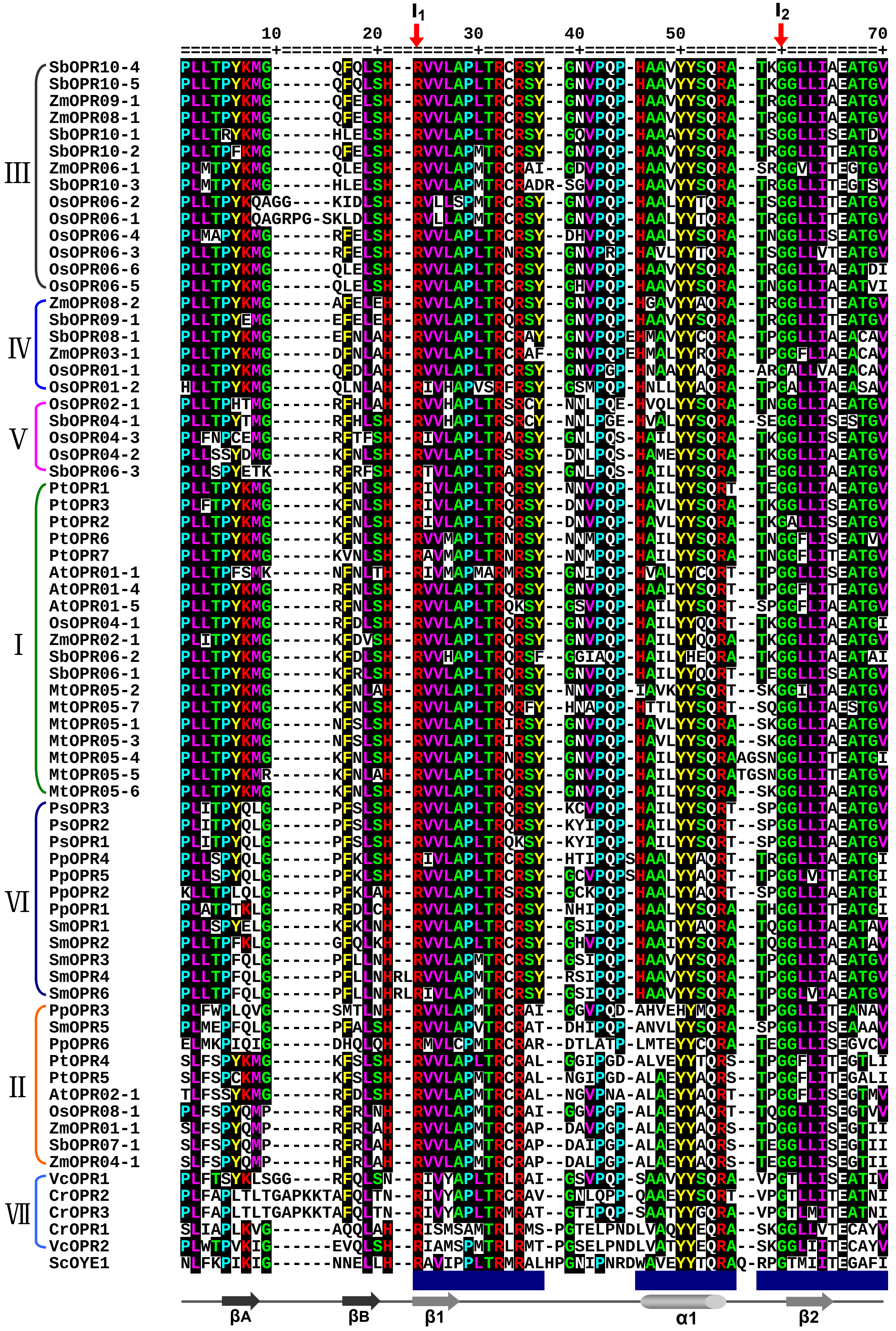
I1
I2
10
20
30
40
50
70
III
SbOPR10-4
SbOPR10-5
ZmOPR09-1
ZmOPR08-1
SbOPR10-1
SbOPR10-2
ZmOPR06-1
SbOPR10-3
OsOPR06-2
OsOPR06-1
OsOPR06-4
OsOPR06-3
OsOPR06-6
OsOPR06-5
IV
ZmOPR08-2
SbOPR09-1
SbOPR08-1
ZmOPR03-1
OsOPR01-1
OsOPR01-2
V
OsOPR02-1
SbOPR04-1
OsOPR04-3
OsOPR04-2
SbOPR06-3
I
PtOPR1
PtOPR3
PtOPR2
PtOPR6
PtOPR7
AtOPR01-1
AtOPR01-4
AtOPR01-5
OsOPR04-1
ZmOPR02-1
SbOPR06-2
SbOPR06-1
MtOPR05-2
MtOPR05-7
MtOPR05-1
MtOPR05-3
MtOPR05-4
MtOPR05-5
MtOPR05-6
VI
PsOPR3
PsOPR2
PsOPR1
PpOPR4
PpOPR5
PpOPR2
PpOPR1
SmOPR1
SmOPR2
SmOPR3
SmOPR4
SmOPR6
II
PpOPR3
SmOPR5
PpOPR6
PtOPR4
PtOPR5
AtOPR02-1
OsOPR08-1
ZmOPR01-1
SbOPR07-1
ZmOPR04-1
VII
VcOPR1
CrOPR2
CrOPR3
CrOPR1
VcOPR2
ScOYE1
βA
βB
β1
α1
β2

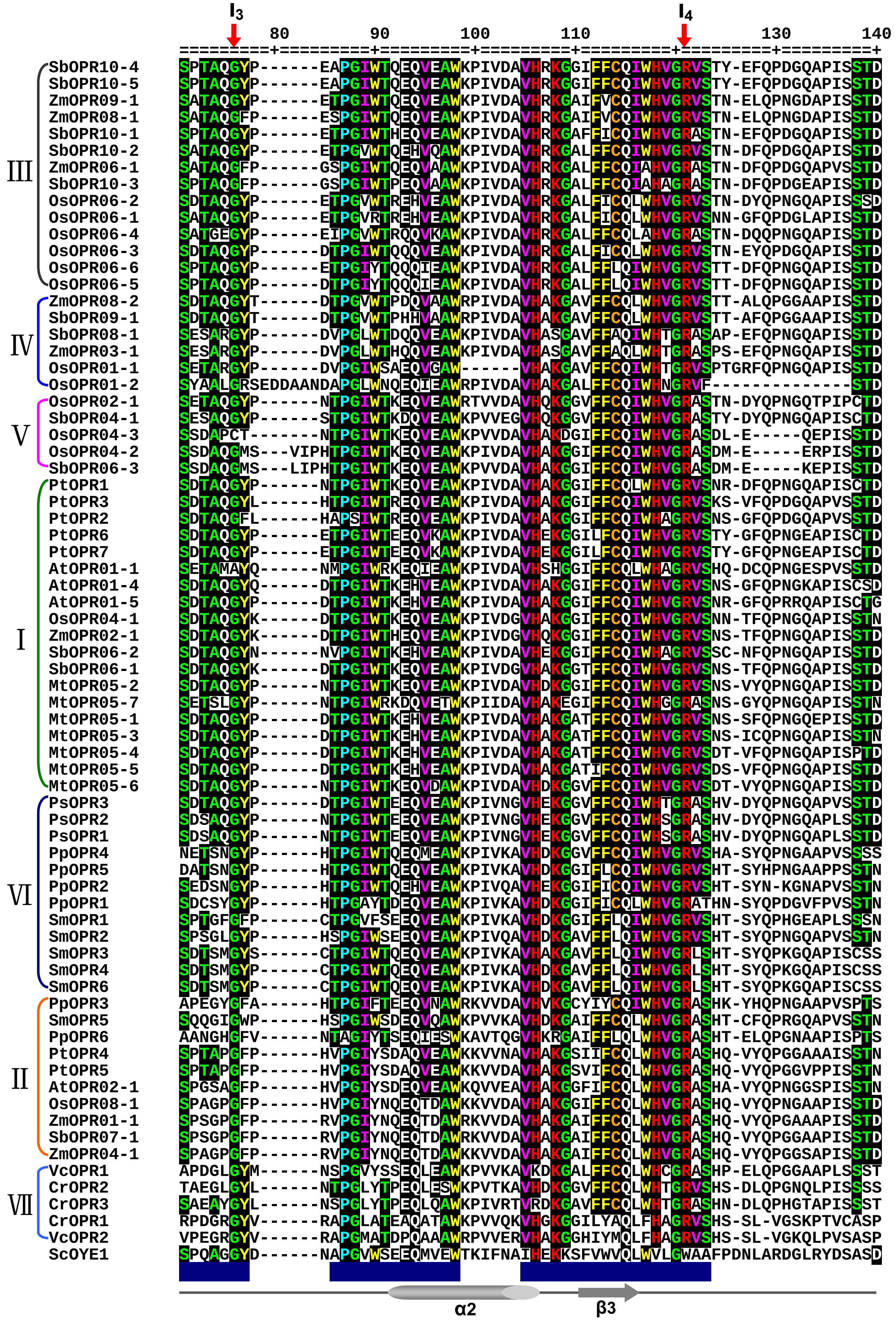
I3
I4
80
90
100
110
130
140
III
IV
V
I
VI
II
VII
SbOPR10-4 SPTAQGYP------EAPGIWTQEQVEAWKPIVDAVHRKGGIFFCQIWHVGRVSTY-EFQPDGQAPISSTD
SbOPR10-5 SPTAQGYP------EAPGIWTQEQVEAWKPIVDAVHRKGGIFFCQIWHVGRVSTY-EFQPDGQAPISSTD
ZmOPR09-1 SATAQGYP------ETPGIWTQEQVEAWKPIVDAVHRKGAIFVCQIWHVGRVSTN-ELQPNGDAPISSTD
ZmOPR08-1 SATAQGFP------ESPGIWTQEQVEAWKPIVDAVHRKGAIFVCQIWHVGRVSTN-ELQPNGDAPISSTD
SbOPR10-1 SPTAQGYP------ETPGIWTHEQVEAWKPIVDAVHRKGAFFICQIWHVGRASTN-EFQPDGQAPISSTD
SbOPR10-2 SATAQGYP------ETPGVWTQEHVQAWKPIVDAVHRKGALFFCQIWHVGRVSTN-DFQPDGQAPISSTD
ZmOPR06-1 SATAQGFP------GSPGIWTQEQVAAWKPIVDAVHRKGALFFCQIAHVGRASTN-DFQPDGQAPVSSTD
SbOPR10-3 SPTAQGFP------GSPGIWTPEQVAAWKPIVDAVHRKGALFFCQIAHAGRASTN-DFQPDGEAPISSTD
OsOPR06-2 SDTAQGYP------ETPGVWTREHVEAWKPIVDAVHRKGALFICQLWHVGRVSTN-DYQPNGQAPISSSD
OsOPR06-1 SATAQGYP------ETPGVRTREHVEAWKPIVDAVHRKGALFICQLWHVGRVSNN-GFQPDGLAPISSTD
OsOPR06-4 SATGEGYP------EIPGVWTRQQVKAWKPIVDAVHRKGALFFCQLAHVGRASTN-DQQPNGQAPISSTD
OsOPR06-3 SDTAQGYP------DTPGIWTQQQVEAWKPIVDAVHRKGALFICQLWHVGRVSTN-EYQPDGQAPISSTD
OsOPR06-6 SPTAQGYP------ETPGIYTQQQIEAWKPIVDAVHRKGALFFLQIWHVGRVSTT-DFQPNGQAPISSTD
OsOPR06-5 SPTAQGYP------DTPGIYTQQQIEAWKPIVDAVHRKGALFFLQIWHVGRVSTT-DFQPNGQAPISSTD
ZmOPR08-2 SDTAQGYT------DTPGVWTPDQVAAWRPIVDAVHAKGAVFFCQLWHVGRVSTT-ALQPGGAAPISSTD
SbOPR09-1 SDTAQGYT------DTPGVWTPHHVAAWRPIVDAVHAKGAVFFCQLWHVGRVSTT-AFQPGGAAPISSTD
SbOPR08-1 SESARGYP------DVPGLWTDQQVEAWKPIVDAVHASGAVFFAQIWHTGRASAP-EFQPNGQAPISSTD
ZmOPR03-1 SESARGYP------DVPGLWTHQQVEAWKPIVDAVHASGAVFFAQLWHTGRASPS-EFQPNGQAPISSTD
OsOPR01-1 SETARGYP------DVPGIWSAEQVGAW------VHAKGAVFFCQIWHTGRVSPTGRFQPNGQAPISSTD
OsOPR01-2 SYAALGRSEDDAANDAPGLWNQEQIEAWRPIVDAVHAKGALFFCQIWHNGRVF-------------STD
OsOPR02-1 SETAQGYP------NTPGIWTKEQVEAWRTVVDAVHQKGGVFFCQIWHVGRASTN-DYQPNGQTPIPCTD
SbOPR04-1 SESAQGYP------STPGIWTKDQVEAWKPVVEGVHQKGGVFFCQIWHVGRASTY-DYQPNGQAPISCTD
OsOPR04-3 SSDAPCT-------NTPGIWTKEQVEAWKPVVDAVHAKDGIFFCQIWHVGRASDL-E-----QEPISSTD
OsOPR04-2 SSDAQGMS---VIPHTPGIWTKEQVEAWKPIVDAVHAKGGIFFCQIWHVGRASDM-E-----ERPISSTD
SbOPR06-3 SSDAQGMS---LIPHTPGIWTKEQVEAWKPVVDAVHAKGGIFFCQIWHVGRASDM-E-----KEPISSTD
PtOPR1 SDTAQGYP------NTPGIWTKEQVEAWKPIVDAVHAKGGIFFCQLWHVGRVSNR-DFQPNGQAPISCTD
PtOPR3 SDTAQGYL------HTPGIWTREQVEAWKPIVDAVHAKGGIFFCQIWHVGRVSKS-VFQPDGQAPVSSTD
PtOPR2 SDTAQGFL------HAPSIWTREQVEAWKPIVDAVHAKGGIFFCQIWHAGRVSNS-GFQPDGQAPVSSTD
PtOPR6 SDTAQGYP------ETPGIWTEEQVKAWKPIVDAVHEKGGILFCQIWHVGRVSTY-GFQPNGEAPISCTD
PtOPR7 SDTAQGYP------ETPGIWTEEQVKAWKPIVDAVHEKGGILFCQIWHVGRVSTY-GFQPNGEAPISCTD
AtOPR01-1 SETAMAYQ------NMPGIWRKEQIEAWKPIVDAVHSHGGIFFCQLWHAGRVSHQ-DCQPNGESPVSSTD
AtOPR01-4 SDTAQGYQ------DTPGIWTKEHVEAWKPIVDAVHAKGGIFFCQIWHVGRVSNS-GFQPNGKAPISCSD
AtOPR01-5 SDTAQGYP------DTPGIWTKEHVEAWKPIVDAVHAKGGIFFCQIWHVGRVSNR-GFQPRRQAPISCTG
OsOPR04-1 SDTAQGYK------DTPGIWTKEQVEAWKPIVDGVHAKGGIFFCQIWHVGRVSNN-TFQPNGQAPISSTN
ZmOPR02-1 SDTAQGYK------DTPGIWTHEQVEAWKPIVDGVHQKGGIFFCQIWHVGRVSNS-TFQPNGQAPISSTD
SbOPR06-2 SDTAQGYN------NVPGIWTKEHVEAWKPIVDAVHEKGGIFFCQIWHAGRVSSC-NFQPNGQAPISSTD
SbOPR06-1 SDTAQGYK------DTPGIWTKEQVEAWKPIVDGVHAKGGTFFCQIWHVGRVSNS-TFQPNGQAPISSTD
MtOPR05-2 SDTAQGYP------NTPGIWTKEQVEAWKPIVDAVHDKGGIFFCQIWHVGRVSNS-VYQPNGQAPISSTD
MtOPR05-7 SETSLGYP------NTPGIWRKDQVETWKPIIDAVHAKEGIFFCQIWHGGRASNS-GYQPNGQAPISSTN
MtOPR05-1 SDTAQGYP------DTPGIWTKEHVEAWKPIVDAVHAKGATFFCQIWHVGRVSNS-SFQPNGQEPISSTD
MtOPR05-3 SDTAQGYP------DTPGIWTKEHVEAWKPIVDAVHAKGATFFCQIWHVGRVSNS-ICQPNGQAPISSTN
MtOPR05-4 SDTAQGYP------DTPGIWTKEHVEAWKPIVDAVHAKGATFFCQIWHVGRVSDT-VFQPNGQAPISPTD
MtOPR05-5 SDTAQGYP------DTPGIWTKEHVEAWKPIVDAVHAKGATIFCQIWHVGRVSDS-VFQPNGQAPISSTD
MtOPR05-6 SDTAQGYP------NTPGIWTKEQVDAWKPIVDAVHDKGGVFFCQIWHVGRVSDT-VYQPNGQAPISSTD
PsOPR3 SDTAQGYP------DTPGIWTEEQVEAWKPIVNGVHEKGGVFFCQIWHTGRASHV-DYQPNGQAPVSSTD
PsOPR2 SDSAQGYP------NTPGIWTEEQVEAWKPIVNGVHEKGGVFFCQIWHSGRASHV-DYQPNGQAPLSSTD
PsOPR1 SDSAQGYP------NTPGIWTEEQVEAWKPIVNGVHEKGGVFFCQIWHSGRASHV-DYQPNGQAPLSSTD
PpOPR4 NETSNGYP------HTPGIWTQEQMEAWKPIVKAVHDKGGVFFCQIWHVGRVSHA-SYQPNGAAPVSSSS
PpOPR5 DATSNGYP------HTPGIWTQEQVEAWKPIVKAVHDKGGIFLCQIWHVGRVSHT-SYHPNGAAPPSSTN
PpOPR2 SEDSNGYP------HTPGIWTQEHVEAWKPIVQAVHEKGGIFICQIWHVGRVSHT-SYN-KGNAPVSSTN
PpOPR1 SDCSYGYP------HTPGAYTDEQVEAWKPIVKAVHDKGGIFICQLWHVGRATHN-SYQPDGVFPVSSTN
SmOPR1 SPTGFGFP------CTPGVFSEEQVEAWKPIVKAVHDKGGIFFLQIWHVGRVSHT-SYQPHGEAPLSSSN
SmOPR2 SPSGLGYP------HSPGIWSEEQVEAWKPIVQAVHDKGAVFFLQIWHVGRVSHT-SYQPNGQAPVSSTN
SmOPR3 SDTSMGYS------CTPGIWTQEQVEAWKPIVKAVHAKGAVFFLQIWHVGRLSHT-SYQPKGQAPISCSS
SmOPR4 SDTSMGYP------CTPGIWTQEQVEAWKPIVKAVHDKGAVFFLQIWHVGRLSHT-SYQPKGQAPISCSS
SmOPR6 SDTSMGYP------CTPGIWTQEQVEAWKPIVKAVHDKGAVFFLQIWHVGRLSHT-SYQPKGQAPISCSS
PpOPR3 APEGYGFA------HTPGIFTEEQVNAWRKVVDAVHVKGCYIYCQIWHVGRASHK-YHQPNGAAPVSPTS
SmOPR5 SQQGIGWP------HSPGIWSDEQVQAWKPVVKAVHDKGAIFFCQLWHVGRASHT-CFQPRGQAPVSSTN
PpOPR6 AANGHGFV------NTAGIYTSEQIESWKAVTQGVHKRGAIFFLQLWHVGRASHT-ELQPGNAAPISPTS
PtOPR4 SPTAPGFP------HVPGIYSDAQVEAWKKVVNAVHAKGSIIFCQLWHVGRASHQ-VYQPGGAAAISSTN
PtOPR5 SPTAPGFP------HVPGIYSDAQVEAWKKVVDAVHAKGSVIFCQLWHVGRASHQ-VYQPGGVPPISSTN
AtOPR02-1 SPGSAGFP------HVPGIYSDEQVEAWKQVVEAVHAKGGFIFCQLWHVGRASHA-VYQPNGGSPISSTN
OsOPR08-1 SPAGPGFP------HVPGIYNQEQTDAWKKVVDAVHAKGGIFFCQLWHVGRASHQ-VYQPNGAAPISSTD
ZmOPR01-1 SPSGPGFP------RVPGIYNQEQTDAWRKVVDAVHAKGAIFFCQLWHVGRASHQ-VYQPGAAAPISSTD
SbOPR07-1 SPSGPGFP------RVPGIYNQEQTDAWRKVVDAVHAKGAIFFCQLWHVGRASHQ-VYQPGGAAPISSTD
ZmOPR04-1 SPAGPGFP------RVPGIYNQEQTDAWKKVVDAVHAKGAIFFCQLWHVGRASHQ-VYQPGGSAPISSTD
VcOPR1 APDGLGYM------NSPGVYSSEQLEAWKPVVKAVKDKGALFFCQLWHCGRASHP-ELQPGGAAPLSSST
CrOPR2 TAEGLGYL------NTPGLYTPEQLESWKPVTKAVHDKGGVFFCQLWHTGRVSHS-DLQPGNQLPISSSS
CrOPR3 SAEAYGYL------NSPGLYTPEQLQAWKPIVRTVRDKGAVFFCQLWHTGRASHN-DLQPHGTAPISSST
CrOPR1 RPDGRGYV------RAPGLATEAQATAWKPVVQKVHGKGGILYAQLFHAGRVSHS-SL-VGSKPTVCASP
VcOPR2 VPEGRGYV------RAPGMATDPQAAAWRPVVERVHAKGGHIYMQLFHAGRVSHS-SL-VGKQLPVSASP
ScOYE1 SPQAGGYD------NAPGVWSEEQMVEWTKIFNAIHEKKSFVWVQLWVLGWAAFPDNLARDGLRYDSASD
α2
β3

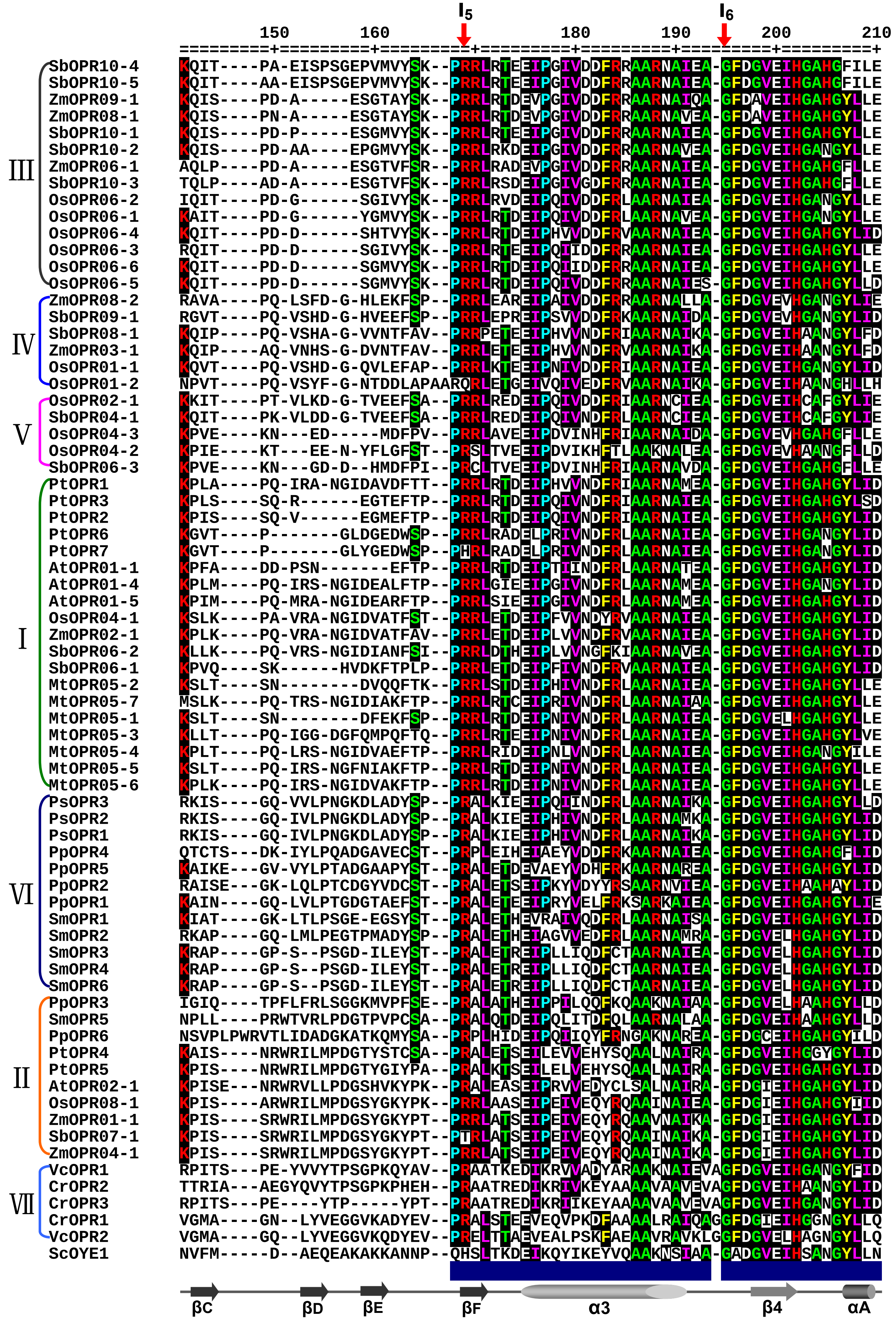

I5
I6
150
160
180
190
200
210
III
IV
V
I
VI
II
VII
SbOPR10-4 KQIT----PA-EISPSGEPVMVYSK--PRRLRTEEIPGIVDDFRRAARNAIEA-GFDGVEIHGAHGFILE
SbOPR10-5 KQIT----AA-EISPSGEPVMVYSK--PRRLRTEEIPGIVDDFRRAARNAIEA-GFDGVEIHGAHGFILE
ZmOPR09-1 KQIS----PD-A-----ESGTAYSK--PRRLRTDEVPGIVDDFRRAARNAIQA-GFDAVEIHGAHGYLLE
ZmOPR08-1 KQIS----PN-A-----ESGTAYSK--PRRLRTDEVPGIVDDFRRAARNAVEA-GFDAVEIHGAHGYLLE
SbOPR10-1 KQIS----PD-P-----ESGMVYSK--PRRLRTEEIPGIVDDFRRAARNAIEA-GFDGVEIHGAHGYLLE
SbOPR10-2 KQIS----PD-AA----EPGMVYSK--PRRLRKDEIPGIVDDFRRAARNAVEA-GFDGVEIHGANGYLLE
ZmOPR06-1 AQLP----PD-A-----ESGTVFSR--PRRLRADEVPGIVDDFRRAARNAIEA-GFDGVEIHGAHGFLLE
SbOPR10-3 TQLP----AD-A-----ESGTVFSK--PRRLRSDEIPGIVGDFRRAARNAIEA-GFDGVEIHGAHGFLLE
OsOPR06-2 IQIT----PD-G------SGIVYSK--PRRLRVDEIPQIVDDFRLAARNAIEA-GFDGVEIHGANGYLLE
OsOPR06-1 KAIT----PD-G------YGMVYSK--PRRLRTDEIPQIVDDFRLAARNAVEA-GFDGVEIHGANGYLLE
OsOPR06-4 KQIT----PD-D------SHTVYSK--PRRLRTDEIPHVVDDFRVAARNAIEA-GFDGVEIHGAHGYLID
OsOPR06-3 RQIT----PD-D------SGIVYSK--PRRLRTEEIPQIIDDFRRAARNAIEA-GFDGVEIHGAHGYLLE
OsOPR06-6 KQIT----PD-D------SGMVYSK--PRRLRTDEIPQIIDDFRRAARNAIEA-GFDGVEIHGAHGYLLE
OsOPR06-5 KQIT----PD-D------SGMVYSK--PRRLRTDEIPQIVDDFRRAARNAIES-GFDGVEIHGAHGYLLD
ZmOPR08-2 RAVA----PQ-LSFD-G-HLEKFSP--PRRLEAREIPAIVDDFRRAARNALLA-GFDGVEVHGANGYLIE
SbOPR09-1 RGVT----PQ-VSHD-G-HVEEFSP--PRRLEPREIPSVVDDFRKAARNAIDA-GFDGVEVHGANGYLIE
SbOPR08-1 KQIP----PQ-VSHA-G-VVNTFAV--PRRPETEEIPHVVNDFRIAARNAIKA-GFDGVEIHAANGYLFD
ZmOPR03-1 KQIP----AQ-VNHS-G-DVNTFAV--PRRLETEEIPHVVNDFRVAARNAIKA-GFDGVEIHAANGYLFD
OsOPR01-1 KQVT----PQ-VSHD-G-QVLEFAP--PRRLKTEEIPNIVDDFRIAARNAIEA-GFDGVEIHGANGYLID
OsOPR01-2 NPVT----PQ-VSYF-G-NTDDLAPAARQRLETGEIVQIVEDFRVAARNAIKA-GFDGVEIHAANGHLLH
OsOPR02-1 KKIT----PT-VLKD-G-TVEEFSA--PRRLREDEIPQIVDDFRIAARNCIEA-GFDGVEIHCAFGYLIE
SbOPR04-1 KQIT----PK-VLDD-G-TVEEFSA--PRRLREDEIPQIVNDFRLAARNCIEA-GFDGVEIHCAFGYLIE
OsOPR04-3 KPVE----KN---ED-----MDFPV--PRRLAVEEIPDVINHFRIAARNAIDA-GFDGVEVHGAHGFLLE
OsOPR04-2 KPIE----KT---EE-N-YFLGFST--PRSLTVEEIPDVIKHFTLAAKNALEA-GFDGVEVHAANGFLLD
SbOPR06-3 KPVE----KN---GD-D--HMDFPI--PRCLTVEEIPDVINHFRIAARNAVDA-GFDGVEIHGAHGFLLE
PtOPR1 KPLA----PQ-IRA-NGIDAVDFTT--PRRLRTDEIPHVVNDFRIAARNAMEA-GFDGVEIHGAHGYLID
PtOPR3 KPLS----SQ-R------EGTEFTP--PRRLRTDEIPQIVNDFRIAARNAIEA-GFDGVEIHGAHGYLSD
PtOPR2 KPIS----SQ-V------EGMEFTP--PRRLRTDEIPQIVNDFRIAARNAIEA-GFDGVEIHGAHGYLID
PtOPR6 KGVT----P-------GLDGEDWSP--PRRLRADELPRIVNDFRLAARNAIEA-GFDGVEIHGANGYLID
PtOPR7 KGVT----P-------GLYGEDWSP--PHRLRADELPRIVNDFRLAARNAIEA-GFDGVEIHGANGYLID
AtOPR01-1 KPFA----DD-PSN-------EFTP--PRRLRTDEIPTIINDFRLAARNATEA-GFDGVEIHGAHGYLID
AtOPR01-4 KPLM----PQ-IRS-NGIDEALFTP--PRRLGIEEIPGIVNDFRLAARNAMEA-GFDGVEIHGANGYLID
AtOPR01-5 KPIM----PQ-MRA-NGIDEARFTP--PRRLSIEEIPGIVNDFRLAARNAMEA-GFDGVEIHGAHGYLID
OsOPR04-1 KSLK----PA-VRA-NGIDVATFST--PRRLETDEIPFVVNDYRVAARNAIEA-GFDGVEIHGAHGYLID
ZmOPR02-1 KPLK----PQ-VRA-NGIDVATFAV--PRRLETDEIPLVVNDFRVAARNAIEA-GFDGVEIHGAHGYLID
SbOPR06-2 KLLK----PQ-VRS-NGIDIANFSI--PRRLDTHEIPLVVNGFKIAARNAVEA-GFDGVEIHGAHGYLID
SbOPR06-1 KPVQ----SK------HVDKFTPLP--PRRLETDEIPFIVNDFRVAARNAIEA-GFDGVEIHGAHGYLID
MtOPR05-2 KSLT----SN--------DVQQFTK--PRRLSTDEIPHIVNDFRLAARNAIEA-GFDGVEIHGAHGYLLE
MtOPR05-7 MSLK----PQ-TRS-NGIDIAKFTP--PRRLRTCEIPRIVNDFRLAARNAIAA-GFDGVEIHGAHGYLLE
MtOPR05-1 KSLT----SN--------DFEKFSP--PRRLRTDEIPNIVNDFRLAARNAIEA-GFDGVELHGAHGYLLE
MtOPR05-3 KLLT----PQ-IGG-DGFQMPQFTQ--PRRLRTEEIPNIVNDFRLAARNAIEA-GFDGVEIHGAHGYLVE
MtOPR05-4 KPLT----PQ-LRS-NGIDVAEFTP--PRRLRIDEIPNLVNDFRLAARNAIEA-GFDGVEIHGANGYILE
MtOPR05-5 KSLT----PQ-IRS-NGFNIAKFTP--PRRLRTDEIPNIVNDFRLAARNAIEA-GFDGVEIHGAHGYLLE
MtOPR05-6 KPLK----PQ-IRS-NGIDVAKFTP--PRRLRTDEIPNIVNDFRLAARNAIEA-GFDGVEIHGAHGYLLE
PsOPR3 RKIS----GQ-VVLPNGKDLADYSP--PRALKIEEIPQIINDFRLAARNAIKA-GFDGVEIHGAHGYLLD
PsOPR2 RKIS----GQ-IVLPNGKDLADYSP--PRALKIEEIPHIVNDFRLAARNAMKA-GFDGVEIHGAHGYLID
PsOPR1 RKIS----GQ-IVLPNGKDLADYSP--PRALKIEEIPHIVNDFRLAARNAIKA-GFDGVEIHGAHGYLID
PpOPR4 QTCTS---DK-IYLPQADGAVECST--PRPLEIHEIAEYVDDFRKAARNAIEA-GFDGVEIHGAHGFLID
PpOPR5 KAIKE---GV-VYLPTADGAAPYST--PRALETDEVAEYVDHFRKAARNAREA-GFDGVEIHGAHGYLID
PpOPR2 RAISE---GK-LQLPTCDGYVDCST--PRALETSEIPKYVDYYRSAARNVIEA-GFDGVEIHAAHAYLID
PpOPR1 KAIN----GQ-LVLPTGDGTAEFST--PRALETEEIPRYVELFRKSARKAIEA-GFDGVEIHGAHGYLIE
SmOPR1 KIAT----GK-LTLPSGE-EGSYST--PRALETHEVRAIVQDFRLAARNAISA-GFDGVEIHGAHGYLID
SmOPR2 RKAP----GQ-LMLPEGTPMADYSP--PRALETHEIAGVVEDFRLAARNAMRA-GFDGVELHGAHGYLID
SmOPR3 KRAP----GP-S--PSGD-ILEYST--PRALETREIPLLIQDFCTAARNAIEA-GFDGVELHGAHGYLID
SmOPR4 KRAP----GP-S--PSGD-ILEYST--PRALETREIPLLIQDFCTAARNAIEA-GFDGVELHGAHGYLID
SmOPR6 KRAP----GP-S--PSGD-ILEYST--PRALETREIPLLIQDFCTAARNAIEA-GFDGVELHGAHGYLID
PpOPR3 IGIQ----TPFLFRLSGGKMVPFSE--PRALATHEIPPILQQFKQAAKNAIAA-GFDGVELHAAHGYLLD
SmOPR5 NPLL----PRWTVRLPDGTPVPCSA--PRALQTDEIPQLITDFQLAARNALAA-GFDGVEIHAAHGYLLD
PpOPR6 NSVPLPWRVTLIDADGKATKQMYSA--PRPLHIDEIPQIIQYFRNGAKNAREA-GFDGCEIHGAHGYILD
PtOPR4 KAIS----NRWRILMPDGTYSTCSA--PRALETSEILEVVEHYSQAALNAIRA-GFDGVEIHGGYGYLID
PtOPR5 KPIS----NRWRILMPDGTYGIYPA--PRALKTSEILELVEHYSQAALNAIRA-GFDGVEIHGAHGYLID
AtOPR02-1 KPISE---NRWRVLLPDGSHVKYPK--PRALEASEIPRVVEDYCLSALNAIRA-GFDGIEIHGAHGYLID
OsOPR08-1 KPIS----ARWRILMPDGSYGKYPK--PRRLAASEIPEIVEQYRQAAINAIEA-GFDGIEIHGAHGYIID
ZmOPR01-1 KPIS----SRWRILMPDGSYGKYPT--PRRLATSEIPEIVEQYRQAAVNAIKA-GFDGIEIHGAHGYLID
SbOPR07-1 KPIS----SRWRILMPDGSYGKYPT--PTRLATSEIPEIVEQYRQAAINAIKA-GFDGIEIHGAHGYLID
ZmOPR04-1 KPIS----SRWRILMPDGSYGKYPT--PRRLATSEIPEIVEQYRQAAINAIKA-GFDGIEIHGAHGYLID
VcOPR1 RPITS---PE-YVVYTPSGPKQYAV--PRAATKEDIKRVVADYARAAKNAIEVAGFDGVEIHGANGYFID
CrOPR2 TTRIA---AEGYQVYTPSGPKPHEH--PRAATREDIKRIVKEYAAAAVAAVEVAGFDGVEIHAANGYLID
CrOPR3 RPITS---PE----YTP-----YPT--PRAATREDIKRIIKEYAAAAVAAVEVAGFDGVEIHGANGYLID
CrOPR1 VGMA----GN--LYVEGGVKADYEV--PRALSTEEVEQVPKDFAAAALRAIQAGGFDGIEIHGGNGYLLQ
VcOPR2 VGMA----GQ--LYVEGGVKQDYEV--PRELTTAEVEALPSKFAEAAVRAVKLGGFDGVELHAGNGYLLQ
ScOYE1 NVFM-----D--AEQEAKAKKANNP--QHSLTKDEIKQYIKEYVQAAKNSIAA-GADGVEIHSANGYLLN
βC
βD
βE
βF
α3
β4
αA

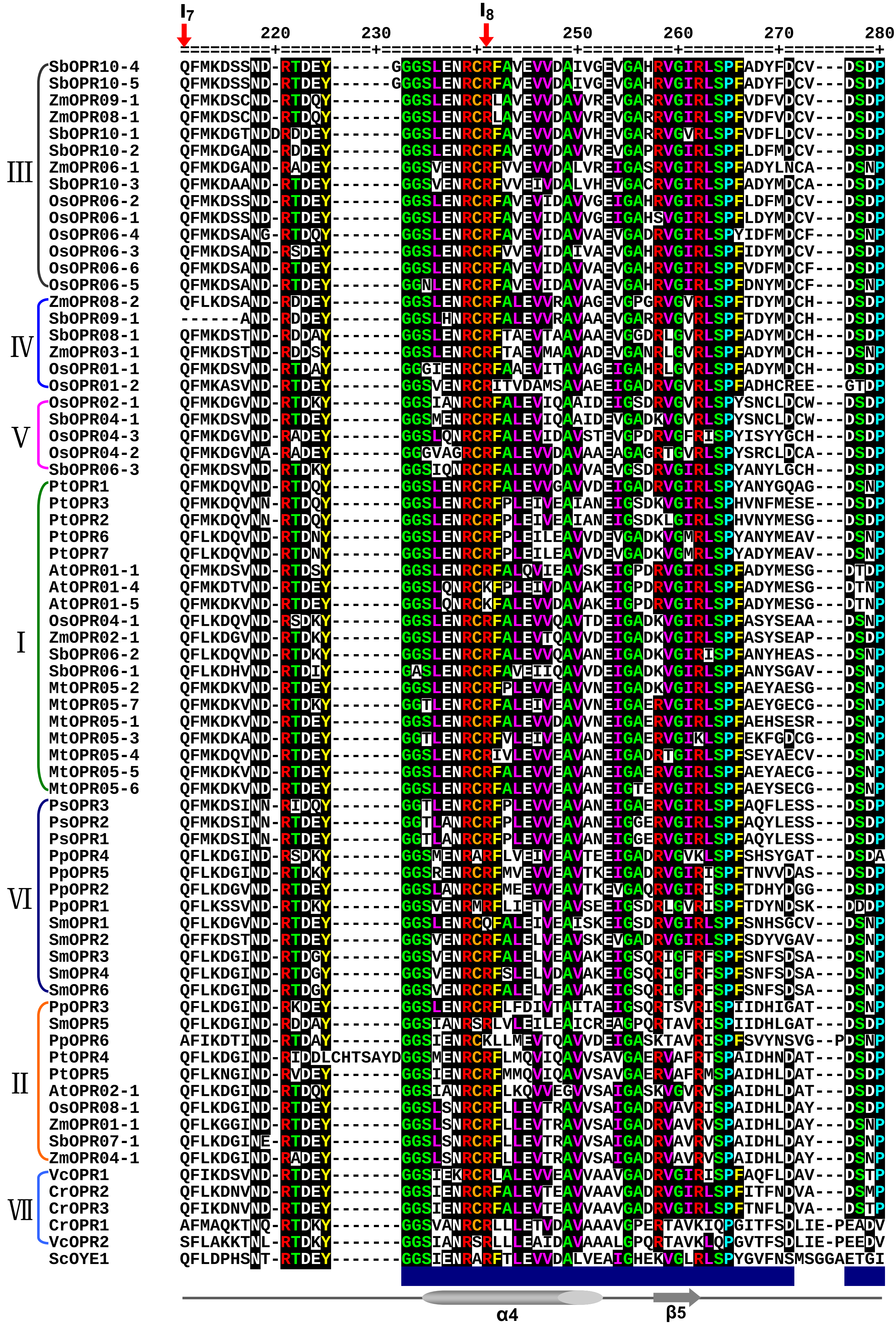

I7
I8
220 230 250 260 270 280
=========+=========+=========+=========+=========+=========+=========+=========+
III
IV
V
I
VI
II
VII
SbOPR10-4 QFMKDSSND-RTDEY------GGGSLENRCRFAVEVVDAIVGEVGAHRVGIRLSPFADYFDCV---DSDP
SbOPR10-5 QFMKDSSND-RTDEY------GGGSLENRCRFAVEVVDAIVGEVGAHRVGIRLSPFADYFDCV---DSDP
ZmOPR09-1 QFMKDSCND-RTDQY-------GGSLENRCRLAVEVVDAVVREVGARRVGIRLSPFVDFVDCV---DSDP
ZmOPR08-1 QFMKDSCND-RTDQY-------GGSLENRCRLAVEVVDAVVREVGARRVGIRLSPFVDFVDCV---DSDP
SbOPR10-1 QFMKDGTNDDRDDEY-------GGSLENRCRFAVEVVDAVVHEVGARRVGVRLSPFVDFLDCV---DSDP
SbOPR10-2 QFMKDGAND-RDDEY-------GGSLENRCRFAVEVVDAVVREVGAPRVGIRLSPFLDFMDCV---DSDP
ZmOPR06-1 QFMKDGAND-RADEY-------GGSVENRCRFVVEVVDALVREIGASRVGIRLSPFADYLNCA---DSNP
SbOPR10-3 QFMKDAAND-RTDEY-------GGSVENRCRFVVEIVDALVHEVGACRVGIRLSPFADYMDCA---DSDP
OsOPR06-2 QFMKDSSND-RTDEY-------GGSLENRCRFAVEVIDAVVGEIGAHRVGIRLSPFLDFMDCV---DSDP
OsOPR06-1 QFMKDSSND-RTDEY-------GGSLENRCRFAVEVIDAVVGEIGAHSVGIRLSPFLDYMDCV---DSDP
OsOPR06-4 QFMKDSANG-RTDQY-------GGSLENRCRFAVEVIDAVVAEVGADRVGIRLSPYIDFMDCF---DSNP
OsOPR06-3 QFMKDSAND-RSDEY-------GGSLENRCRFVVEVIDAIVAEVGAHRVGIRLSPFIDYMDCV---DSDP
OsOPR06-6 QFMKDSAND-RTDEY-------GGSLENRCRFAVEVIDAVVAEVGAHRVGIRLSPFVDFMDCF---DSDP
OsOPR06-5 QFMKDSAND-RTDEY-------GGNLENRCRFAVEVIDAVVAEVGAHRVGIRLSPFDNYMDCF---DSNP
ZmOPR08-2 QFLKDSAND-RDDEY-------GGSLENRCRFALEVVRAVAGEVGPGRVGVRLSPFTDYMDCH---DSDP
SbOPR09-1 ------AND-RDDEY-------GGSLHNRCRFALEVVRAVAAEVGARRVGVRLSPFTDYMDCH---DSDP
SbOPR08-1 QFMKDSTND-RDDAY-------GGSLENRCRFTAEVTAAVAAEVGGDRLGVRLSPFADYMDCH---DSDP
ZmOPR03-1 QFMKDSTND-RDDSY-------GGSLENRCRFTAEVMAAVADEVGANRLGVRLSPFADYMDCH---DSNP
OsOPR01-1 QFMKDSVND-RTDAY-------GGGIENRCRFAAEVITAVAGEIGAHRLGVRLSPFADYMDCH---DSDP
OsOPR01-2 QFMKASVND-RTDEY-------GGSVENRCRITVDAMSAVAEEIGADRVGVRLSPFADHCREE---GTDP
OsOPR02-1 QFMKDGVND-RTDKY-------GGSIANRCRFALEVIQAAIDEIGSDRVGVRLSPYSNCLDCW---DSDP
SbOPR04-1 QFMKDSVND-RTDEY-------GGSMENRCRFALEVIQAAIDEVGADKVGVRLSPYSNCLDCW---DSDP
OsOPR04-3 QFMKDGVND-RADEY-------GGSLQNRCRFALEVIDAVSTEVGPDRVGFRISPYISYYGCH---DSDP
OsOPR04-2 QFMKDGVNA-RADEY-------GGGVAGRCRFALEVVDAVAAEAGAGRTGVRLSPYSRCLDCA---DSDP
SbOPR06-3 QFMKDSVND-RTDKY-------GGSIQNRCRFALEVVDAVVAEVGSDRVGIRLSPYANYLGCH---DSDP
PtOPR1 QFMKDQVND-RTDQY-------GGSLENRCRFALEVVGAVVDEIGADRVGIRLSPYANYGQAG---DSNP
PtOPR3 QFMKDQVNN-RTDQY-------GGSLENRCRFPLEIVEAIANEIGSDKVGIRLSPHVNFMESE---DSDP
PtOPR2 QFMKDQVNN-RTDQY-------GGSLENRCRFPLEIVEAIANEIGSDKLGIRLSPHVNYMESG---DSDP
PtOPR6 QFLKDQVND-RTDNY-------GGSLENRCRFPLEILEAVVDEVGADKVGMRLSPYANYMEAV---DSNP
PtOPR7 QFLKDQVND-RTDNY-------GGSLENRCRFPLEILEAVVDEVGADKVGMRLSPYADYMEAV---DSNP
AtOPR01-1 QFMKDSVND-RTDSY-------GGSLENRCRFALQVIEAVSKEIGPDRVGIRLSPFADYMESG---DTDP
AtOPR01-4 QFMKDTVND-RTDEY-------GGSLQNRCKFPLEIVDAVAKEIGPDRVGIRLSPFADYMESG---DTNP
AtOPR01-5 QFMKDKVND-RTDEY-------GGSLQNRCKFALEVVDAVAKEIGPDRVGIRLSPFADYMESG---DTNP
OsOPR04-1 QFLKDQVND-RSDKY-------GGSLENRCRFALEVVQAVTDEIGADKVGIRLSPFASYSEAA---DSNP
ZmOPR02-1 QFLKDGVND-RTDKY-------GGSLENRCRFALEVTQAVVDEIGADKVGIRLSPFASYSEAP---DSDP
SbOPR06-2 QFLKDQVND-RTDKY-------GGSLENRCRFALEVVQAVANEIGADKVGIRISPFANYHEAS---DSNP
SbOPR06-1 QFLKDHVND-RTDIY-------GASLENRCRFAVEIIQAVVDEIGADKVGIRLSPFANYSGAV---DSNP
MtOPR05-2 QFMKDKVND-RTDEY-------GGSLENRCRFPLEVVEAVVNEIGADKVGIRLSPFAEYAESG---DSNP
MtOPR05-7 QFMKDKVND-RTDKY-------GGTLENRCRFALEIVEAVVNEIGAERVGIRLSPFAEYGECG---DSNP
MtOPR05-1 QFMKDKVND-RTDEY-------GGSLENRCRFALEVVDAVVNEIGAERVGIRLSPFAEHSESR---DSNP
MtOPR05-3 QFMKDKAND-RTDEY-------GGTLENRCRFVLEIVEAVANEIGAERVGIKLSPFEKFGDCG---DSNP
MtOPR05-4 QFMKDQVND-RTDEY-------GGSLENRCRIVLEVVEAVANEIGADRTGIRLSPFSEYAECV---DSNP
MtOPR05-5 QFMKDKVND-RTDEY-------GGSLENRCRFALEVVEAVANEIGAERVGIRLSPFAEYAECG---DSNP
MtOPR05-6 QFMKDKVND-RTDEY-------GGSLENRCRFALEVVEAVANEIGTERVGIRLSPFAEYSECG---DSNP
PsOPR3 QFMKDSINN-RIDQY-------GGTLENRCRFPLEVVEAVANEIGAERVGIRLSPFAQFLESS---DSDP
PsOPR2 QFMKDSINN-RTDEY-------GGTLANRCRFPLEVVEAVANEIGGERVGIRLSPFAQYLESS---DSDP
PsOPR1 QFMKDSINN-RTDEY-------GGTLANRCRFPLEVVEAVANEIGGERVGIRLSPFAQYLESS---DSDP
PpOPR4 QFLKDGIND-RSDKY-------GGSMENRARFLVEIVEAVTEEIGADRVGVKLSPFSHSYGAT---DSDA
PpOPR5 QFLKDGIND-RTDKY-------GGSRENRCRFMVEVVEAVTKEIGADRVGIRISPFTNVVDAS---DSDP
PpOPR2 QFLKDGVND-RTDEY-------GGSLANRCRFMEEVVEAVTKEVGAQRVGIRISPFTDHYDGG---DSDP
PpOPR1 QFLKSSVND-RTDKY-------GGSVENRMRFLIETVEAVSEEIGSDRLGVRISPFTDYNDSK---DDDP
SmOPR1 QFLKDGVND-RTDEY-------GGSLENRCQFALEIVEAISKEIGSDRVGIRLSPFSNHSGCV---DSNP
SmOPR2 QFFKDSTND-RTDEY-------GGSVENRCRFALELVEAVSKEVGADRVGIRLSPFSDYVGAV---DSNP
SmOPR3 QFLKDGIND-RTDGY-------GGSVENRCRFALELVEAVAKEIGSQRIGFRFSPFSNFSDSA---DSNP
SmOPR4 QFLKDGIND-RTDGY-------GGSVENRCRFSLELVDAVAKEIGSQRIGFRFSPFSNFSDSA---DSNP
SmOPR6 QFLKDGIND-RTDGY-------GGSVENRCRFALELVEAVAKEIGSQRIGFRFSPFSNFSDSA---DSNP
PpOPR3 QFLKDGIND-RKDEY-------GGSLENRCRFLFDIVTAITAEIGSQRTSVRISPIIDHIGAT---DSNP
SmOPR5 QFLKDGIND-RDDAY-------GGSIANRSRLVLEILEAICREAGPQRTAVRISPIIDHLGAT---DSDP
PpOPR6 AFIKDTIND-RTDAY-------GGSIENRCKLLMEVTQAVVDEIGASKTAVRISPFSVYNSVG--PDSNP
PtOPR4 QFLKDGIND-RIDDLCHTSAYDGGSMENRCRFLMQVIQAVVSAVGAERVAFRTSPAIDHNDAT---DSDP
PtOPR5 QFLKNGIND-RVDEY-------GGSIENRCRFMMQVIQAVVSAVGAERVAFRMSPAIDHLDAT---DSDP
AtOPR02-1 QFLKDGIND-RTDQY-------GGSIANRCRFLKQVVEGVVSAIGASKVGVRVSPAIDHLDAT---DSDP
OsOPR08-1 QFLKDGIND-RTDEY-------GGSLSNRCRFLLEVTRAVVSAIGADRVAVRISPAIDHLDAY---DSDP
ZmOPR01-1 QFLKGGIND-RTDEY-------GGSLSNRCRFLLEVTRAVVSAIGADRVAVRVSPAIDHLDAY---DSNP
SbOPR07-1 QFLKDGINE-RTDEY-------GGSLSNRCRFLLEVTRAVVSAIGADRVAVRVSPAIDHLDAY---DSNP
ZmOPR04-1 QFLKDGIND-RADEY-------GGSLSNRCRFLLEVTRAVVSAIGADRVAVRVSPAIDHLDAY---DSNP
VcOPR1 QFIKDSVND-RTDEY-------GGSIEKRCRLALEVVEAVVAAVGADRVGIRISPFAQFLDAV---DSTP
CrOPR2 QFLKDNVND-RTDEY-------GGSIENRCRFALEVTEAVVAAVGADRVGIRLSPFITFNDVA---DSMP
CrOPR3 QFIKDNVND-RTDEY-------GGSIENRCRFALEVTEAVVAAVGADRVGIRLSPFTNFLDVA---DSTP
CrOPR1 AFMAQKTNQ-RTDKY-------GGSVANRCRLLLETVDAVAAAVGPERTAVKIQPGITFSDLIE-PEADV
VcOPR2 SFLAKKTNL-RTDKY-------GGSIANRSRLLLEAIDAVAAALGPQRTAVKLQPGVTFSDLIE-PEEDV
ScOYE1 QFLDPHSNT-RTDEY-------GGSIENRARFTLEVVDALVEAIGHEKVGLRLSPYGVFNSMSGGAETGI
α4
β5

```
                 I9                       I10
                 290    ↓      300       310      ↓  320       330       340       350
              =========+=========+=========+=========+=========+=========+=========+
III
SbOPR10-4     VALGHYMIQQLNKHEG------FLYCHMVEPRMAM----VDG---------RRQIPHRLLPFRKAFH---
SbOPR10-5     VALGHYMIQQLNKHEG------FLYCHMVEPRMAI----VDG---------RRQIPHRLLPFRKAFH---
ZmOPR09-1     VALGHYMVQQLNRHSG------LLYCHMVEPRMAT----VDG---------RRQIPHGLLPFRKAFH---
ZmOPR08-1     VALGHYMVQQLNRHSG------LLYCHMVEPRIT-----AHG---------RRQIPHGLLPFRKAFH---
SbOPR10-1     LALGEYMVQQLNRHEG------LLYCHMVEPRMAN----VDG---------RRQIPHRLLPFRKAFN---
SbOPR10-2     AALADYMVRQLNRHEG------FLYCHMVEPRMAV----VDG---------RRQIPHRLLPFRETFK---
ZmOPR06-1     LALGHYMVEQLNRHEG------FLYCHMVEPRMAI----VDD---------RRQIPHGLLPFKAAFN---
SbOPR10-3     MALGDYMVRQLNKHEG------FLYCHMVEPRMSI----VDD---------RRQIPHRLLPFKTVFS---
OsOPR06-2     EALGSYMVEQLNKHEG------FLYCHMVEPRMAI----VDG---------RRQIQHGLLPFRKAFK---
OsOPR06-1     EALGSYMVEQLNKHED------FLYCHMVEPRMAI----VDG---------RRQIQHGLLPFRKQFN---
OsOPR06-4     EALGSYMVRQLNKHPE------LLYCHMVEPRMAT----VEG---------RRKINHGLLPFRKQFN---
OsOPR06-3     VALGSYMVQQLNKHPG------FLYCHMVEPRMAI----VEG---------RRKITHGLLPFRKLFN---
OsOPR06-6     VALGSYMVQQLNKHPG------FLYCHMVEPRMAI----IEG---------RRKIAHGLLPFRKQFN---
OsOPR06-5     VALGSYMVQQLNKHPG------FLYCHMVEPGMAI----VEG---------RRKITHGLLPFRKQFN---
IV
ZmOPR08-2     EALAGYLVRSLSD-VG------VLYCHMIEPRMAI----VDG---------RRQIPHRLRPFREAFR---
SbOPR09-1     EALAGYMVEKLSE-LG------VLYCHMIEPRMAI----VDG---------RRQIPHRLRPFREKFR---
SbOPR08-1     EALGLHVIDKTLNPLG------VLYCHMVEARMRV---NPEDGN-------KMTLQQMLLPFRRAFR---
ZmOPR03-1     EALALHVIDETLNPLG------VLYCHMVEPRMRV---NPDDG--------IMTLEHMLLPFRRAFR---
OsOPR01-1     EVLALRVIGLMNN-LG------VLYCHMIEPRMCV----GAGEDGSK----PVIAHGRLLPFRKAFR---
OsOPR01-2     EEVALHLIGVMNG-LG------VLYCHVIEPRCCV----SSIEEHRA----RRNVPHRLLPFRRAFH---
V
OsOPR02-1     DALGLYMIQAMSK-LG------VLYCSMVEPEVVK----VDG---------KVQIPYKLWHFRKVFA---
SbOPR04-1     DALGLYMIHAMNK-LG------VLYCSMVEPEVVK----VDG---------KVQIPHKLLHFRKAFS---
OsOPR04-3     DALGVYMARELDR-RG------VLYCSAVEPEMVAATTVVDGETTTTMSRRMMIPHRLHGMREAFRR--
OsOPR04-2     DALAAHMARELGS-RG------VLYCNVVEPEMVA--TPAEGGSG----GETMRIPHRLRAVREAFA---
SbOPR06-3     DRLGVYMAQELNR-YN------ILYCSAVEPEMV----MVDG---------KMKIPHSLQEMRKAFN---
I
PtOPR1        GALGLYMVESLNK-YG------ILFCHMVEPRMKT----VGE---------RVESPHSLLPMRKAFN---
PtOPR3        EALGLHMVKSLNK-YG------IAYCHMVEPRMKI----GAG---------NTKFSESLLPMRKAFN---
PtOPR2        EALGLYMVKSLNK-YG------IAYCHMVEPRMKI----GAG---------NPKFSESLLPMRKAFN---
PtOPR6        EELGLYMANALNK-FG------ILYLHVIEPRMVK----AND---------IYETPHSLLPMKNAFE---
PtOPR7        EELGLYMANALNK-FG------ILYLHVIEPRMVK----AND---------IYETPHSLLPMKNAFE---
AtOPR01-1     KRLGLYMAKSLNR-FE------ILYCHMIEPRMKT----VSE---------IFECRESLTPMRNAFN---
AtOPR01-4     GALGLYMAESLNK-YG------ILYCHVIEARMKT----MGE---------VHACPHTLMPMRKAFK---
AtOPR01-5     EALGLYMVESLNK-YG------ILYCHMIEPRMKT----VGE---------IAACSHTLMPMREAFK---
OsOPR04-1     EALGLYMANALNK-FG------ILYCHMVEPRMVK----LGE---------KFETPHSLRPIRDAFK---
ZmOPR02-1     EALGLYMANALNE-FG------ILYCHMVEPRMVK----LGE---------KFETPYSLRPIRDAFR---
SbOPR06-2     EALGLYMAKALNK-FG------ILYLHMVEPRMIT----VGD---------KTETPYSLRLMRDAFE---
SbOPR06-1     EALGLYMANALNN-FG------VLYCHVVVPRMVK----NGE---------NFETPSHIGLMRSAFK---
MtOPR05-2     NELGLHMVNALNK-YN------ILYCHMVEPRIKS----ASE---------TIESPYSLVPMRKAFN---
MtOPR05-7     EELGLYMVNALSK-YN------ILYCHMVEPRMKT----ITE---------KVECLHSLVPMRKAFN---
MtOPR05-1     TELGLYMVNALNK-YN------ILYCHMVEPRIKS----NDE---------TIESPYSLVPMRKAFN---
MtOPR05-3     QELGLYMVNALNK-YG------ILYCHMVEPRMEI----LDE---------KTECHYSLVSIRKVFN---
MtOPR05-4     RELGLYMANALNK-YG------ILYCHVVEPRMKT----VNE---------RIECSHSLVPMRKAFK---
MtOPR05-5     NELGLYMVNALNK-YG------ILYCHMVEPRMKT----VGE---------KTECPDSLVPMRKAFK---
MtOPR05-6     KELGLYMVNALNK-YG------ILYCHMVEPRMIT----VGE---------KTECPDSLVPMRKAFK---
VI
PsOPR3        EALGLYMAETLNR-YN------ILYAHYVEPRLVT----TFE---------PVATNKSLLPMRKAFK---
PsOPR2        EALGLYMAEALNR-YN------ILYAHYVEPRLVT----TFE---------PVATNKSLFPMRKAFK---
PsOPR1        EALGLYMAEALNR-YN------ILYAHYVEPRLVT----TFE---------PVATNKSLFPMRKAFK---
PpOPR4        TGLSVFLAETLNR-YN------LLYLMGVEASG------CGDE----------ESAESLWPMRKAFK---
PpOPR5        NGLSVYIAETLNR-YN------LLYLHGVEPRF------HPERT--------VQSEESLWPMRKAYK---
PpOPR2        TGLSVYIANTLNK-YN------LLYLHVVEPRFTF----KSETL--------IESKHSIWPIRNAYR---
PpOPR1        VALGVSIAQALNK-YN------LMYLHCIEPRFSP----QETPH--------IETDKTLWPVRKAFK---
SmOPR1        QELGLYLIQALNS-AN------LVYVHLVEPRVNF----GCPN-----------DAHTLWPIVRAYK---
SmOPR2        LQLGLYMAEELNR-FN------ILYAHFVEPRMRV----GAAI---------APEAAGIWPLRKAFK---
SmOPR3        MKLAVFLAEALNR-YN------ILYV--AEPKIKL----SGEV------------ESELWPVKRAFK---
SmOPR4        MKLAVFLAEALNR-YN------ILYV--AEPKVKL----SGEV------------ESELWPVRRAFK---
SmOPR6        MKLAVFLAEALNR-YN------ILYV--AEPKVKL----SGEV------------ESELWPVRRAFK---
II
PpOPR3        VALGVHLARNLSR-FN------LAYLHVTEPRFHAQG-VSDTQ------------LNCKIFRDQYE---
SmOPR5        VALAHHLVAGFVK-LG------VLYLHATEPAF------SDWN------------RSLGFLREATSGSS
PpOPR6        EALFTYLHSKLDK-FG------LAYIHLTEPEFSF----IKDN---------FKWAPQYAHVTKRRIT---
PtOPR4        LNLGLSVIERINK-LQLQVGSKLTYLHVQR---------ELGRPGT-----EDVVAQTTRTWRRAYQ---
PtOPR5        LNLGLSVIERINK-LQLQVGSKLTYLHVTQPRYTASGQTESRRTGS-----EDEEAQMIRTWRRAYQ---
AtOPR02-1     LSLGLAVVGMLNK-LQGVNGSKLAYLHVTQPRYHAYGQTESGRQGS-----DEEEAKLMKSLRMAYN---
OsOPR08-1     IKLGMAVVERLNA-LQQQSGR-LAYLHVTQPRYTAYGQTESGQHGS-----AEEESRLMRTLRGTYQ---
ZmOPR01-1     LQLGLAVVERLNA-LQQEAGR-LAYLHVTQPRYTAYGQTESGQHGS-----AEEESRLMRAVRGAYR---
SbOPR07-1     LQLGLAVVDRLNA-LQQEVGRLLAYFHVTQPRYTAYGQTESGQHGS-----AEEESRLMRALRGAYR---
ZmOPR04-1     LQLGLAVVDRLNA-LQEETGR-LAYLHVTQPRYTAYGQTESGQHGS-----AEEESRLMRALRGAYR---
VII
VcOPR1        YATHTYLVEQLNK-YG------LSYIHMVEPRIL--GNTEVES------------SSDSLEPFRRVYK---
CrOPR2        YGTNVYLLEQLNK-LG------LAYVHMVEPRID--GITECA-------------TVDTLAPFRSVWK---
CrOPR3        YGTNVYLLEQLNK-FG------LAYVHMVEPRMAG-GCQRVCERA---------TTETLLPFRAVWK---
CrOPR1        KETLEYLGPELSK-RN------LAYVCLSTLNSEPYFRFLGLSEPN-------IKFDPFRLFRNTFT---
VcOPR2        RETLDYLGPELSK-RK------LAFVTLSSLNGEPYYKFAGLQAPN-------VNFDVFRDFRSKYS---
ScOYE1        VAQYAYVAGELEK-RAKA-GKRLAFVHLVEPRVTNPFLTEGE-----------GEYEGGSNDFVYSIWK
```

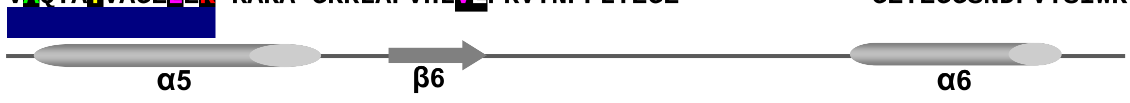

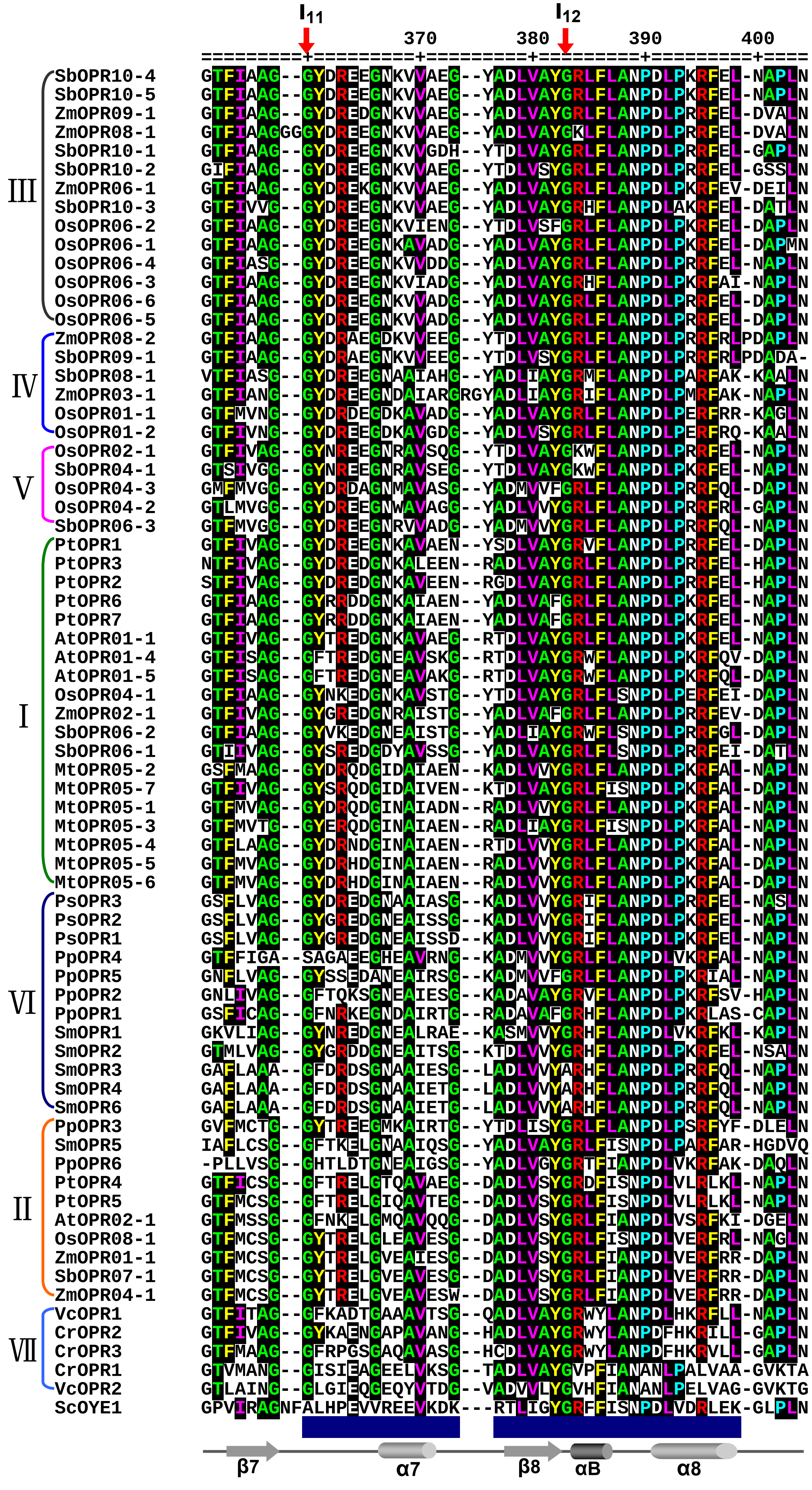
I11
I12
370
380
390
400
III
SbOPR10-4 GTFIAAG--GYDREEGNKVVAEG--YADLVAYGRLFLANPDLPKRFEL-NAPLN
SbOPR10-5 GTFIAAG--GYDREEGNKVVAEG--YADLVAYGRLFLANPDLPKRFEL-NAPLN
ZmOPR09-1 GTFIAAG--GYDREDGNKVVAEG--YADLVAYGRLFLANPDLPRRFEL-DVALN
ZmOPR08-1 GTFIAAGGGGYDREEGNKVVAEG--YADLVAYGKLFLANPDLPRRFEL-DVALN
SbOPR10-1 GTFIAAG--GYDREEGNKVVGDH--YTDLVAYGRLFLANPDLPRRFEL-GAPLN
SbOPR10-2 GIFIAAG--GYDREEGNKVVAEG--YTDLVSYGRLFLANPDLPRRFEL-GSSLN
ZmOPR06-1 GTFIAAG--GYDREKGNKVVAEG--YADLVAYGRLFLANPDLPKRFEV-DEILN
SbOPR10-3 GTFIVVG--GYDREEGNKVVAEG--YADLVAYGRHFLANPDLAKRFEL-DATLN
OsOPR06-2 GTFIAAG--GYDREEGNKVIENG--YTDLVSFGRLFLANPDLPKRFEL-DAPLN
OsOPR06-1 GTFIAAG--GYDREEGNKAVADG--YADLVAYGRLFLANPDLPKRFEL-DAPMN
OsOPR06-4 GTFIASG--GYDREEGNKVVDDG--YADLVAYGRLFLANPDLPRRFEL-NAPLN
OsOPR06-3 GTFIAAG--GYDREEGNKVIADG--YADLVAYGRHFLANPDLPKRFAI-NAPLN
OsOPR06-6 GTFIAAG--GYDREEGNKVVADG--YADLVAYGRLFLANPDLPRRFEL-DAPLN
OsOPR06-5 GTFIAAG--GYDREEGNKVVADG--YADLVAYGRLFLANPDLPRRFEL-DAPLN
IV
ZmOPR08-2 GTFIAAG--GYDRAEGDKVVEEG--YTDLVAYGRLFLANPDLPRRFRLPDAPLN
SbOPR09-1 GTFIAAG--GYDRAEGNKVVEEG--YTDLVSYGRLFLANPDLPRRFRLPDADA-
SbOPR08-1 VTFIASG--GYDREEGNAAIAHG--YADLIAYGRMFLANPDLPARFAK-KAALN
ZmOPR03-1 GTFIANG--GYDREEGNDAIARGRGYADLIAYGRIFLANPDLPMRFAK-NAPLN
OsOPR01-1 GTFMVNG--GYDRDEGDKAVADG--YADLVAYGRLFLANPDLPERFRR-KAGLN
OsOPR01-2 GTFIVNG--GYDREEGDKAVGDG--YADLVSYGRLFLANPDLPERFRQ-KAALN
V
OsOPR02-1 GTFIVAG--GYNREEGNRAVSQG--YTDLVAYGKWFLANPDLPRRFEL-NAPLN
SbOPR04-1 GTSIVGG--GYNREEGNRAVSEG--YTDLVAYGKWFLANPDLPKRFEL-NAPLN
OsOPR04-3 GMFMVGG--GYDRDAGNMAVASG--YADMVVFGRLFLANPDLPRRFQL-DAPLN
OsOPR04-2 GTLMVGG--GYDREEGNWAVAGG--YADLVVYGRLFLANPDLPRRFRL-GAPLN
SbOPR06-3 GTFMVGG--GYDREEGNRVVADG--YADMVVYGRLFLANPDLPRRFQL-NAPLN
I
PtOPR1 GTFIVAG--GYDREEGNKAVAEN--YSDLVAYGRVFLANPDLPRRFEL-DAPLN
PtOPR3 NTFIVAG--GYDREDGNKALEEN--RADLVAYGRLFLANPDLPRRFEL-HAPLN
PtOPR2 STFIVAG--GYDREDGNKAVEEN--RGDLVAYGRLFLANPDLPRRFEL-HAPLN
PtOPR6 GTFIAAG--GYRRDDGNKAIAEN--YADLVAFGRLFLANPDLPKRFEL-NAPLN
PtOPR7 GTFIAAG--GYRRDDGNKAIAEN--YADLVAFGRLFLANPDLPKRFEL-NAPLN
AtOPR01-1 GTFIVAG--GYTREDGNKAVAEG--RTDLVAYGRLFLANPDLPKRFEL-NAPLN
AtOPR01-4 GTFISAG--GFTREDGNEAVSKG--RTDLVAYGRWFLANPDLPKRFQV-DAPLN
AtOPR01-5 GTFISAG--GFTREDGNEAVAKG--RTDLVAYGRWFLANPDLPKRFQL-DAPLN
OsOPR04-1 GTFIAAG--GYNKEDGNKAVSTG--YTDLVAYGRLFLSNPDLPERFEI-DAPLN
ZmOPR02-1 GTFIVAG--GYGREDGNRAISTG--YADLVAFGRLFLANPDLPRRFEV-DAPLN
SbOPR06-2 GTFIAAG--GYVKEDGNEAISTG--YADLIAYGRWFLSNPDLPRRFGL-DAPLN
SbOPR06-1 GTIIVAG--GYSREDGDYAVSSG--YADLVAYGRLFLSNPDLPRRFEI-DATLN
MtOPR05-2 GSFMAAG--GYDRQDGIDAIAEN--KADLVVYGRLFLANPDLPKRFAL-NAPLN
MtOPR05-7 GTFIVAG--GYSRQDGIDAIVEN--KTDLVAYGRLFISNPDLPKRFAL-DAPLN
MtOPR05-1 GTFMVAG--GYDRQDGINAIADN--RADLVVYGRLFLANPDLPKRFAL-NAPLN
MtOPR05-3 GTFMVTG--GYERQDGINAIAEN--RADLIAYGRLFISNPDLPKRFAL-NAPLN
MtOPR05-4 GTFLAAG--GYDRNDGINAIAEN--RTDLVVYGRLFLANPDLPKRFAL-NAPLN
MtOPR05-5 GTFMVAG--GYDRHDGINAIAEN--RADLVVYGRLFLANPDLPKRFAL-DAPLN
MtOPR05-6 GTFMVAG--GYDRHDGINAIAEN--RADLVVYGRLFLANPDLPKRFAL-DAPLN
VI
PsOPR3 GSFLVAG--GYDREDGNAAIASG--KADLVVYGRIFLANPDLPRRFEL-NASLN
PsOPR2 GSFLVAG--GYGREDGNEAISSG--KADLVVYGRIFLANPDLPKRFEL-NAPLN
PsOPR1 GSFLVAG--GYGREDGNEAISSD--KADLVVYGRIFLANPDLPKRFEL-NAPLN
PpOPR4 GTFFIGA--SAGAEEGHEAVRNG--KADMVVYGRLFLANPDLVKRFAL-NAPLN
PpOPR5 GNFLVAG--GYSSEDANEAIRSG--KADMVVFGRLFLANPDLPKRIAL-NAPLN
PpOPR2 GNLIVAG--GFTQKSGNEAIESG--KADAVAYGRVFLANPDLPKRFSV-HAPLN
PpOPR1 GSFICAG--GFNRKEGNDAIRTG--RADAVAFGRHFLANPDLPKRLAS-CAPLN
SmOPR1 GKVLIAG--GYNREDGNEALRAE--KASMVVYGRHFLANPDLVKRFKL-KAPLN
SmOPR2 GTMLVAG--GYGRDDGNEAITSG--KTDLVVYGRHFLANPDLPKRFEL-NSALN
SmOPR3 GAFLAAA--GFDRDSGNAAIESG--LADLVVYARHFLANPDLPRRFQL-NAPLN
SmOPR4 GAFLAAA--GFDRDSGNAAIETG--LADLVVYARHFLANPDLPRRFQL-NAPLN
SmOPR6 GAFLAAA--GFDRDSGNAAIETG--LADLVVYARHFLANPDLPRRFQL-NAPLN
II
PpOPR3 GVFMCTG--GYTREEGMKAIRTG--YTDLISYGRLFLANPDLPSRFYF-DLELN
SmOPR5 IAFLCSG--GFTKELGNAAIQSG--YADLVAYGRLFISNPDLPARFAR-HGDVQ
PpOPR6 -PLLVSG--GHTLDTGNEAIGSG--YADLVGYGRTFIANPDLVKRFAK-DAQLN
PtOPR4 GTFICSG--GFTRELGTQAVAEG--DADLVSYGRDFISNPDLVLRLKL-NAPLN
PtOPR5 GTFMCSG--GFTRELGIQAVTEG--DADLVSYGRLFISNPDLVLRLKL-NAPLN
AtOPR02-1 GTFMSSG--GFNKELGMQAVQQG--DADLVSYGRLFIANPDLVSRFKI-DGELN
OsOPR08-1 GTFMCSG--GYTRELGLEAVESG--DADLVSYGRLFISNPDLVERFRL-NAGLN
ZmOPR01-1 GTFMCSG--GYTRELGVEAIESG--DADLVSYGRLFIANPDLVERFRR-DAPLN
SbOPR07-1 GTFMCSG--GYTRELGVEAVESG--DADLVSYGRLFIANPDLVERFRR-DAPLN
ZmOPR04-1 GTFMCSG--GYTRELGVEAVESW--DADLVSYGRLFIANPDLVERFRR-DAPLN
VII
VcOPR1 GTFITAG--GFKADTGAAAVTSG--QADLVAYGRWYLANPDLHKRFLL-NAPLN
CrOPR2 GTFIVAG--GYKAENGAPAVANG--HADLVAYGRWYLANPDFHKRILL-GAPLN
CrOPR3 GTFMAAG--GFRPGSGAQAVASG--HCDLVAYGRWYLANPDFHKRVLL-GAPLN
CrOPR1 GTVMANG--GISIEAGEELVKSG--TADLVAYGVPFIANANLPALVAA-GVKTA
VcOPR2 GTLAING--GLGIEQGEQYVTDG--VADVVLYGVHFIANANLPELVAG-GVKTG
ScOYE1 GPVIRAGNFALHPEVVREEVKDK---RTLIGYGRFFISNPDLVDRLEK-GLPLN
β7
α7
β8
αB
α8

Additional file 3

Amino acid sequences alignment of Oxidored_FMN domain in OPR genes from eleven species representing six major lineages within the green plants: the green algae (*Chlamydomonas reinhardtii* and *Volvox carteri*), the mosses (*Physcomitrella patens*), the lycophytes (*Selaginella moellendorffii*), the gymnosperms (*Picea sitchensis*), the monocotyledoneous angiosperms (*Oryza sativa*, *Sorghum bicolor* and *Zea mays*) and the dicotyledoneous angiosperms (*Arabidopsis thaliana*, *Populus trichocarpa* and *Medicago truncatula*). Only 3 of 74 OPR genes (AtOPR01-2/3 and SbOPR06-4) without complete Oxidored_FMN domain are excluded, while the outgroup ScOYE1 from yeast (Saito et al., 1991) is included in the alignment. The blue blocks below the alignment represent the fragments selected by the Gblocks program (Castresana, 2000; Talavera and Castresana, 2007) with default parameters underlined. Positions of the alignments where more than 50% of the sequences are identical are shown with black boxes. The secondary structure elements are represented at the bottom of the alignment as tubes or arrows shown in gray based on structural information from OPR proteins AtOPR1 (Fox et al., 2005), AtOPR3 (Malone et al., 2005) and LeOPR3 (Breithaupt et al., 2006). The intron positions of putative ancestral OPR are represented at the top of the alignment as red vertical arrows, and I_1~I_{12} represent intron 1~12. The seven conserved OPR subfamilies are represented as Ⅰ, Ⅱ, Ⅲ, Ⅳ, Ⅴ, Ⅵ and Ⅶ at the left side of the alignment.

References

Breithaupt C, Kurzbauer R, Lilie H, Schaller A, Strassner J, Huber R, Macheroux P, Clausen T (2006) Crystal structure of 12-oxophytodienoate reductase 3 from tomato: self-inhibition by dimerization. Proc Natl Acad Sci U S A 103: 14337-14342

Castresana J (2000) Selection of conserved blocks from multiple alignments for their use in phylogenetic analysis. Mol Biol Evol 17: 540-552

Fox BG, Malone TE, Johnson KA, Madson SE, Aceti D, Bingman CA, Blommel PG, Buchan B, Burns B, Cao J, Cornilescu C, Doreleijers J, Ellefson J, Frederick R, Geetha H, Hruby D, Jeon WB, Kimball T, Kunert J, Markley JL, Newman C, Olson A, Peterson FC, Phillips GN, Jr., Primm J, Ramirez B, Rosenberg NS, Runnels M, Seder K, Shaw J, Smith DW, Sreenath H, Song J, Sussman MR, Thao S, Troestler D, Tyler E, Tyler R, Ulrich E, Vinarov D, Vojtik F, Volkman BF, Wesenberg G, Wrobel RL, Zhang J, Zhao Q, Zolnai Z (2005) X-ray structure of Arabidopsis At1g77680, 12-oxophytodienoate reductase isoform 1. Proteins 61: 206-208

Malone TE, Madson SE, Wrobel RL, Jeon WB, Rosenberg NS, Johnson KA, Bingman CA, Smith DW, Phillips GN, Jr., Markley JL, Fox BG (2005) X-ray structure of Arabidopsis At2g06050, 12-oxophytodienoate reductase isoform 3. Proteins 58: 243-245

Saito K, Thiele DJ, Davio M, Lockridge O, Massey V (1991) The cloning and expression of a gene encoding Old Yellow Enzyme from Saccharomyces carlsbergensis. J Biol Chem 266: 20720-20724

Talavera G, Castresana J (2007) Improvement of phylogenies after removing divergent and ambiguously aligned blocks from protein sequence alignments. Syst Biol 56: 564-577
