# Supplementary material for: Phylogenetic analysis, structural evolution and functional divergence of the 12-oxo-phytodienoate acid reductase gene family in plants
Source: BMC Evol Biol. 2009 May 5;9:90. doi: 10.1186/1471-2148-9-90 (PMC2688005; doi:10.1186/1471-2148-9-90)
Supplement: Additional file 4 — Nucleotide sequences alignment of OPR family genes from eleven species. Nucleotide sequences alignment of OPR genes from eleven species representing the six major lineages within the green plants: the green algae (Chlamydomonas reinhardtii and Volvox carteri), the mosses (Physcomitrella patens), the lycophytes (Selaginella moellendorffii), the gymnosperms (Picea sitchensis), the monocotyledoneous angiosperms (Oryza sativa, Sorghum bicolor and Zea mays) and the dicotyledoneous angiosperms (Arabidopsis thaliana, Populus trichocarpa and Medicago truncatula). Identical residues in the alignments are shaded and seven well-conserved OPR subfamilies are represented as Sub.I, II, III, IV, V, VI and VII at the left side of the alignment. [file 1471-2148-9-90-S4.pdf]

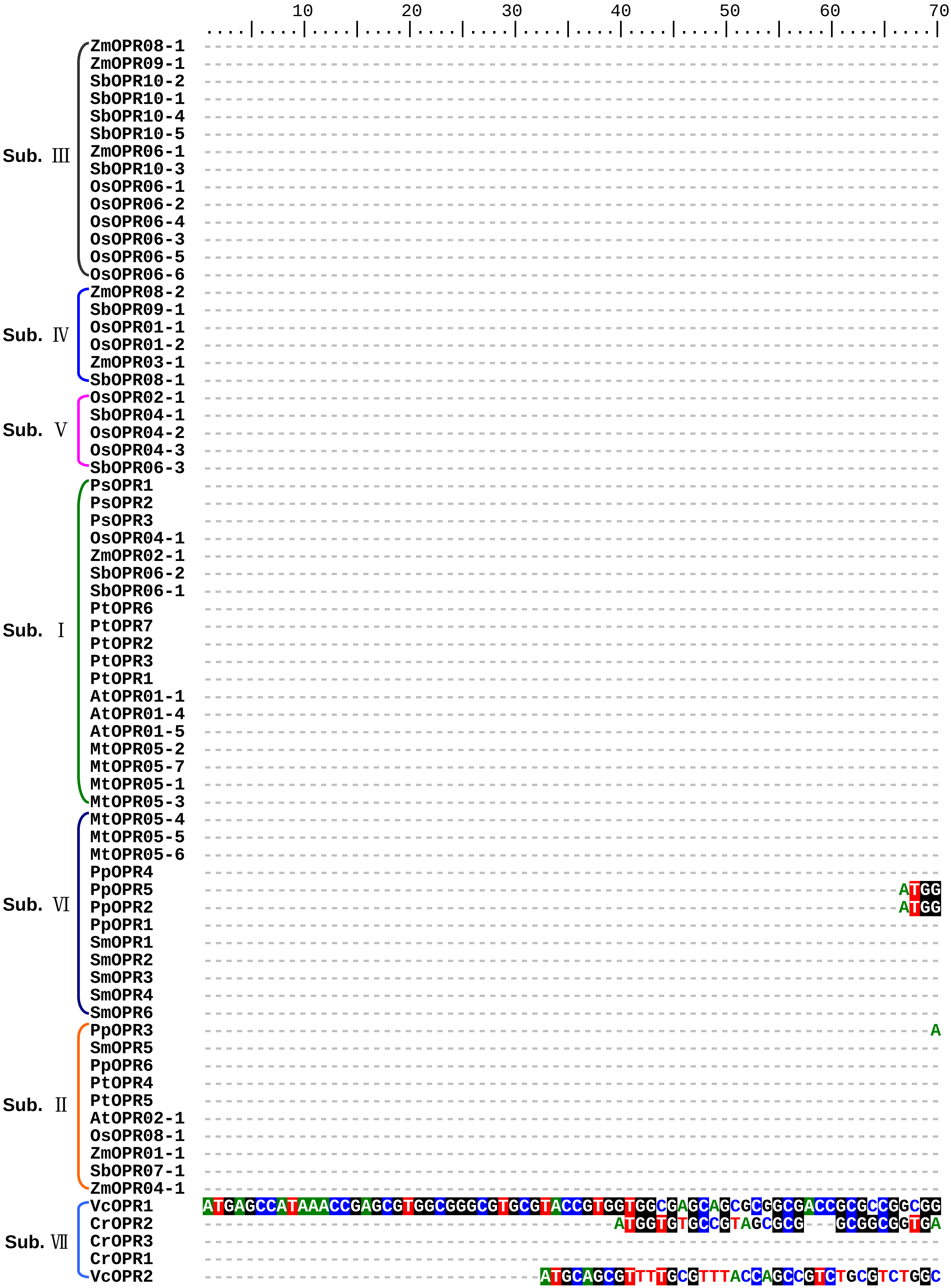

10 20 30 40 50 60 70
Sub. III
ZmOPR08-1
ZmOPR09-1
SbOPR10-2
SbOPR10-1
SbOPR10-4
SbOPR10-5
ZmOPR06-1
SbOPR10-3
OsOPR06-1
OsOPR06-2
OsOPR06-4
OsOPR06-3
OsOPR06-5
OsOPR06-6
Sub. IV
ZmOPR08-2
SbOPR09-1
OsOPR01-1
OsOPR01-2
ZmOPR03-1
SbOPR08-1
Sub. V
OsOPR02-1
SbOPR04-1
OsOPR04-2
OsOPR04-3
SbOPR06-3
Sub. I
PsOPR1
PsOPR2
PsOPR3
OsOPR04-1
ZmOPR02-1
SbOPR06-2
SbOPR06-1
PtOPR6
PtOPR7
PtOPR2
PtOPR3
PtOPR1
AtOPR01-1
AtOPR01-4
AtOPR01-5
MtOPR05-2
MtOPR05-7
MtOPR05-1
MtOPR05-3
Sub. VI
MtOPR05-4
MtOPR05-5
MtOPR05-6
PpOPR4
PpOPR5 ATGG
PpOPR2 ATGG
PpOPR1
SmOPR1
SmOPR2
SmOPR3
SmOPR4
SmOPR6
Sub. II
PpOPR3 A
SmOPR5
PpOPR6
PtOPR4
PtOPR5
AtOPR02-1
OsOPR08-1
ZmOPR01-1
SbOPR07-1
ZmOPR04-1
Sub. VII
VcOPR1 ATGAGCCATAAACCGAGCGTGGCGGGCGTGCGTACCGTGGTGGCGAGCAGCGCGGCGACCGCGCCGGCGG
CrOPR2 ATGGTGTGCCGTAGCGCG GCGGCGGTGA
CrOPR3
CrOPR1
VcOPR2 ATGCAGCGTTTTGCGTTTACCAGCCGTCTGCGTCTGGC

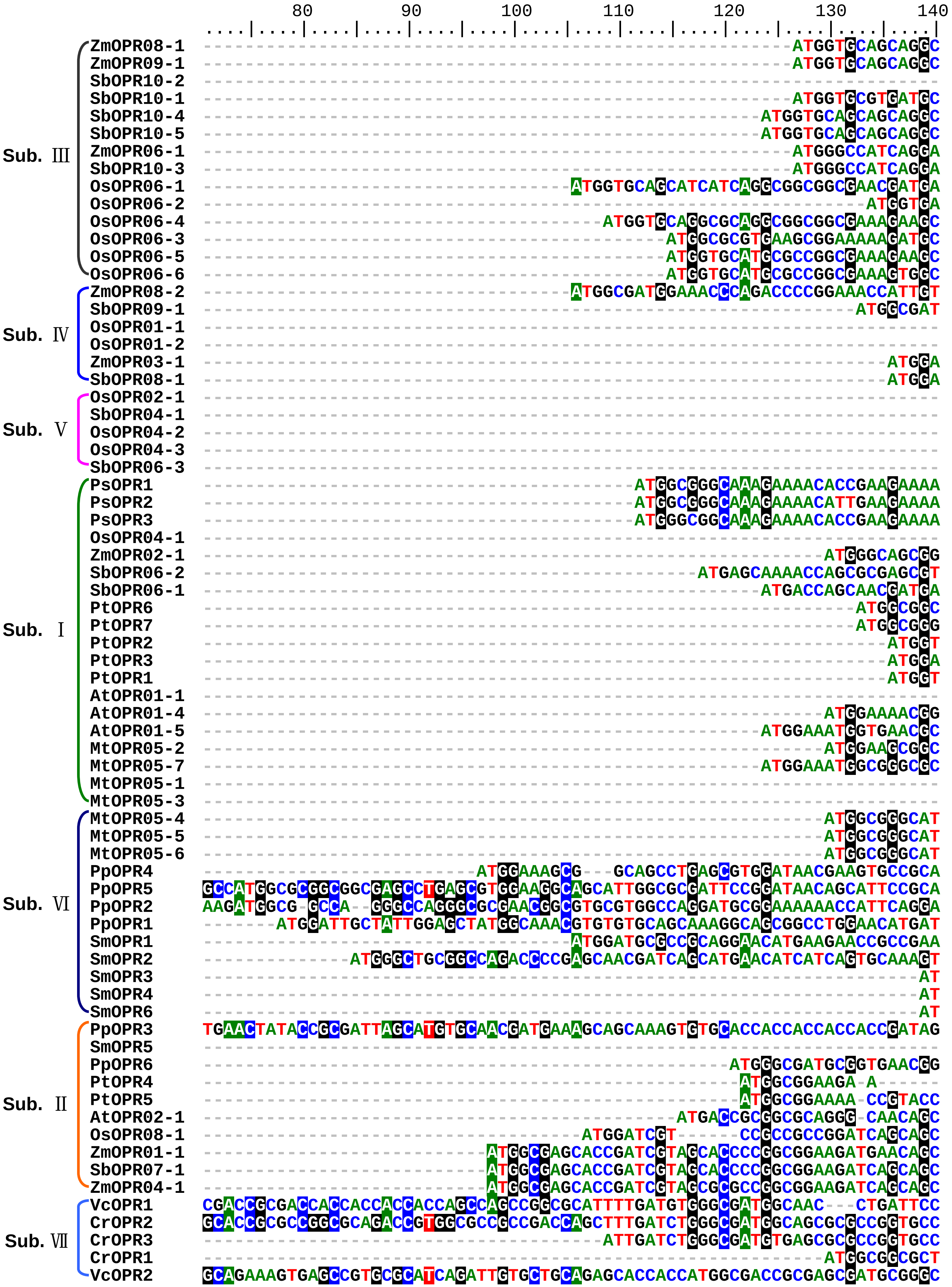

80
90
100
110
120
130
140
Sub. Ⅲ
ZmOPR08-1
ZmOPR09-1
SbOPR10-2
SbOPR10-1
SbOPR10-4
SbOPR10-5
ZmOPR06-1
SbOPR10-3
OsOPR06-1
OsOPR06-2
OsOPR06-4
OsOPR06-3
OsOPR06-5
OsOPR06-6
Sub. Ⅳ
ZmOPR08-2
SbOPR09-1
OsOPR01-1
OsOPR01-2
ZmOPR03-1
SbOPR08-1
Sub. Ⅴ
OsOPR02-1
SbOPR04-1
OsOPR04-2
OsOPR04-3
SbOPR06-3
Sub. Ⅰ
PsOPR1
PsOPR2
PsOPR3
OsOPR04-1
ZmOPR02-1
SbOPR06-2
SbOPR06-1
PtOPR6
PtOPR7
PtOPR2
PtOPR3
PtOPR1
AtOPR01-1
AtOPR01-4
AtOPR01-5
MtOPR05-2
MtOPR05-7
MtOPR05-1
MtOPR05-3
Sub. Ⅵ
MtOPR05-4
MtOPR05-5
MtOPR05-6
PpOPR4
PpOPR5
PpOPR2
PpOPR1
SmOPR1
SmOPR2
SmOPR3
SmOPR4
SmOPR6
Sub. Ⅱ
PpOPR3
SmOPR5
PpOPR6
PtOPR4
PtOPR5
AtOPR02-1
OsOPR08-1
ZmOPR01-1
SbOPR07-1
ZmOPR04-1
Sub. Ⅶ
VcOPR1
CrOPR2
CrOPR3
CrOPR1
VcOPR2

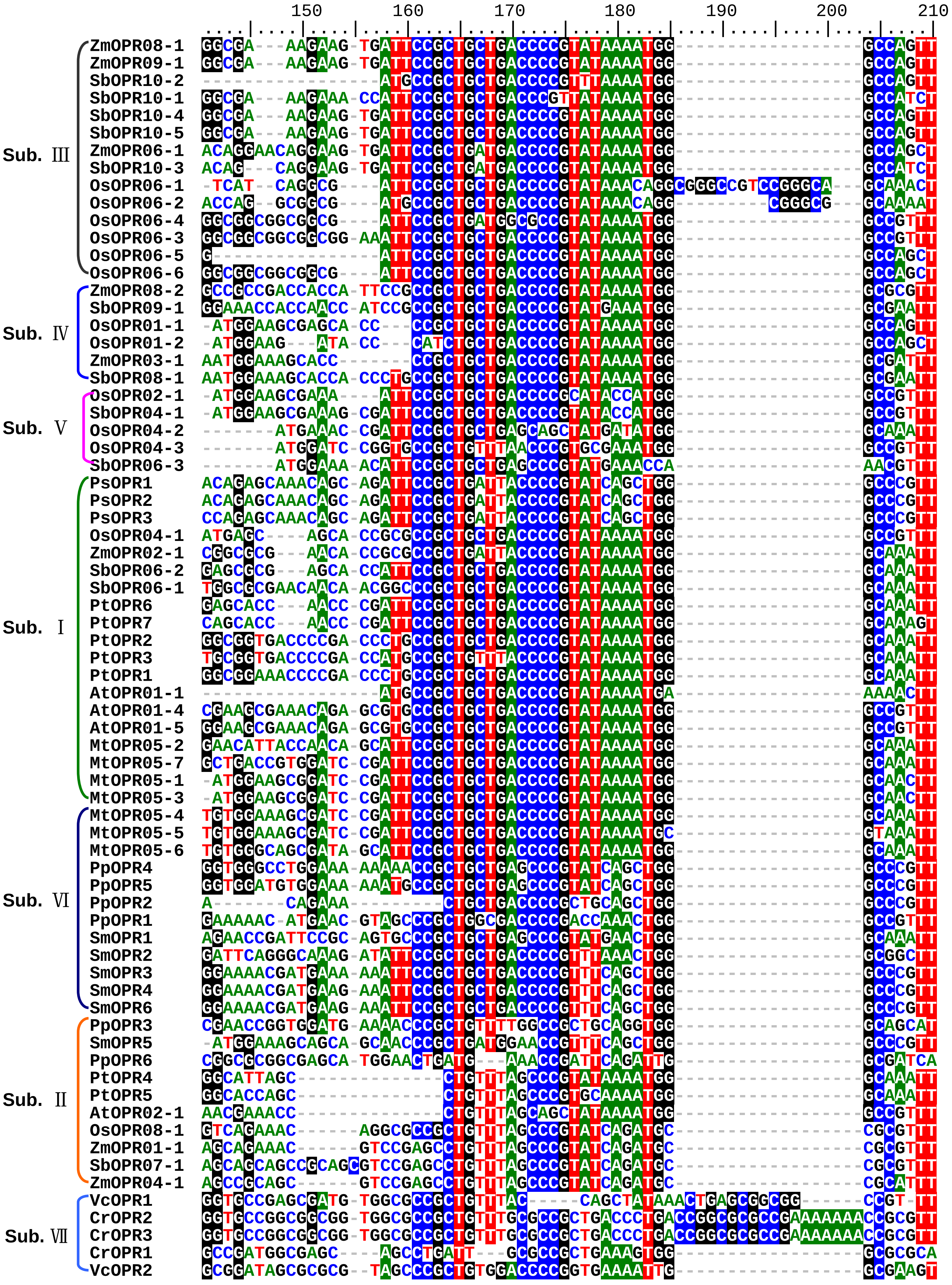
150
160
170
180
190
200
210
Sub. Ⅲ
ZmOPR08-1
ZmOPR09-1
SbOPR10-2
SbOPR10-1
SbOPR10-4
SbOPR10-5
ZmOPR06-1
SbOPR10-3
OsOPR06-1
OsOPR06-2
OsOPR06-4
OsOPR06-3
OsOPR06-5
OsOPR06-6
Sub. Ⅳ
ZmOPR08-2
SbOPR09-1
OsOPR01-1
OsOPR01-2
ZmOPR03-1
SbOPR08-1
Sub. Ⅴ
OsOPR02-1
SbOPR04-1
OsOPR04-2
OsOPR04-3
SbOPR06-3
Sub. Ⅰ
PsOPR1
PsOPR2
PsOPR3
OsOPR04-1
ZmOPR02-1
SbOPR06-2
SbOPR06-1
PtOPR6
PtOPR7
PtOPR2
PtOPR3
PtOPR1
AtOPR01-1
AtOPR01-4
AtOPR01-5
MtOPR05-2
MtOPR05-7
MtOPR05-1
MtOPR05-3
Sub. Ⅵ
MtOPR05-4
MtOPR05-5
MtOPR05-6
PpOPR4
PpOPR5
PpOPR2
PpOPR1
SmOPR1
SmOPR2
SmOPR3
SmOPR4
SmOPR6
Sub. Ⅱ
PpOPR3
SmOPR5
PpOPR6
PtOPR4
PtOPR5
AtOPR02-1
OsOPR08-1
ZmOPR01-1
SbOPR07-1
ZmOPR04-1
Sub. Ⅶ
VcOPR1
CrOPR2
CrOPR3
CrOPR1
VcOPR2

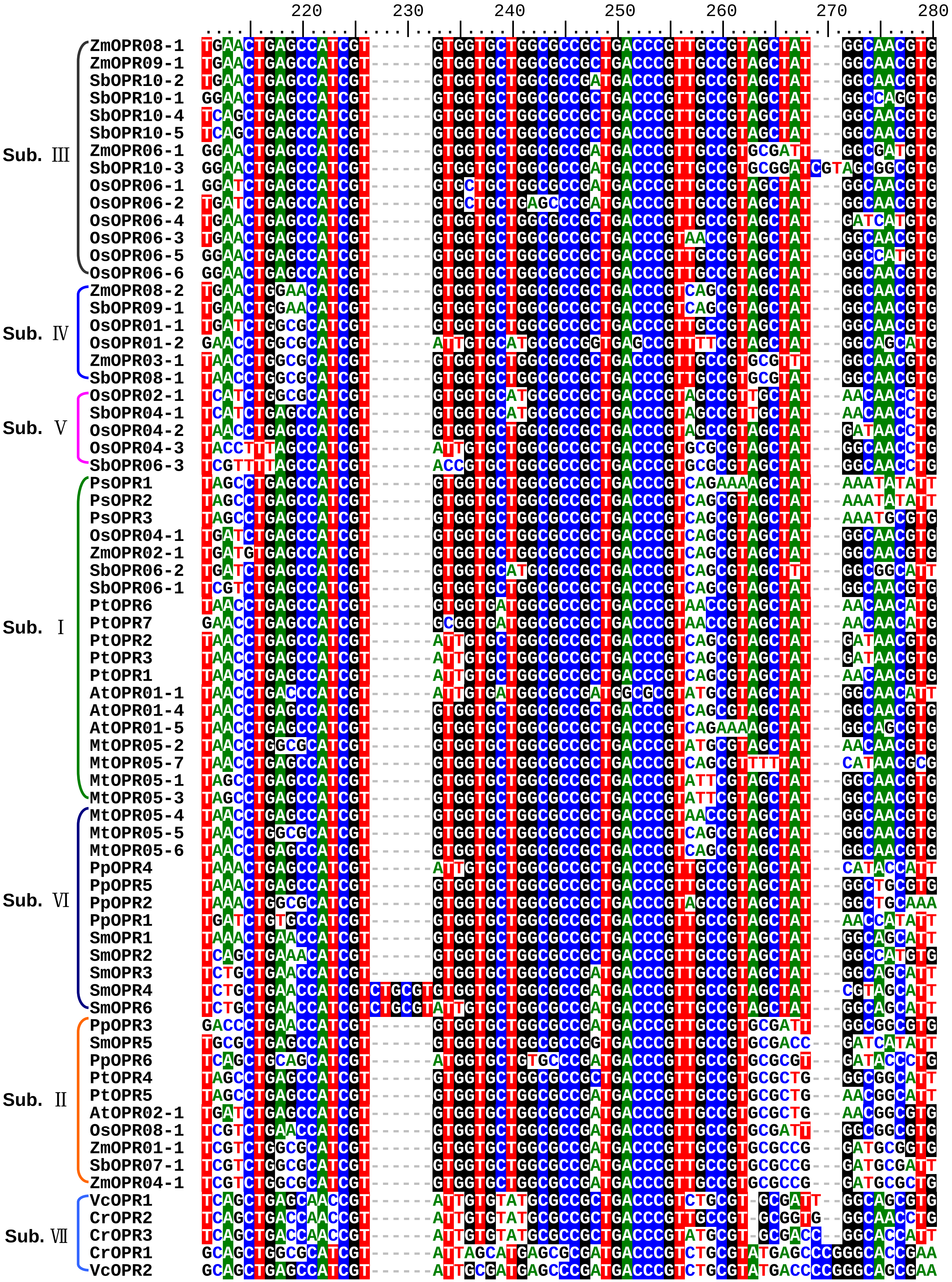

220
230
240
250
260
270
280
Sub. Ⅲ
ZmOPR08-1
ZmOPR09-1
SbOPR10-2
SbOPR10-1
SbOPR10-4
SbOPR10-5
ZmOPR06-1
SbOPR10-3
OsOPR06-1
OsOPR06-2
OsOPR06-4
OsOPR06-3
OsOPR06-5
OsOPR06-6
Sub. Ⅳ
ZmOPR08-2
SbOPR09-1
OsOPR01-1
OsOPR01-2
ZmOPR03-1
SbOPR08-1
Sub. Ⅴ
OsOPR02-1
SbOPR04-1
OsOPR04-2
OsOPR04-3
SbOPR06-3
Sub. Ⅰ
PsOPR1
PsOPR2
PsOPR3
OsOPR04-1
ZmOPR02-1
SbOPR06-2
SbOPR06-1
PtOPR6
PtOPR7
PtOPR2
PtOPR3
PtOPR1
AtOPR01-1
AtOPR01-4
AtOPR01-5
MtOPR05-2
MtOPR05-7
MtOPR05-1
MtOPR05-3
Sub. Ⅵ
MtOPR05-4
MtOPR05-5
MtOPR05-6
PpOPR4
PpOPR5
PpOPR2
PpOPR1
SmOPR1
SmOPR2
SmOPR3
SmOPR4
SmOPR6
Sub. Ⅱ
PpOPR3
SmOPR5
PpOPR6
PtOPR4
PtOPR5
AtOPR02-1
OsOPR08-1
ZmOPR01-1
SbOPR07-1
ZmOPR04-1
Sub. Ⅶ
VcOPR1
CrOPR2
CrOPR3
CrOPR1
VcOPR2

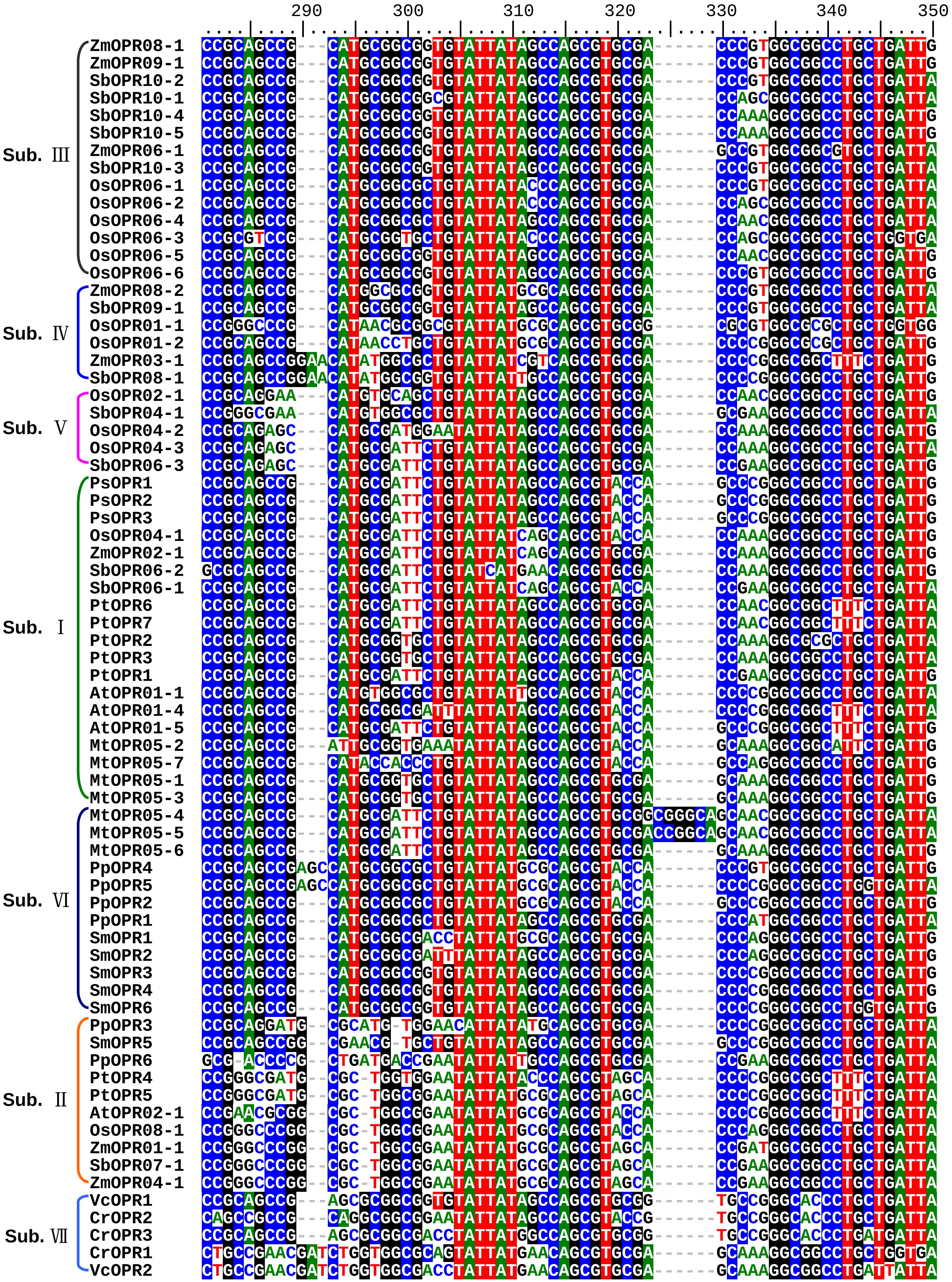
290
300
310
320
330
340
350
Sub. Ⅲ
ZmOPR08-1
ZmOPR09-1
SbOPR10-2
SbOPR10-1
SbOPR10-4
SbOPR10-5
ZmOPR06-1
SbOPR10-3
OsOPR06-1
OsOPR06-2
OsOPR06-4
OsOPR06-3
OsOPR06-5
OsOPR06-6
Sub. Ⅳ
ZmOPR08-2
SbOPR09-1
OsOPR01-1
OsOPR01-2
ZmOPR03-1
SbOPR08-1
Sub. Ⅴ
OsOPR02-1
SbOPR04-1
OsOPR04-2
OsOPR04-3
SbOPR06-3
Sub. Ⅰ
PsOPR1
PsOPR2
PsOPR3
OsOPR04-1
ZmOPR02-1
SbOPR06-2
SbOPR06-1
PtOPR6
PtOPR7
PtOPR2
PtOPR3
PtOPR1
AtOPR01-1
AtOPR01-4
AtOPR01-5
MtOPR05-2
MtOPR05-7
MtOPR05-1
MtOPR05-3
Sub. Ⅵ
MtOPR05-4
MtOPR05-5
MtOPR05-6
PpOPR4
PpOPR5
PpOPR2
PpOPR1
SmOPR1
SmOPR2
SmOPR3
SmOPR4
SmOPR6
Sub. Ⅱ
PpOPR3
SmOPR5
PpOPR6
PtOPR4
PtOPR5
AtOPR02-1
OsOPR08-1
ZmOPR01-1
SbOPR07-1
ZmOPR04-1
Sub. Ⅶ
VcOPR1
CrOPR2
CrOPR3
CrOPR1
VcOPR2

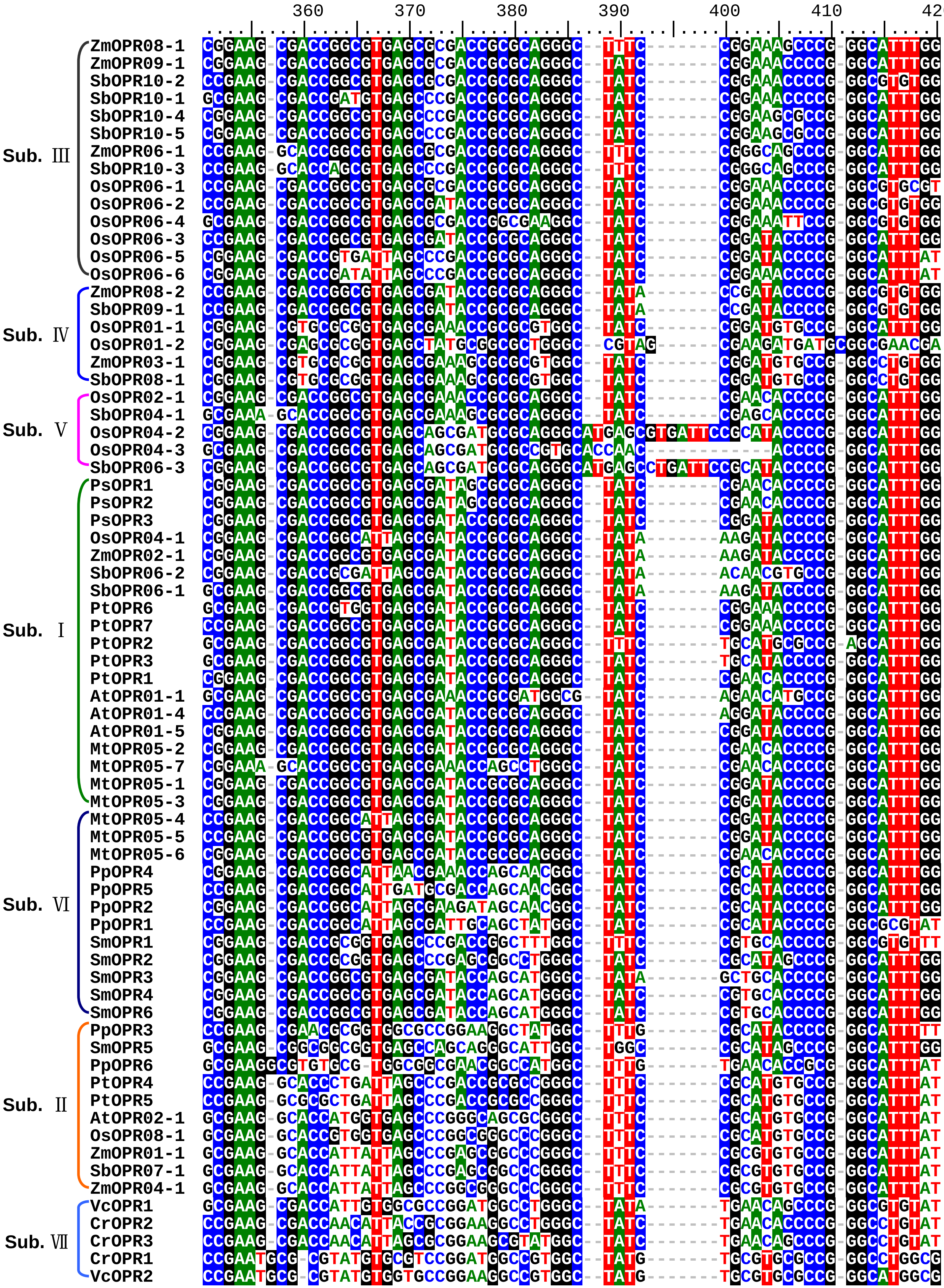
360
370
380
390
400
410
420
Sub. Ⅲ
ZmOPR08-1
ZmOPR09-1
SbOPR10-2
SbOPR10-1
SbOPR10-4
SbOPR10-5
ZmOPR06-1
SbOPR10-3
OsOPR06-1
OsOPR06-2
OsOPR06-4
OsOPR06-3
OsOPR06-5
OsOPR06-6
Sub. Ⅳ
ZmOPR08-2
SbOPR09-1
OsOPR01-1
OsOPR01-2
ZmOPR03-1
SbOPR08-1
Sub. Ⅴ
OsOPR02-1
SbOPR04-1
OsOPR04-2
OsOPR04-3
SbOPR06-3
Sub. Ⅰ
PsOPR1
PsOPR2
PsOPR3
OsOPR04-1
ZmOPR02-1
SbOPR06-2
SbOPR06-1
PtOPR6
PtOPR7
PtOPR2
PtOPR3
PtOPR1
AtOPR01-1
AtOPR01-4
AtOPR01-5
MtOPR05-2
MtOPR05-7
MtOPR05-1
MtOPR05-3
Sub. Ⅵ
MtOPR05-4
MtOPR05-5
MtOPR05-6
PpOPR4
PpOPR5
PpOPR2
PpOPR1
SmOPR1
SmOPR2
SmOPR3
SmOPR4
SmOPR6
Sub. Ⅱ
PpOPR3
SmOPR5
PpOPR6
PtOPR4
PtOPR5
AtOPR02-1
OsOPR08-1
ZmOPR01-1
SbOPR07-1
ZmOPR04-1
Sub. Ⅶ
VcOPR1
CrOPR2
CrOPR3
CrOPR1
VcOPR2

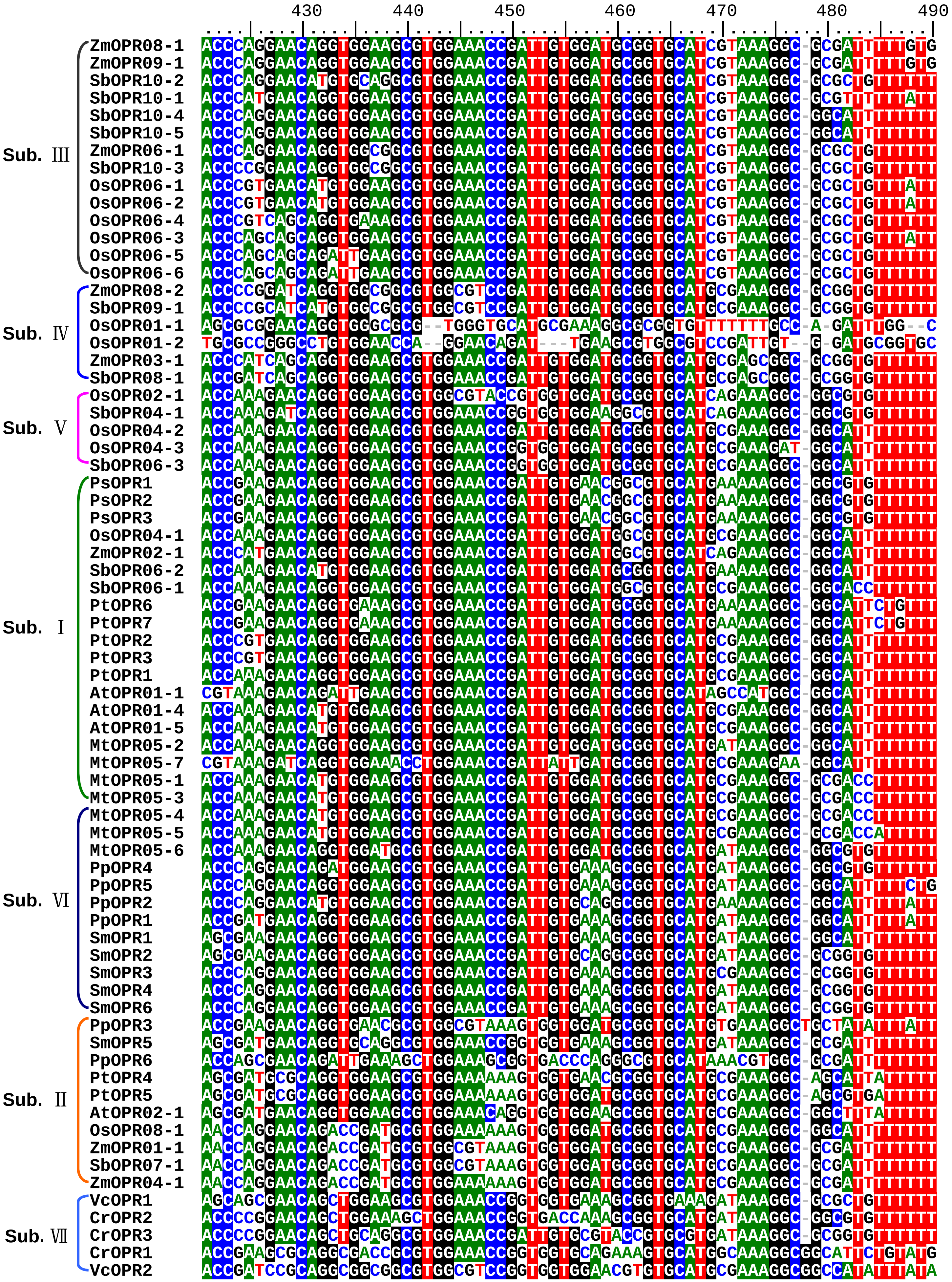

430
440
450
460
470
480
490
Sub. Ⅲ
ZmOPR08-1
ZmOPR09-1
SbOPR10-2
SbOPR10-1
SbOPR10-4
SbOPR10-5
ZmOPR06-1
SbOPR10-3
OsOPR06-1
OsOPR06-2
OsOPR06-4
OsOPR06-3
OsOPR06-5
OsOPR06-6
Sub. Ⅳ
ZmOPR08-2
SbOPR09-1
OsOPR01-1
OsOPR01-2
ZmOPR03-1
SbOPR08-1
Sub. Ⅴ
OsOPR02-1
SbOPR04-1
OsOPR04-2
OsOPR04-3
SbOPR06-3
Sub. Ⅰ
PsOPR1
PsOPR2
PsOPR3
OsOPR04-1
ZmOPR02-1
SbOPR06-2
SbOPR06-1
PtOPR6
PtOPR7
PtOPR2
PtOPR3
PtOPR1
AtOPR01-1
AtOPR01-4
AtOPR01-5
MtOPR05-2
MtOPR05-7
MtOPR05-1
MtOPR05-3
Sub. Ⅵ
MtOPR05-4
MtOPR05-5
MtOPR05-6
PpOPR4
PpOPR5
PpOPR2
PpOPR1
SmOPR1
SmOPR2
SmOPR3
SmOPR4
SmOPR6
Sub. Ⅱ
PpOPR3
SmOPR5
PpOPR6
PtOPR4
PtOPR5
AtOPR02-1
OsOPR08-1
ZmOPR01-1
SbOPR07-1
ZmOPR04-1
Sub. Ⅶ
VcOPR1
CrOPR2
CrOPR3
CrOPR1
VcOPR2

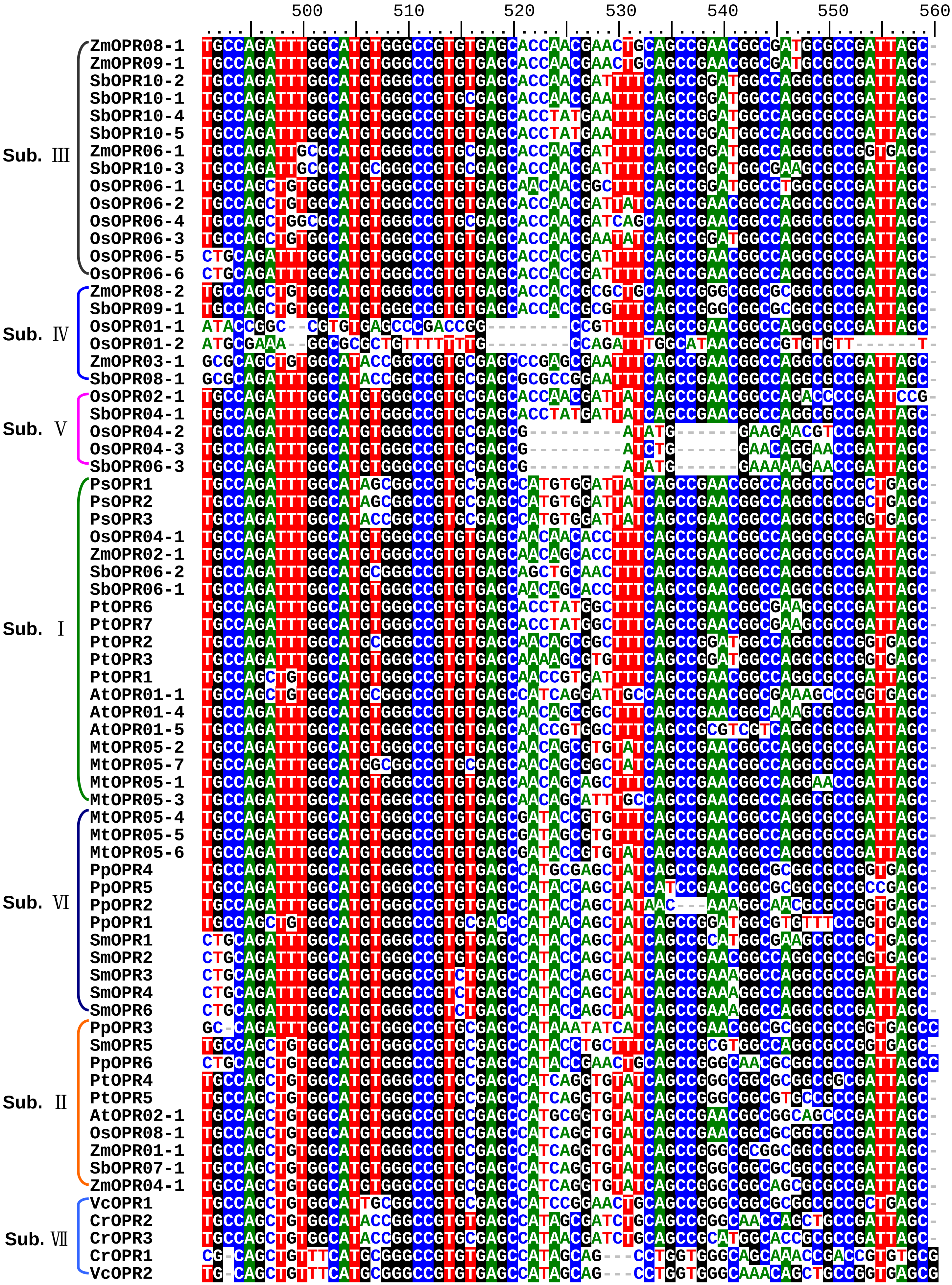
500
510
520
530
540
550
560
Sub. Ⅲ
ZmOPR08-1
ZmOPR09-1
SbOPR10-2
SbOPR10-1
SbOPR10-4
SbOPR10-5
ZmOPR06-1
SbOPR10-3
OsOPR06-1
OsOPR06-2
OsOPR06-4
OsOPR06-3
OsOPR06-5
OsOPR06-6
Sub. Ⅳ
ZmOPR08-2
SbOPR09-1
OsOPR01-1
OsOPR01-2
ZmOPR03-1
SbOPR08-1
Sub. Ⅴ
OsOPR02-1
SbOPR04-1
OsOPR04-2
OsOPR04-3
SbOPR06-3
Sub. Ⅰ
PsOPR1
PsOPR2
PsOPR3
OsOPR04-1
ZmOPR02-1
SbOPR06-2
SbOPR06-1
PtOPR6
PtOPR7
PtOPR2
PtOPR3
PtOPR1
AtOPR01-1
AtOPR01-4
AtOPR01-5
MtOPR05-2
MtOPR05-7
MtOPR05-1
MtOPR05-3
Sub. Ⅵ
MtOPR05-4
MtOPR05-5
MtOPR05-6
PpOPR4
PpOPR5
PpOPR2
PpOPR1
SmOPR1
SmOPR2
SmOPR3
SmOPR4
SmOPR6
Sub. Ⅱ
PpOPR3
SmOPR5
PpOPR6
PtOPR4
PtOPR5
AtOPR02-1
OsOPR08-1
ZmOPR01-1
SbOPR07-1
ZmOPR04-1
Sub. Ⅶ
VcOPR1
CrOPR2
CrOPR3
CrOPR1
VcOPR2

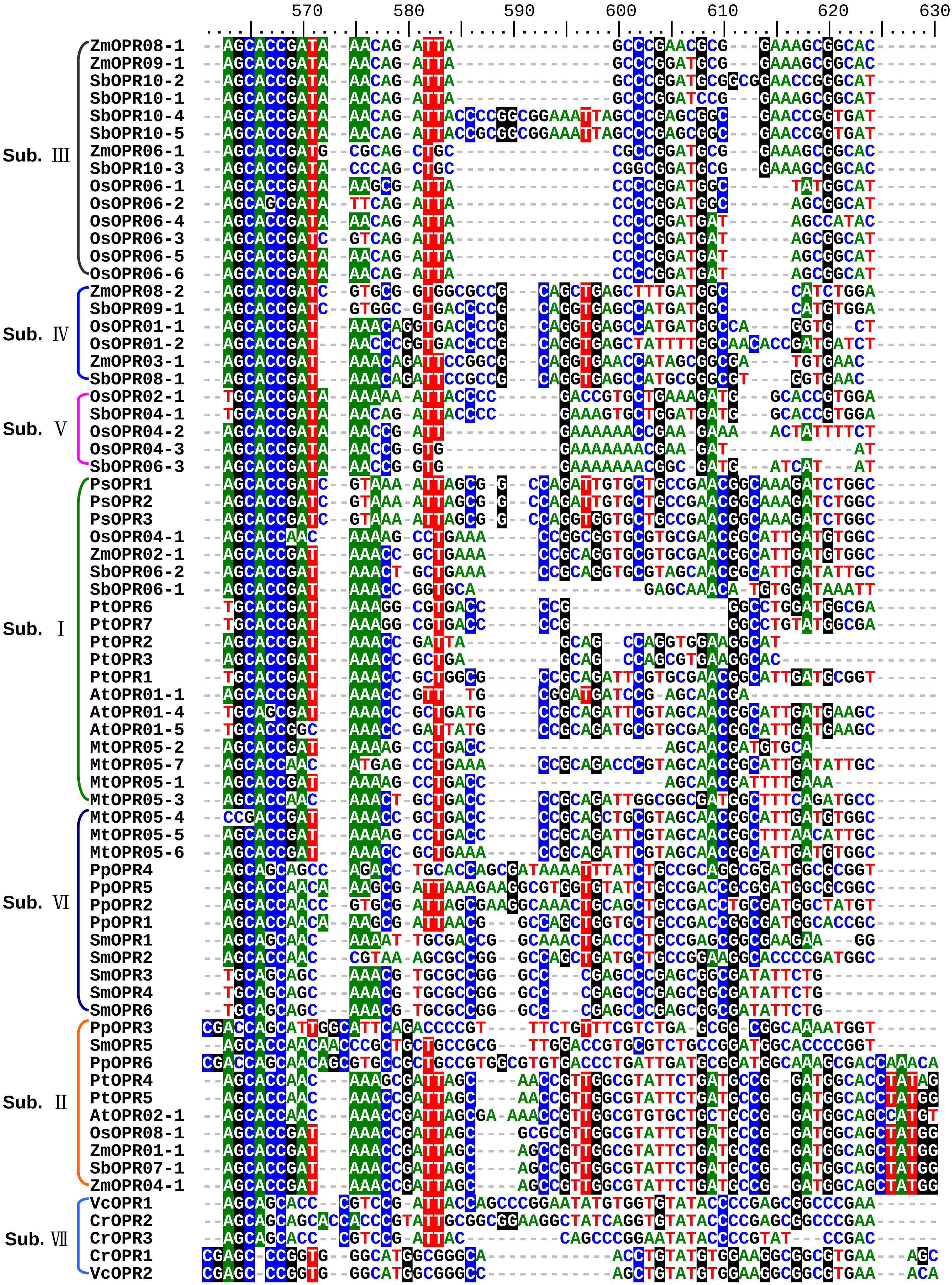

570
580
590
600
610
620
630
Sub. Ⅲ
ZmOPR08-1
ZmOPR09-1
SbOPR10-2
SbOPR10-1
SbOPR10-4
SbOPR10-5
ZmOPR06-1
SbOPR10-3
OsOPR06-1
OsOPR06-2
OsOPR06-4
OsOPR06-3
OsOPR06-5
OsOPR06-6
Sub. Ⅳ
ZmOPR08-2
SbOPR09-1
OsOPR01-1
OsOPR01-2
ZmOPR03-1
SbOPR08-1
Sub. Ⅴ
OsOPR02-1
SbOPR04-1
OsOPR04-2
OsOPR04-3
SbOPR06-3
Sub. Ⅰ
PsOPR1
PsOPR2
PsOPR3
OsOPR04-1
ZmOPR02-1
SbOPR06-2
SbOPR06-1
PtOPR6
PtOPR7
PtOPR2
PtOPR3
PtOPR1
AtOPR01-1
AtOPR01-4
AtOPR01-5
MtOPR05-2
MtOPR05-7
MtOPR05-1
MtOPR05-3
Sub. Ⅵ
MtOPR05-4
MtOPR05-5
MtOPR05-6
PpOPR4
PpOPR5
PpOPR2
PpOPR1
SmOPR1
SmOPR2
SmOPR3
SmOPR4
SmOPR6
Sub. Ⅱ
PpOPR3
SmOPR5
PpOPR6
PtOPR4
PtOPR5
AtOPR02-1
OsOPR08-1
ZmOPR01-1
SbOPR07-1
ZmOPR04-1
Sub. Ⅶ
VcOPR1
CrOPR2
CrOPR3
CrOPR1
VcOPR2

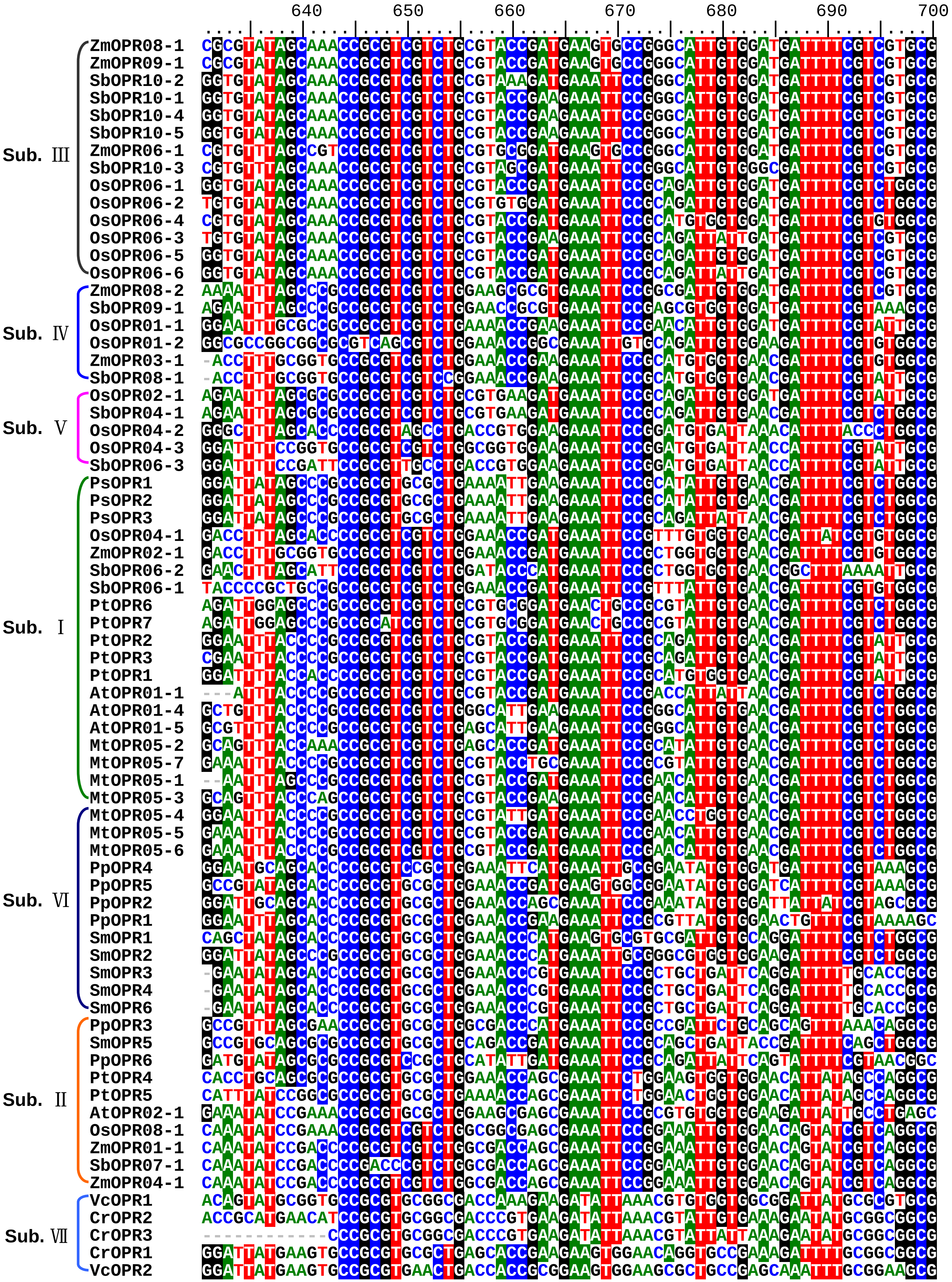
640
650
660
670
680
690
700
Sub. Ⅲ
ZmOPR08-1
ZmOPR09-1
SbOPR10-2
SbOPR10-1
SbOPR10-4
SbOPR10-5
ZmOPR06-1
SbOPR10-3
OsOPR06-1
OsOPR06-2
OsOPR06-4
OsOPR06-3
OsOPR06-5
OsOPR06-6
Sub. Ⅳ
ZmOPR08-2
SbOPR09-1
OsOPR01-1
OsOPR01-2
ZmOPR03-1
SbOPR08-1
Sub. Ⅴ
OsOPR02-1
SbOPR04-1
OsOPR04-2
OsOPR04-3
SbOPR06-3
Sub. Ⅰ
PsOPR1
PsOPR2
PsOPR3
OsOPR04-1
ZmOPR02-1
SbOPR06-2
SbOPR06-1
PtOPR6
PtOPR7
PtOPR2
PtOPR3
PtOPR1
AtOPR01-1
AtOPR01-4
AtOPR01-5
MtOPR05-2
MtOPR05-7
MtOPR05-1
MtOPR05-3
Sub. Ⅵ
MtOPR05-4
MtOPR05-5
MtOPR05-6
PpOPR4
PpOPR5
PpOPR2
PpOPR1
SmOPR1
SmOPR2
SmOPR3
SmOPR4
SmOPR6
Sub. Ⅱ
PpOPR3
SmOPR5
PpOPR6
PtOPR4
PtOPR5
AtOPR02-1
OsOPR08-1
ZmOPR01-1
SbOPR07-1
ZmOPR04-1
Sub. Ⅶ
VcOPR1
CrOPR2
CrOPR3
CrOPR1
VcOPR2

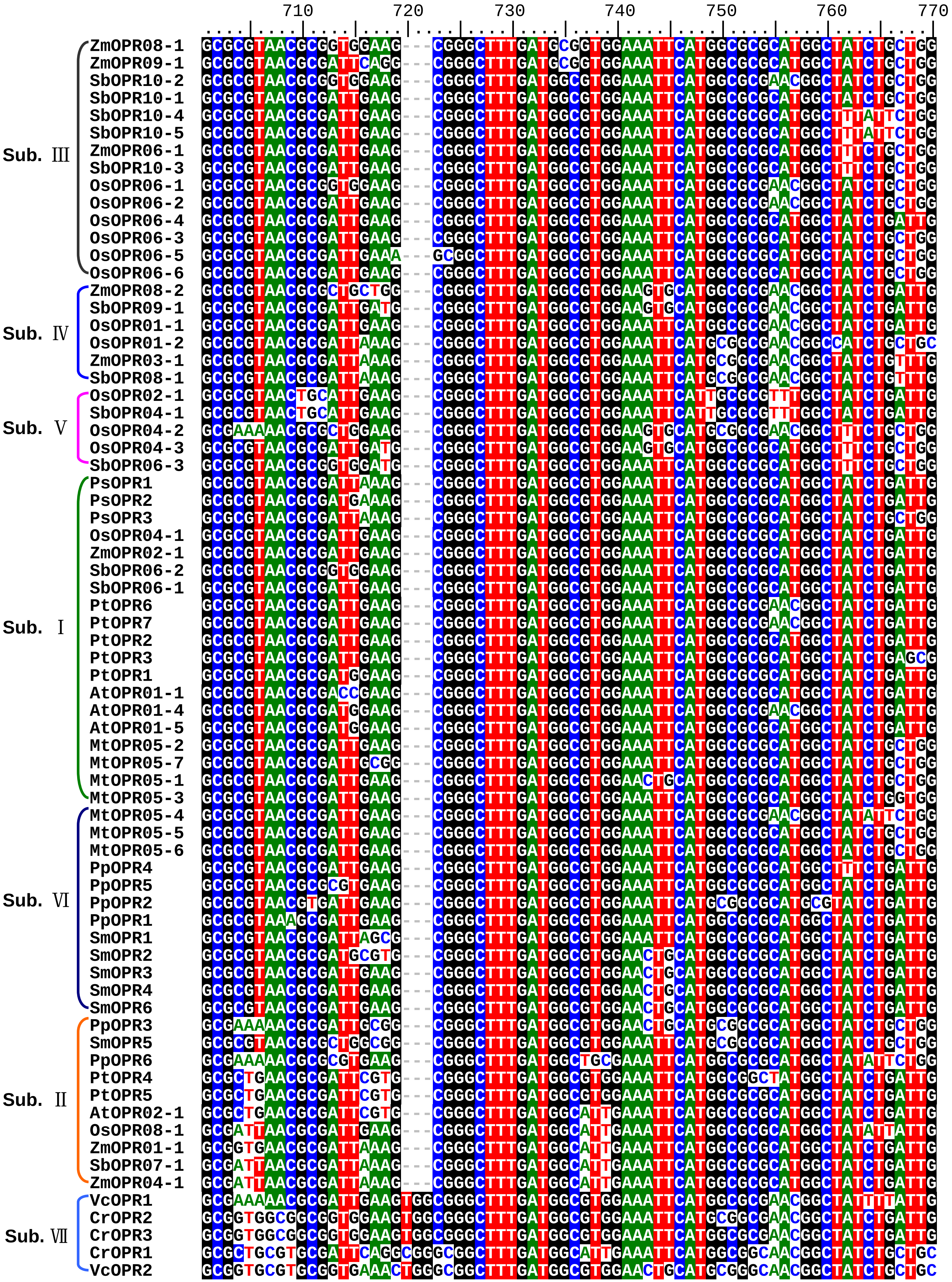

710
720
730
740
750
760
770
Sub. Ⅲ
ZmOPR08-1
ZmOPR09-1
SbOPR10-2
SbOPR10-1
SbOPR10-4
SbOPR10-5
ZmOPR06-1
SbOPR10-3
OsOPR06-1
OsOPR06-2
OsOPR06-4
OsOPR06-3
OsOPR06-5
OsOPR06-6
Sub. Ⅳ
ZmOPR08-2
SbOPR09-1
OsOPR01-1
OsOPR01-2
ZmOPR03-1
SbOPR08-1
Sub. Ⅴ
OsOPR02-1
SbOPR04-1
OsOPR04-2
OsOPR04-3
SbOPR06-3
Sub. Ⅰ
PsOPR1
PsOPR2
PsOPR3
OsOPR04-1
ZmOPR02-1
SbOPR06-2
SbOPR06-1
PtOPR6
PtOPR7
PtOPR2
PtOPR3
PtOPR1
AtOPR01-1
AtOPR01-4
AtOPR01-5
MtOPR05-2
MtOPR05-7
MtOPR05-1
MtOPR05-3
Sub. Ⅵ
MtOPR05-4
MtOPR05-5
MtOPR05-6
PpOPR4
PpOPR5
PpOPR2
PpOPR1
SmOPR1
SmOPR2
SmOPR3
SmOPR4
SmOPR6
Sub. Ⅱ
PpOPR3
SmOPR5
PpOPR6
PtOPR4
PtOPR5
AtOPR02-1
OsOPR08-1
ZmOPR01-1
SbOPR07-1
ZmOPR04-1
Sub. Ⅶ
VcOPR1
CrOPR2
CrOPR3
CrOPR1
VcOPR2

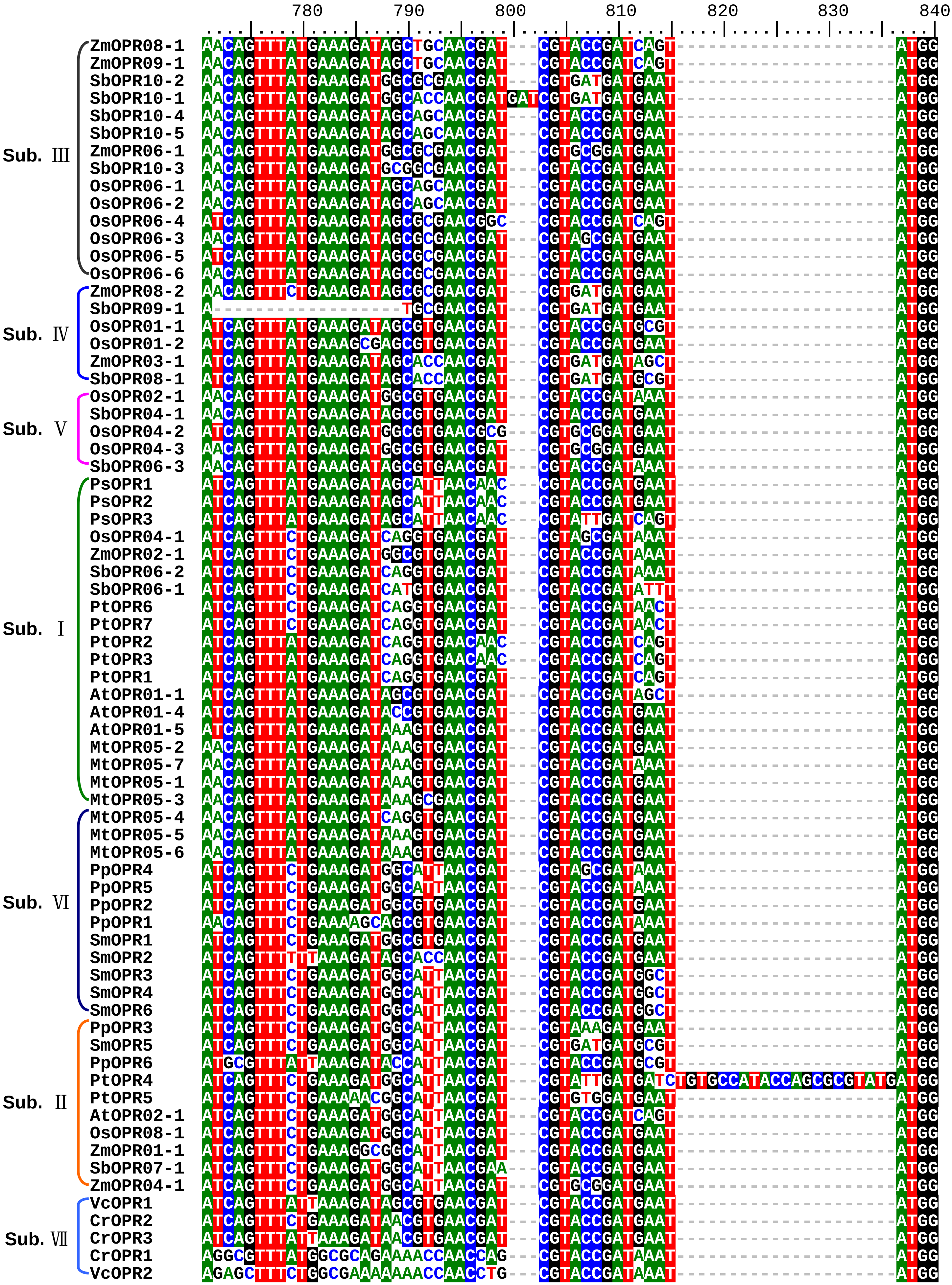
780
790
800
810
820
830
840
Sub. Ⅲ
ZmOPR08-1
ZmOPR09-1
SbOPR10-2
SbOPR10-1
SbOPR10-4
SbOPR10-5
ZmOPR06-1
SbOPR10-3
OsOPR06-1
OsOPR06-2
OsOPR06-4
OsOPR06-3
OsOPR06-5
OsOPR06-6
Sub. Ⅳ
ZmOPR08-2
SbOPR09-1
OsOPR01-1
OsOPR01-2
ZmOPR03-1
SbOPR08-1
Sub. Ⅴ
OsOPR02-1
SbOPR04-1
OsOPR04-2
OsOPR04-3
SbOPR06-3
Sub. Ⅰ
PsOPR1
PsOPR2
PsOPR3
OsOPR04-1
ZmOPR02-1
SbOPR06-2
SbOPR06-1
PtOPR6
PtOPR7
PtOPR2
PtOPR3
PtOPR1
AtOPR01-1
AtOPR01-4
AtOPR01-5
MtOPR05-2
MtOPR05-7
MtOPR05-1
MtOPR05-3
Sub. Ⅵ
MtOPR05-4
MtOPR05-5
MtOPR05-6
PpOPR4
PpOPR5
PpOPR2
PpOPR1
SmOPR1
SmOPR2
SmOPR3
SmOPR4
SmOPR6
Sub. Ⅱ
PpOPR3
SmOPR5
PpOPR6
PtOPR4
PtOPR5
AtOPR02-1
OsOPR08-1
ZmOPR01-1
SbOPR07-1
ZmOPR04-1
Sub. Ⅶ
VcOPR1
CrOPR2
CrOPR3
CrOPR1
VcOPR2

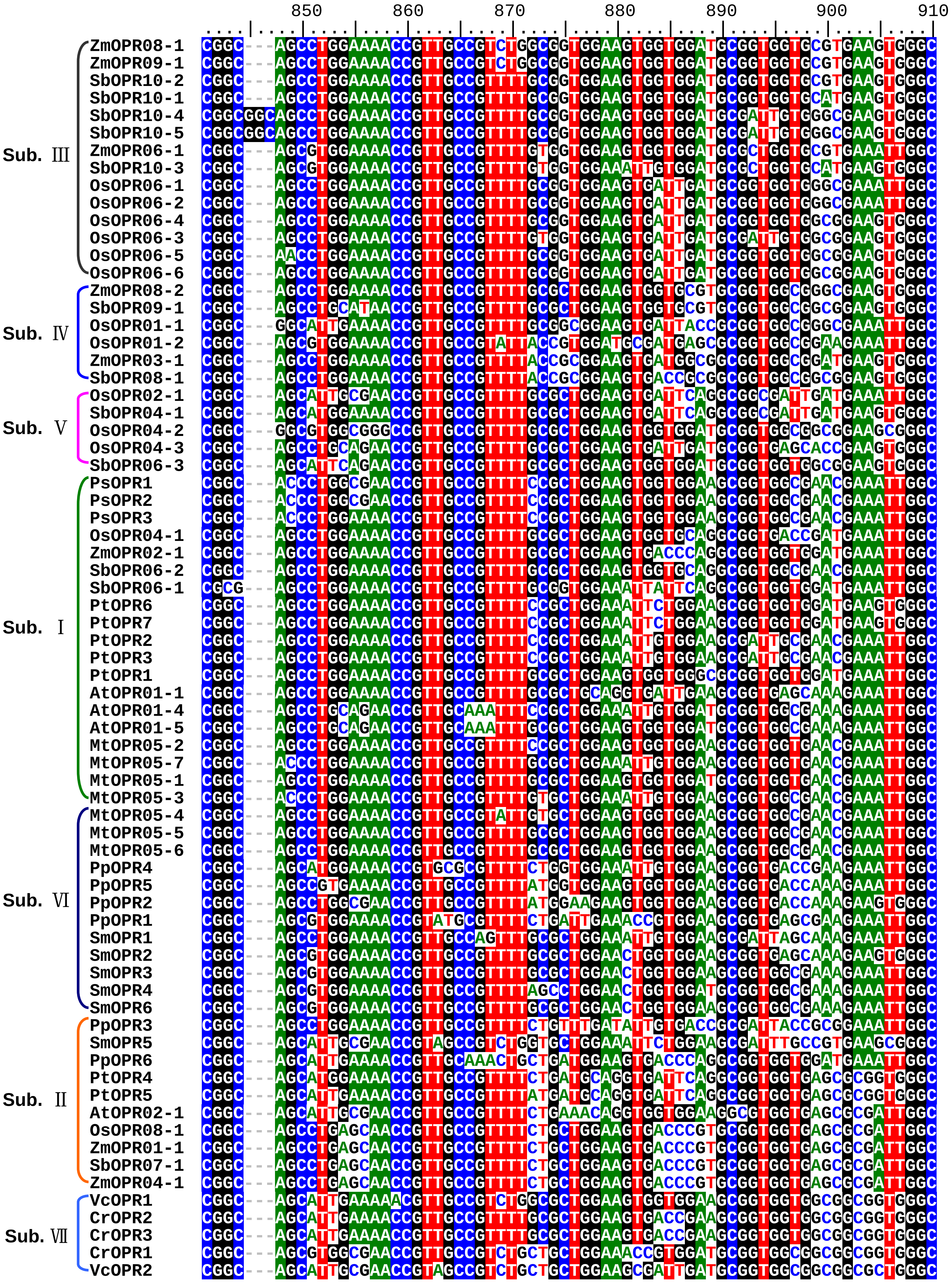
850
860
870
880
890
900
910
Sub. Ⅲ
ZmOPR08-1
ZmOPR09-1
SbOPR10-2
SbOPR10-1
SbOPR10-4
SbOPR10-5
ZmOPR06-1
SbOPR10-3
OsOPR06-1
OsOPR06-2
OsOPR06-4
OsOPR06-3
OsOPR06-5
OsOPR06-6
Sub. Ⅳ
ZmOPR08-2
SbOPR09-1
OsOPR01-1
OsOPR01-2
ZmOPR03-1
SbOPR08-1
Sub. Ⅴ
OsOPR02-1
SbOPR04-1
OsOPR04-2
OsOPR04-3
SbOPR06-3
Sub. Ⅰ
PsOPR1
PsOPR2
PsOPR3
OsOPR04-1
ZmOPR02-1
SbOPR06-2
SbOPR06-1
PtOPR6
PtOPR7
PtOPR2
PtOPR3
PtOPR1
AtOPR01-1
AtOPR01-4
AtOPR01-5
MtOPR05-2
MtOPR05-7
MtOPR05-1
MtOPR05-3
Sub. Ⅵ
MtOPR05-4
MtOPR05-5
MtOPR05-6
PpOPR4
PpOPR5
PpOPR2
PpOPR1
SmOPR1
SmOPR2
SmOPR3
SmOPR4
SmOPR6
Sub. Ⅱ
PpOPR3
SmOPR5
PpOPR6
PtOPR4
PtOPR5
AtOPR02-1
OsOPR08-1
ZmOPR01-1
SbOPR07-1
ZmOPR04-1
Sub. Ⅶ
VcOPR1
CrOPR2
CrOPR3
CrOPR1
VcOPR2

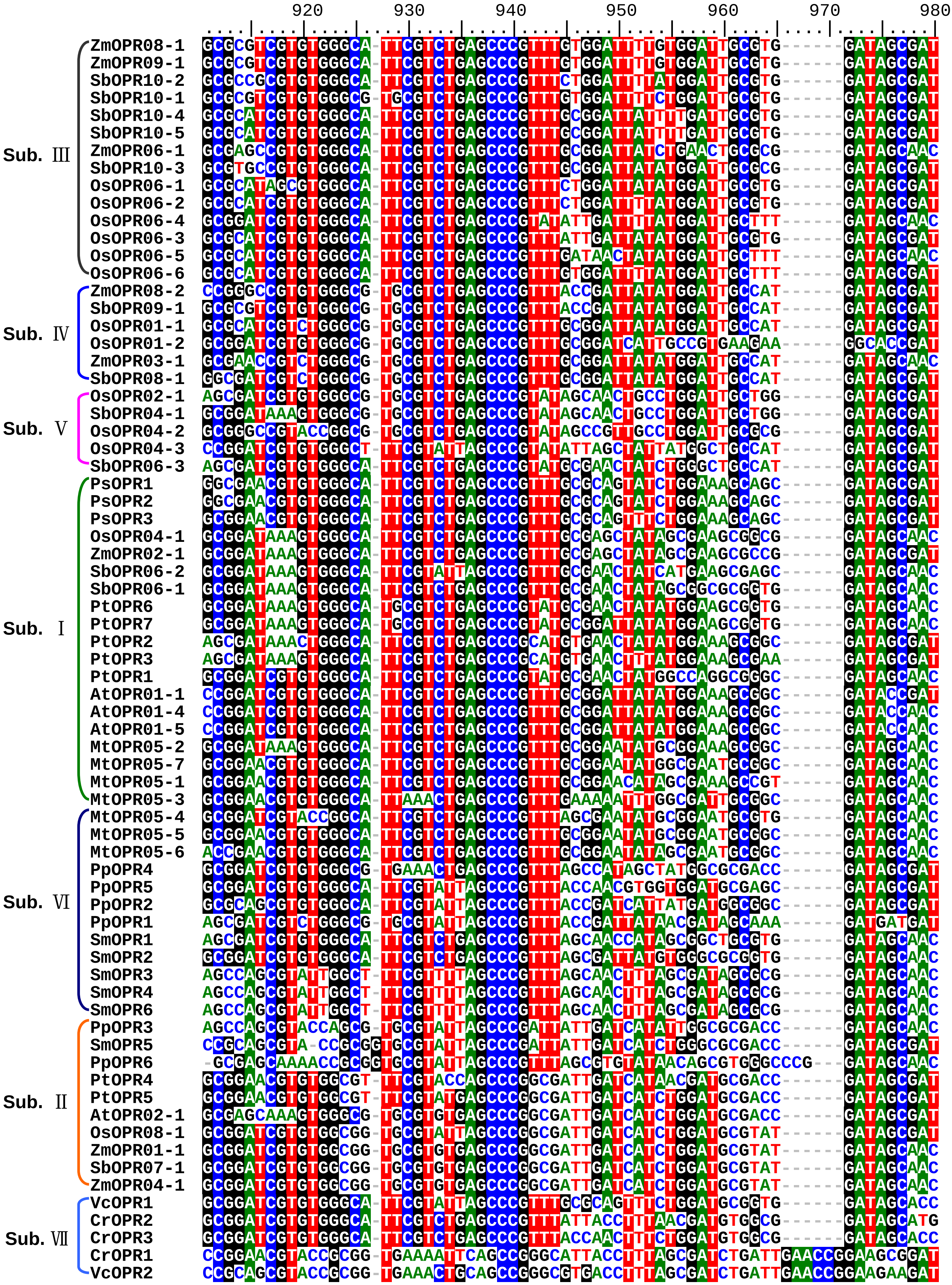
920
930
940
950
960
970
980
Sub. Ⅲ
ZmOPR08-1
ZmOPR09-1
SbOPR10-2
SbOPR10-1
SbOPR10-4
SbOPR10-5
ZmOPR06-1
SbOPR10-3
OsOPR06-1
OsOPR06-2
OsOPR06-4
OsOPR06-3
OsOPR06-5
OsOPR06-6
Sub. Ⅳ
ZmOPR08-2
SbOPR09-1
OsOPR01-1
OsOPR01-2
ZmOPR03-1
SbOPR08-1
Sub. Ⅴ
OsOPR02-1
SbOPR04-1
OsOPR04-2
OsOPR04-3
SbOPR06-3
Sub. Ⅰ
PsOPR1
PsOPR2
PsOPR3
OsOPR04-1
ZmOPR02-1
SbOPR06-2
SbOPR06-1
PtOPR6
PtOPR7
PtOPR2
PtOPR3
PtOPR1
AtOPR01-1
AtOPR01-4
AtOPR01-5
MtOPR05-2
MtOPR05-7
MtOPR05-1
MtOPR05-3
Sub. Ⅵ
MtOPR05-4
MtOPR05-5
MtOPR05-6
PpOPR4
PpOPR5
PpOPR2
PpOPR1
SmOPR1
SmOPR2
SmOPR3
SmOPR4
SmOPR6
Sub. Ⅱ
PpOPR3
SmOPR5
PpOPR6
PtOPR4
PtOPR5
AtOPR02-1
OsOPR08-1
ZmOPR01-1
SbOPR07-1
ZmOPR04-1
Sub. Ⅶ
VcOPR1
CrOPR2
CrOPR3
CrOPR1
VcOPR2

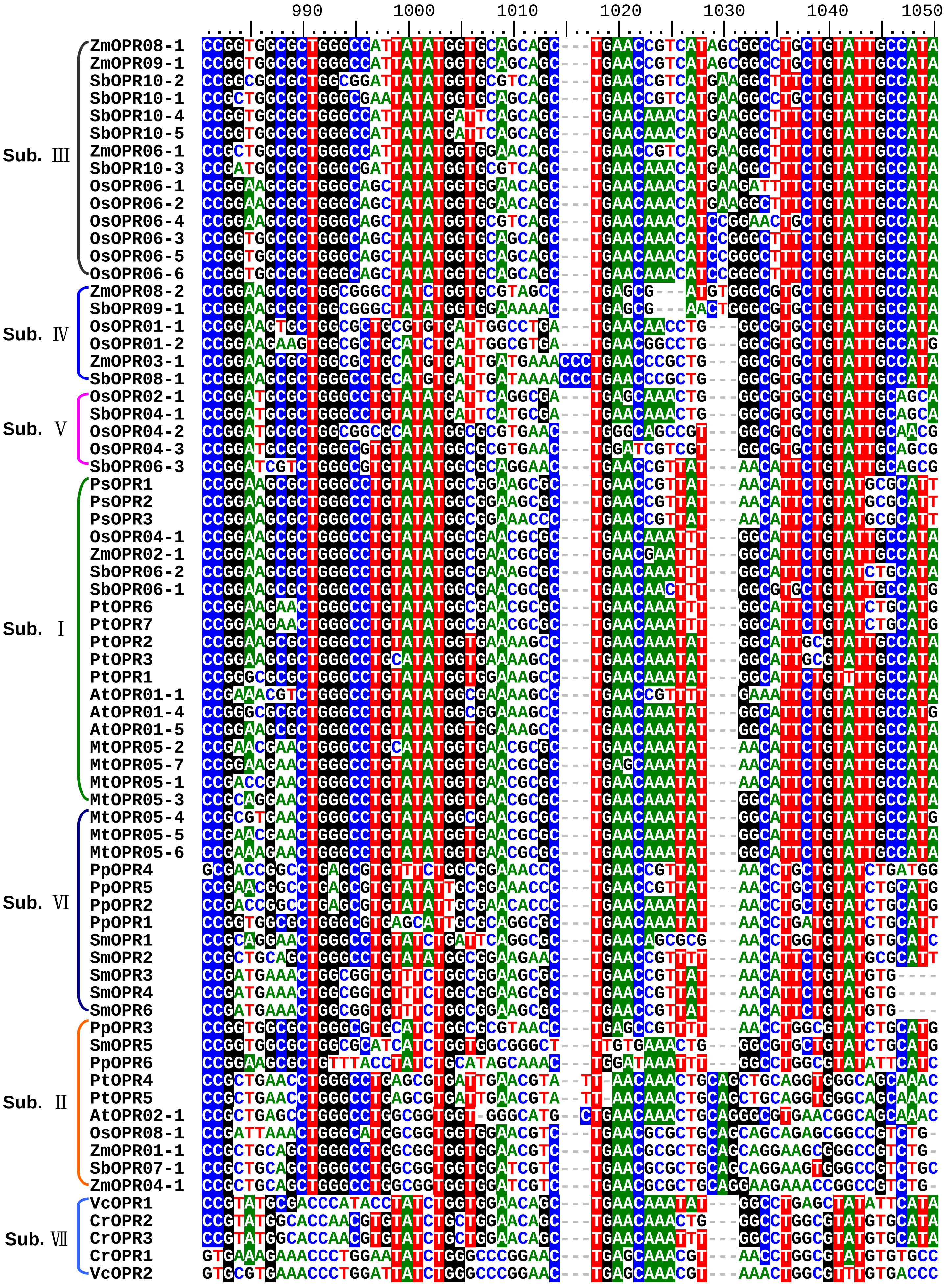
990
1000
1010
1020
1030
1040
1050
Sub. Ⅲ
ZmOPR08-1
ZmOPR09-1
SbOPR10-2
SbOPR10-1
SbOPR10-4
SbOPR10-5
ZmOPR06-1
SbOPR10-3
OsOPR06-1
OsOPR06-2
OsOPR06-4
OsOPR06-3
OsOPR06-5
OsOPR06-6
Sub. Ⅳ
ZmOPR08-2
SbOPR09-1
OsOPR01-1
OsOPR01-2
ZmOPR03-1
SbOPR08-1
Sub. Ⅴ
OsOPR02-1
SbOPR04-1
OsOPR04-2
OsOPR04-3
SbOPR06-3
Sub. Ⅰ
PsOPR1
PsOPR2
PsOPR3
OsOPR04-1
ZmOPR02-1
SbOPR06-2
SbOPR06-1
PtOPR6
PtOPR7
PtOPR2
PtOPR3
PtOPR1
AtOPR01-1
AtOPR01-4
AtOPR01-5
MtOPR05-2
MtOPR05-7
MtOPR05-1
MtOPR05-3
Sub. Ⅵ
MtOPR05-4
MtOPR05-5
MtOPR05-6
PpOPR4
PpOPR5
PpOPR2
PpOPR1
SmOPR1
SmOPR2
SmOPR3
SmOPR4
SmOPR6
Sub. Ⅱ
PpOPR3
SmOPR5
PpOPR6
PtOPR4
PtOPR5
AtOPR02-1
OsOPR08-1
ZmOPR01-1
SbOPR07-1
ZmOPR04-1
Sub. Ⅶ
VcOPR1
CrOPR2
CrOPR3
CrOPR1
VcOPR2

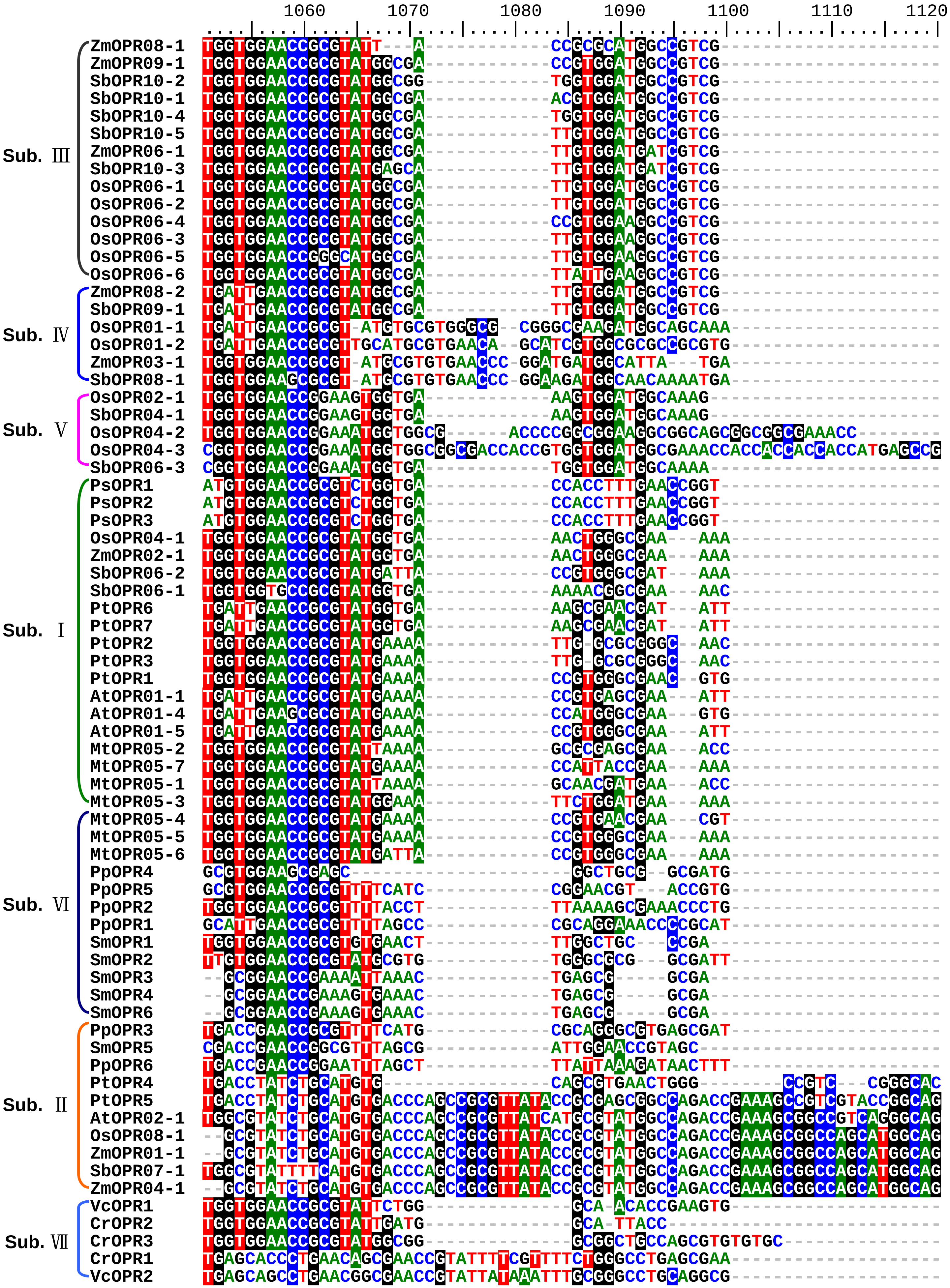
1060
1070
1080
1090
1100
1110
1120
Sub. III
ZmOPR08-1
ZmOPR09-1
SbOPR10-2
SbOPR10-1
SbOPR10-4
SbOPR10-5
ZmOPR06-1
SbOPR10-3
OsOPR06-1
OsOPR06-2
OsOPR06-4
OsOPR06-3
OsOPR06-5
OsOPR06-6
Sub. IV
ZmOPR08-2
SbOPR09-1
OsOPR01-1
OsOPR01-2
ZmOPR03-1
SbOPR08-1
Sub. V
OsOPR02-1
SbOPR04-1
OsOPR04-2
OsOPR04-3
SbOPR06-3
Sub. I
PsOPR1
PsOPR2
PsOPR3
OsOPR04-1
ZmOPR02-1
SbOPR06-2
SbOPR06-1
PtOPR6
PtOPR7
PtOPR2
PtOPR3
PtOPR1
AtOPR01-1
AtOPR01-4
AtOPR01-5
MtOPR05-2
MtOPR05-7
MtOPR05-1
MtOPR05-3
Sub. VI
MtOPR05-4
MtOPR05-5
MtOPR05-6
PpOPR4
PpOPR5
PpOPR2
PpOPR1
SmOPR1
SmOPR2
SmOPR3
SmOPR4
SmOPR6
Sub. II
PpOPR3
SmOPR5
PpOPR6
PtOPR4
PtOPR5
AtOPR02-1
OsOPR08-1
ZmOPR01-1
SbOPR07-1
ZmOPR04-1
Sub. VII
VcOPR1
CrOPR2
CrOPR3
CrOPR1
VcOPR2

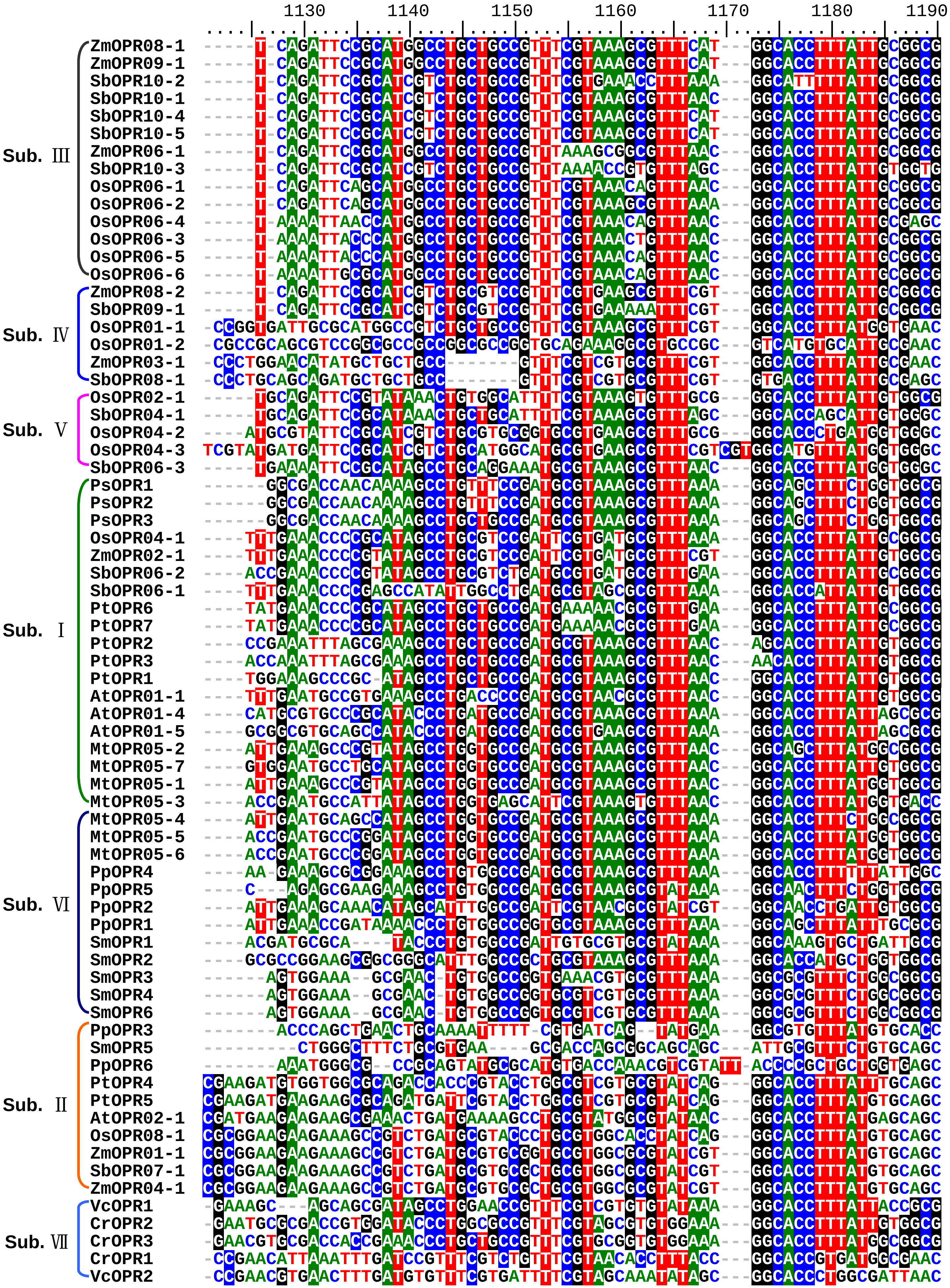
1130
1140
1150
1160
1170
1180
1190
Sub. Ⅲ
ZmOPR08-1
ZmOPR09-1
SbOPR10-2
SbOPR10-1
SbOPR10-4
SbOPR10-5
ZmOPR06-1
SbOPR10-3
OsOPR06-1
OsOPR06-2
OsOPR06-4
OsOPR06-3
OsOPR06-5
OsOPR06-6
Sub. Ⅳ
ZmOPR08-2
SbOPR09-1
OsOPR01-1
OsOPR01-2
ZmOPR03-1
SbOPR08-1
Sub. Ⅴ
OsOPR02-1
SbOPR04-1
OsOPR04-2
OsOPR04-3
SbOPR06-3
Sub. Ⅰ
PsOPR1
PsOPR2
PsOPR3
OsOPR04-1
ZmOPR02-1
SbOPR06-2
SbOPR06-1
PtOPR6
PtOPR7
PtOPR2
PtOPR3
PtOPR1
AtOPR01-1
AtOPR01-4
AtOPR01-5
MtOPR05-2
MtOPR05-7
MtOPR05-1
MtOPR05-3
Sub. Ⅵ
MtOPR05-4
MtOPR05-5
MtOPR05-6
PpOPR4
PpOPR5
PpOPR2
PpOPR1
SmOPR1
SmOPR2
SmOPR3
SmOPR4
SmOPR6
Sub. Ⅱ
PpOPR3
SmOPR5
PpOPR6
PtOPR4
PtOPR5
AtOPR02-1
OsOPR08-1
ZmOPR01-1
SbOPR07-1
ZmOPR04-1
Sub. Ⅶ
VcOPR1
CrOPR2
CrOPR3
CrOPR1
VcOPR2

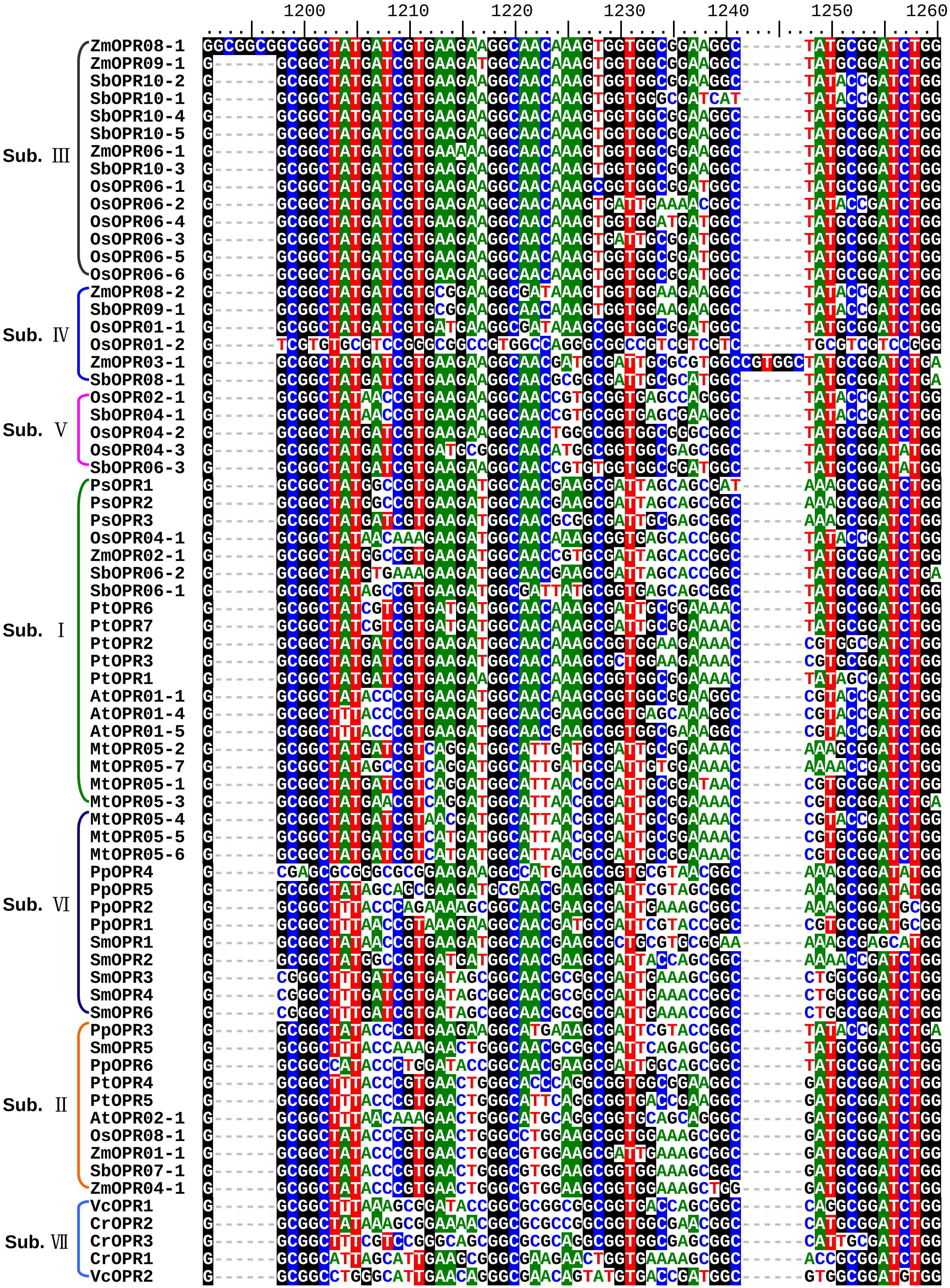
1200
1210
1220
1230
1240
1250
1260
Sub. III
ZmOPR08-1
ZmOPR09-1
SbOPR10-2
SbOPR10-1
SbOPR10-4
SbOPR10-5
ZmOPR06-1
SbOPR10-3
OsOPR06-1
OsOPR06-2
OsOPR06-4
OsOPR06-3
OsOPR06-5
OsOPR06-6
Sub. IV
ZmOPR08-2
SbOPR09-1
OsOPR01-1
OsOPR01-2
ZmOPR03-1
SbOPR08-1
Sub. V
OsOPR02-1
SbOPR04-1
OsOPR04-2
OsOPR04-3
SbOPR06-3
Sub. I
PsOPR1
PsOPR2
PsOPR3
OsOPR04-1
ZmOPR02-1
SbOPR06-2
SbOPR06-1
PtOPR6
PtOPR7
PtOPR2
PtOPR3
PtOPR1
AtOPR01-1
AtOPR01-4
AtOPR01-5
MtOPR05-2
MtOPR05-7
MtOPR05-1
MtOPR05-3
Sub. VI
MtOPR05-4
MtOPR05-5
MtOPR05-6
PpOPR4
PpOPR5
PpOPR2
PpOPR1
SmOPR1
SmOPR2
SmOPR3
SmOPR4
SmOPR6
Sub. II
PpOPR3
SmOPR5
PpOPR6
PtOPR4
PtOPR5
AtOPR02-1
OsOPR08-1
ZmOPR01-1
SbOPR07-1
ZmOPR04-1
Sub. VII
VcOPR1
CrOPR2
CrOPR3
CrOPR1
VcOPR2

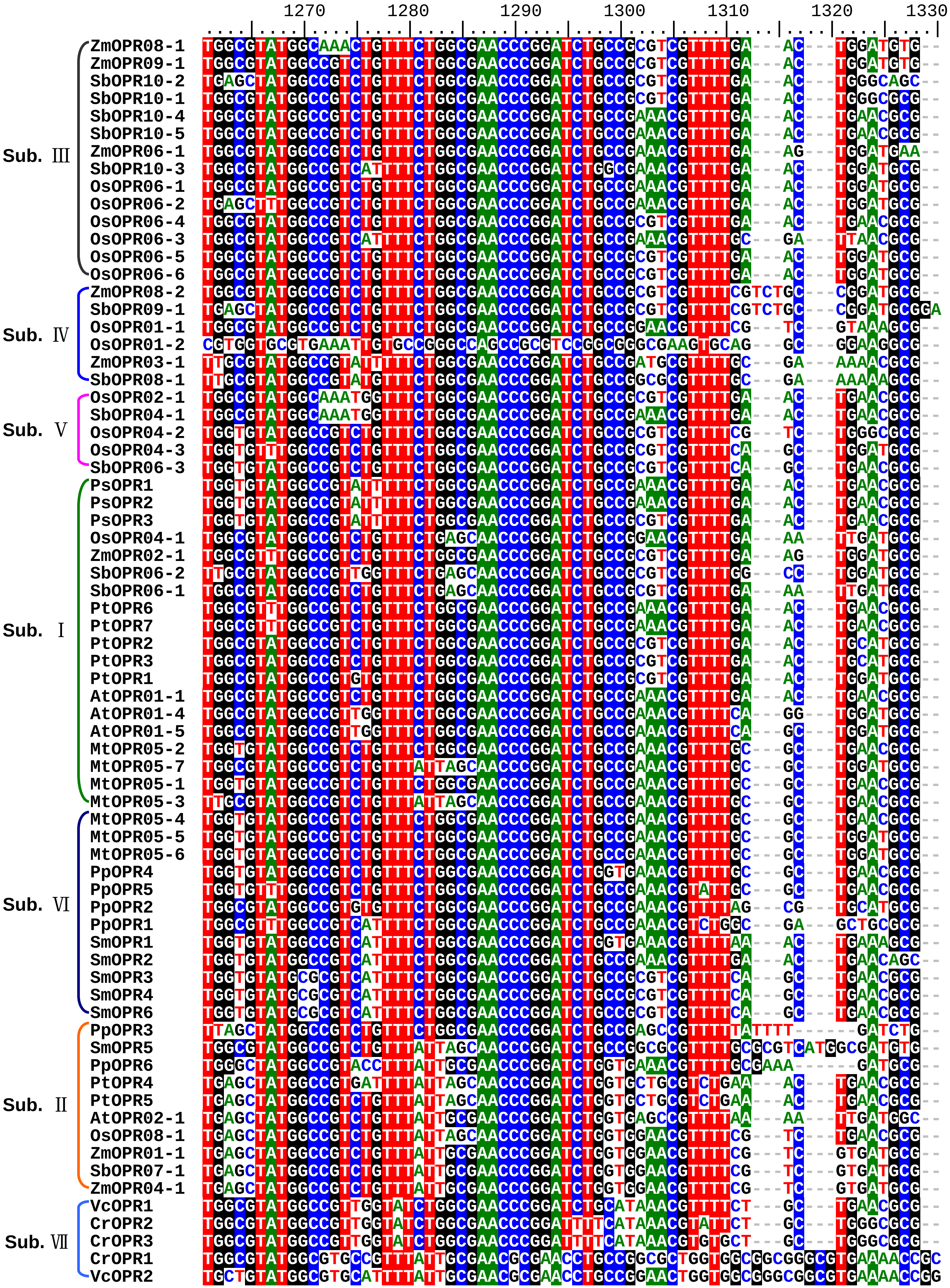
1270
1280
1290
1300
1310
1320
1330
Sub. Ⅲ
ZmOPR08-1
ZmOPR09-1
SbOPR10-2
SbOPR10-1
SbOPR10-4
SbOPR10-5
ZmOPR06-1
SbOPR10-3
OsOPR06-1
OsOPR06-2
OsOPR06-4
OsOPR06-3
OsOPR06-5
OsOPR06-6
Sub. Ⅳ
ZmOPR08-2
SbOPR09-1
OsOPR01-1
OsOPR01-2
ZmOPR03-1
SbOPR08-1
Sub. Ⅴ
OsOPR02-1
SbOPR04-1
OsOPR04-2
OsOPR04-3
SbOPR06-3
Sub. Ⅰ
PsOPR1
PsOPR2
PsOPR3
OsOPR04-1
ZmOPR02-1
SbOPR06-2
SbOPR06-1
PtOPR6
PtOPR7
PtOPR2
PtOPR3
PtOPR1
AtOPR01-1
AtOPR01-4
AtOPR01-5
MtOPR05-2
MtOPR05-7
MtOPR05-1
MtOPR05-3
Sub. Ⅵ
MtOPR05-4
MtOPR05-5
MtOPR05-6
PpOPR4
PpOPR5
PpOPR2
PpOPR1
SmOPR1
SmOPR2
SmOPR3
SmOPR4
SmOPR6
Sub. Ⅱ
PpOPR3
SmOPR5
PpOPR6
PtOPR4
PtOPR5
AtOPR02-1
OsOPR08-1
ZmOPR01-1
SbOPR07-1
ZmOPR04-1
Sub. Ⅶ
VcOPR1
CrOPR2
CrOPR3
CrOPR1
VcOPR2

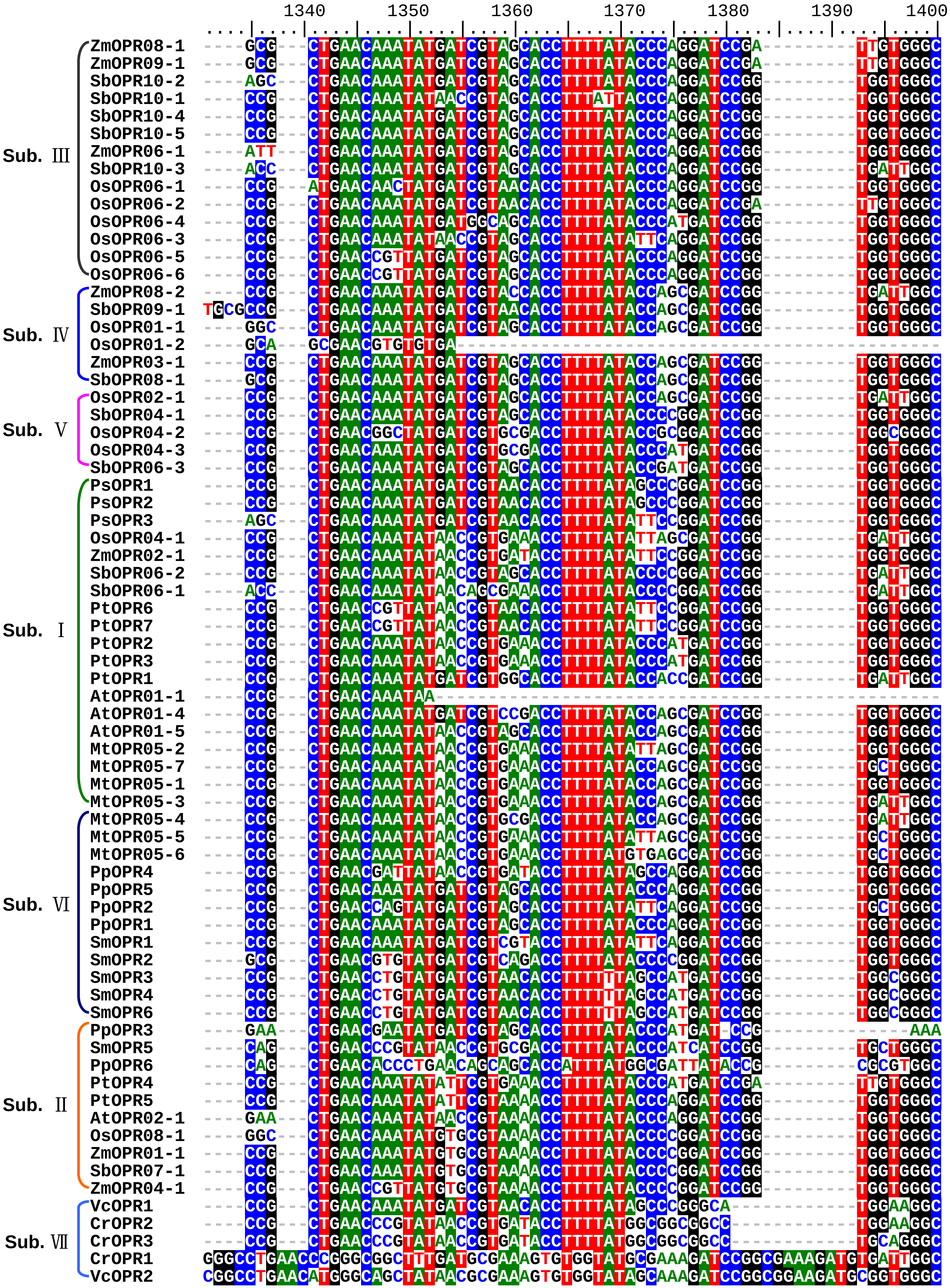

1340
1350
1360
1370
1380
1390
1400
Sub. Ⅲ
ZmOPR08-1
ZmOPR09-1
SbOPR10-2
SbOPR10-1
SbOPR10-4
SbOPR10-5
ZmOPR06-1
SbOPR10-3
OsOPR06-1
OsOPR06-2
OsOPR06-4
OsOPR06-3
OsOPR06-5
OsOPR06-6
Sub. Ⅳ
ZmOPR08-2
SbOPR09-1
OsOPR01-1
OsOPR01-2
ZmOPR03-1
SbOPR08-1
Sub. Ⅴ
OsOPR02-1
SbOPR04-1
OsOPR04-2
OsOPR04-3
SbOPR06-3
Sub. Ⅰ
PsOPR1
PsOPR2
PsOPR3
OsOPR04-1
ZmOPR02-1
SbOPR06-2
SbOPR06-1
PtOPR6
PtOPR7
PtOPR2
PtOPR3
PtOPR1
AtOPR01-1
AtOPR01-4
AtOPR01-5
MtOPR05-2
MtOPR05-7
MtOPR05-1
MtOPR05-3
Sub. Ⅵ
MtOPR05-4
MtOPR05-5
MtOPR05-6
PpOPR4
PpOPR5
PpOPR2
PpOPR1
SmOPR1
SmOPR2
SmOPR3
SmOPR4
SmOPR6
Sub. Ⅱ
PpOPR3
SmOPR5
PpOPR6
PtOPR4
PtOPR5
AtOPR02-1
OsOPR08-1
ZmOPR01-1
SbOPR07-1
ZmOPR04-1
Sub. Ⅶ
VcOPR1
CrOPR2
CrOPR3
CrOPR1
VcOPR2

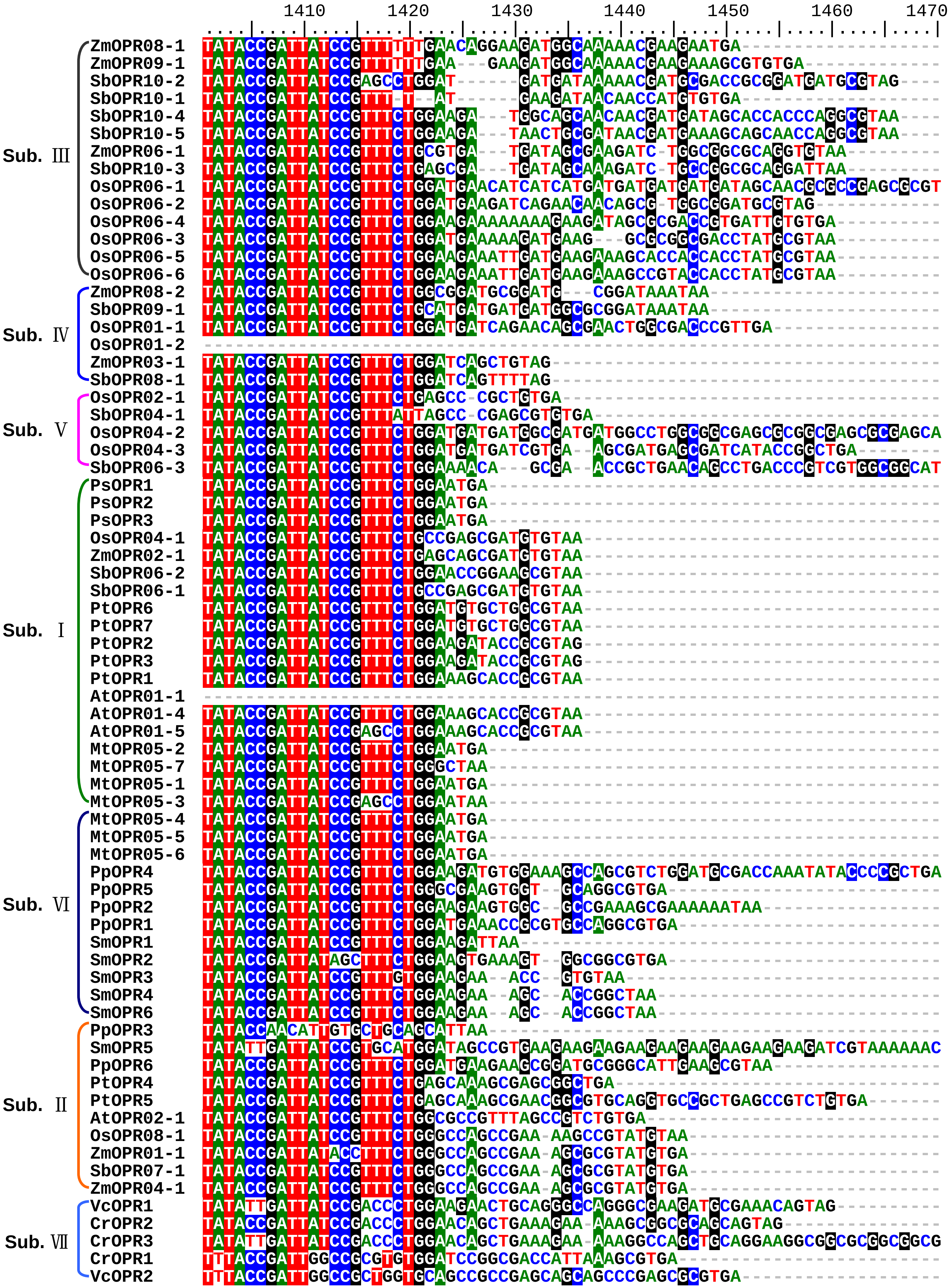
1410
1420
1430
1440
1450
1460
1470
Sub. III
ZmOPR08-1
ZmOPR09-1
SbOPR10-2
SbOPR10-1
SbOPR10-4
SbOPR10-5
ZmOPR06-1
SbOPR10-3
OsOPR06-1
OsOPR06-2
OsOPR06-4
OsOPR06-3
OsOPR06-5
OsOPR06-6
Sub. IV
ZmOPR08-2
SbOPR09-1
OsOPR01-1
OsOPR01-2
ZmOPR03-1
SbOPR08-1
Sub. V
OsOPR02-1
SbOPR04-1
OsOPR04-2
OsOPR04-3
SbOPR06-3
Sub. I
PsOPR1
PsOPR2
PsOPR3
OsOPR04-1
ZmOPR02-1
SbOPR06-2
SbOPR06-1
PtOPR6
PtOPR7
PtOPR2
PtOPR3
PtOPR1
AtOPR01-1
AtOPR01-4
AtOPR01-5
MtOPR05-2
MtOPR05-7
MtOPR05-1
MtOPR05-3
Sub. VI
MtOPR05-4
MtOPR05-5
MtOPR05-6
PpOPR4
PpOPR5
PpOPR2
PpOPR1
SmOPR1
SmOPR2
SmOPR3
SmOPR4
SmOPR6
Sub. II
PpOPR3
SmOPR5
PpOPR6
PtOPR4
PtOPR5
AtOPR02-1
OsOPR08-1
ZmOPR01-1
SbOPR07-1
ZmOPR04-1
Sub. VII
VcOPR1
CrOPR2
CrOPR3
CrOPR1
VcOPR2

```
                              1480      1490      1500      1510
                          ....|....|....|....|....|....|....|....|....
Sub. III    ZmOPR08-1     --------------------------------------------
            ZmOPR09-1     --------------------------------------------
            SbOPR10-2     --------------------------------------------
            SbOPR10-1     --------------------------------------------
            SbOPR10-4     --------------------------------------------
            SbOPR10-5     --------------------------------------------
            ZmOPR06-1     --------------------------------------------
            SbOPR10-3     --------------------------------------------
            OsOPR06-1     AA------------------------------------------
            OsOPR06-2     --------------------------------------------
            OsOPR06-4     --------------------------------------------
            OsOPR06-3     --------------------------------------------
            OsOPR06-5     --------------------------------------------
            OsOPR06-6     --------------------------------------------
Sub. IV     ZmOPR08-2     --------------------------------------------
            SbOPR09-1     --------------------------------------------
            OsOPR01-1     --------------------------------------------
            OsOPR01-2     --------------------------------------------
            ZmOPR03-1     --------------------------------------------
            SbOPR08-1     --------------------------------------------
Sub. V      OsOPR02-1     --------------------------------------------
            SbOPR04-1     --------------------------------------------
            OsOPR04-2     GCAACAAAAGCGGCGATCAGGATGGCGTGTAG------------
            OsOPR04-3     --------------------------------------------
            SbOPR06-3     TTATCAGAACAAAGAAGGCATGATTTGA----------------
Sub. I      PsOPR1        --------------------------------------------
            PsOPR2        --------------------------------------------
            PsOPR3        --------------------------------------------
            OsOPR04-1     --------------------------------------------
            ZmOPR02-1     --------------------------------------------
            SbOPR06-2     --------------------------------------------
            SbOPR06-1     --------------------------------------------
            PtOPR6        --------------------------------------------
            PtOPR7        --------------------------------------------
            PtOPR2        --------------------------------------------
            PtOPR3        --------------------------------------------
            PtOPR1        --------------------------------------------
            AtOPR01-1     --------------------------------------------
            AtOPR01-4     --------------------------------------------
            AtOPR01-5     --------------------------------------------
            MtOPR05-2     --------------------------------------------
            MtOPR05-7     --------------------------------------------
            MtOPR05-1     --------------------------------------------
            MtOPR05-3     --------------------------------------------
Sub. VI     MtOPR05-4     --------------------------------------------
            MtOPR05-5     --------------------------------------------
            MtOPR05-6     --------------------------------------------
            PpOPR4        ACACCAAAATTGCGGAATTTTGCAGCCGTGCGACCGTGCTGTGA
            PpOPR5        --------------------------------------------
            PpOPR2        --------------------------------------------
            PpOPR1        --------------------------------------------
            SmOPR1        --------------------------------------------
            SmOPR2        --------------------------------------------
            SmOPR3        --------------------------------------------
            SmOPR4        --------------------------------------------
            SmOPR6        --------------------------------------------
Sub. II     PpOPR3        --------------------------------------------
            SmOPR5        ATCCGAGCAAACTGTAG---------------------------
            PpOPR6        --------------------------------------------
            PtOPR4        --------------------------------------------
            PtOPR5        --------------------------------------------
            AtOPR02-1     --------------------------------------------
            OsOPR08-1     --------------------------------------------
            ZmOPR01-1     --------------------------------------------
            SbOPR07-1     --------------------------------------------
            ZmOPR04-1     --------------------------------------------
Sub. VII    VcOPR1        --------------------------------------------
            CrOPR2        --------------------------------------------
            CrOPR3        GTGACCGATAAATAG-----------------------------
            CrOPR1        --------------------------------------------
            VcOPR2        --------------------------------------------
```

Additional file 4

Nucleotide sequences alignment of OPR genes from eleven species representing the six major lineages within the green plants: the green algae (*Chlamydomonas reinhardtii* and *Volvox carteri*), the mosses (*Physcomitrella patens*), the lycophytes (*Selaginella moellendorffii*), the gymnosperms (*Picea sitchensis*), the monocotyledoneous angiosperms (*Oryza sativa*, *Sorghum bicolor* and *Zea mays*) and the dicotyledoneous angiosperms (*Arabidopsis thaliana*, *Populus trichocarpa* and *Medicago truncatula*). Identical residues in the alignments are shaded and seven well-conserved OPR subfamilies are represented as Sub. Ⅰ, Ⅱ, Ⅲ, Ⅳ, Ⅴ, Ⅵ and Ⅶ at the left side of the alignment.
