# Supplementary material for: Phylogenetic analysis, structural evolution and functional divergence of the 12-oxo-phytodienoate acid reductase gene family in plants
Source: BMC Evol Biol. 2009 May 5;9:90. doi: 10.1186/1471-2148-9-90 (PMC2688005; doi:10.1186/1471-2148-9-90)
Supplement: Additional file 5 — The rooted maximum-likelihood phylogenetic tree of OPR family genes from eleven representative species. The rooted maximum-likelihood (ML) phylogenetic tree of OPR genes in eleven representative plants was inferred from the amino acid sequence alignments of the Oxidored_FMN domain (Additional file 3) under the Jones-Taylor-Thornton (JTT) model, implemented in Phylip v3.68 package. The old yellow enzyme 1 (OYE1) of Saccharomyces cerevisiae (ScOYE1) was selected as an out of group marker and its accession number in SWISS-PROT is Q02899. The bootstrap values from 1000 resamplings are given at each node and the branch lengths are drawn to scale. The seven conserved OPR subfamilies are represented as Sub.I, II, III, IV, V, VI and VII at the right side of the ML tree. [file 1471-2148-9-90-S5.pdf]

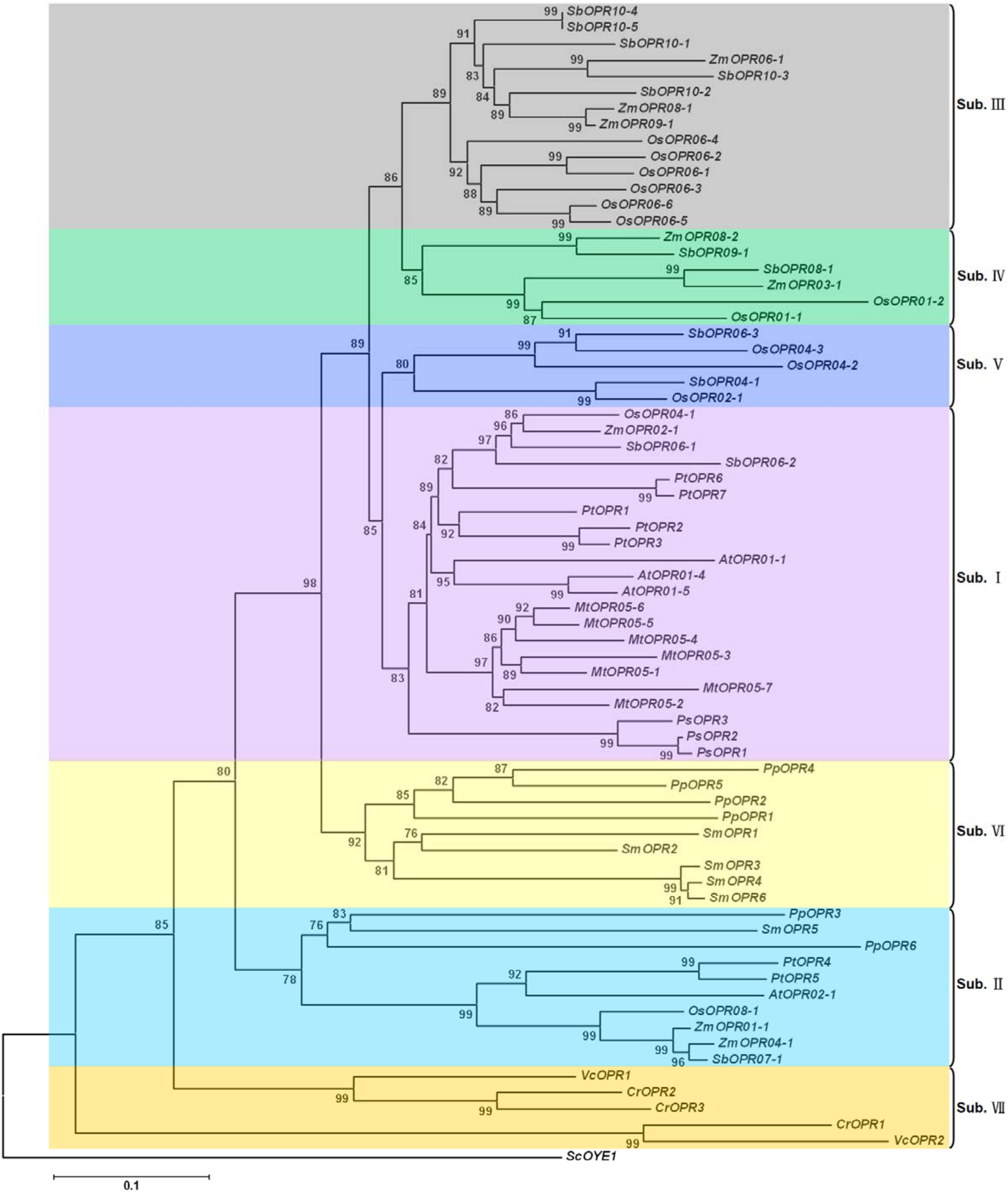

SbOPR10-4
SbOPR10-5
SbOPR10-1
ZmOPR06-1
SbOPR10-3
SbOPR10-2
ZmOPR08-1
ZmOPR09-1
OsOPR06-4
OsOPR06-2
OsOPR06-1
OsOPR06-3
OsOPR06-6
OsOPR06-5
ZmOPR08-2
SbOPR09-1
SbOPR08-1
ZmOPR03-1
OsOPR01-2
OsOPR01-1
SbOPR06-3
OsOPR04-3
OsOPR04-2
SbOPR04-1
OsOPR02-1
OsOPR04-1
ZmOPR02-1
SbOPR06-1
SbOPR06-2
PtOPR6
PtOPR7
PtOPR1
PtOPR2
PtOPR3
AtOPR01-1
AtOPR01-4
AtOPR01-5
MtOPR05-6
MtOPR05-5
MtOPR05-4
MtOPR05-3
MtOPR05-1
MtOPR05-7
MtOPR05-2
PsOPR3
PsOPR2
PsOPR1
PpOPR4
PpOPR5
PpOPR2
PpOPR1
SmOPR1
SmOPR2
SmOPR3
SmOPR4
SmOPR6
PpOPR3
SmOPR5
PpOPR6
PtOPR4
PtOPR5
AtOPR02-1
OsOPR08-1
ZmOPR01-1
ZmOPR04-1
SbOPR07-1
VcOPR1
CrOPR2
CrOPR3
CrOPR1
VcOPR2
ScOYE1
Sub. III
Sub. IV
Sub. V
Sub. I
Sub. VI
Sub. II
Sub. VII
0.1

Additional file 5

The rooted maximum-likelihood (ML) phylogenetic tree of OPR genes in 11 representative plants was inferred from the amino acid sequence alignments of the Oxidored_FMN domain (Fig. S1) under the Jones-Taylor-Thornton (JTT) model (Jones et al., 1992), implemented in Phylip v3.68 package (Retief, 2000). The old yellow enzyme 1 (OYE1) of *Saccharomyces cerevisiae* (*ScOYE1*) (Saito et al., 1991) was selected as an out of group marker and its accession number in SWISS-PROT is Q02899. The bootstrap values from 1000 resamplings are given at each node and the branch lengths are drawn to scale. The seven conserved OPR subfamilies are represented as Sub. Ⅰ, Ⅱ, Ⅲ, Ⅳ, Ⅴ, Ⅵ and Ⅶ at the right side of the ML tree.

References

Jones DT, Taylor WR, Thornton JM (1992) The rapid generation of mutation data matrices from protein sequences. Comput Appl Biosci 8: 275-282

Retief JD (2000) Phylogenetic analysis using PHYLIP. Methods Mol Biol 132: 243-258

Saito K, Thiele DJ, Davio M, Lockridge O, Massey V (1991) The cloning and expression of a gene encoding Old Yellow Enzyme from Saccharomyces carlsbergensis. J Biol Chem 266: 20720-20724
