# Supplementary material for: Phylogenetic analysis, structural evolution and functional divergence of the 12-oxo-phytodienoate acid reductase gene family in plants
Source: BMC Evol Biol. 2009 May 5;9:90. doi: 10.1186/1471-2148-9-90 (PMC2688005; doi:10.1186/1471-2148-9-90)
Supplement: Additional file 6 — Likelihood ratio statistics (2Δℓ). This data provide represent the likelihood ratio statistical analysis of the comparisons of M0 with M3, M1a with M2a, and M7 with M8, respectively. [file 1471-2148-9-90-S6.pdf]

Additional file 6

Likelihood ratio statistics (2Δℓ)

Comparison	2Δℓ	df	$\chi^2_{1\%}$
M0 (one-ratio) vs. M3 (discrete)	2439.82	4	13.28
M1a (nearly neutral) vs. M2a (positive selection)	807.50	2	9.21
M7 (beta) vs. M8 (beta & ω)	673.90	2	9.21
